# A novel genomic signature predicting FDG uptake in diverse metastatic tumors

**DOI:** 10.1186/s13550-017-0355-3

**Published:** 2018-01-18

**Authors:** Aurora Crespo-Jara, Maria Carmen Redal-Peña, Elena Maria Martinez-Navarro, Manuel Sureda, Francisco Jose Fernandez-Morejon, Francisco J. Garcia-Cases, Ramon Gonzalez Manzano, Antonio Brugarolas

**Affiliations:** 1 Plataforma de Oncologia, Hospital Quironsalud Torrevieja, Pda. La Loma s/n, 03184 Torrevieja, Alicante Spain; 2 Catedra Oncologia Multidisciplinar, Universidad Catolica de Murcia, Murcia, Spain

**Keywords:** FDG uptake, SUV, Metastatic cancer, Genomic signature, Gene expression microarray

## Abstract

**Background:**

Building a universal genomic signature predicting the intensity of FDG uptake in diverse metastatic tumors may allow us to understand better the biological processes underlying this phenomenon and their requirements of glucose uptake.

**Methods:**

A balanced training set (*n* = 71) of metastatic tumors including some of the most frequent histologies, with matched PET/CT quantification measurements and whole human genome gene expression microarrays, was used to build the signature. Selection of microarray features was carried out exclusively on the basis of their strong association with FDG uptake (as measured by SUVmean35) by means of univariate linear regression. A thorough bioinformatics study of these genes was performed, and multivariable models were built by fitting several state of the art regression techniques to the training set for comparison.

**Results:**

The 909 probes with the strongest association with the SUVmean35 (comprising 742 identifiable genes and 62 probes not matched to a symbol) were used to build the signature. Partial least squares using three components (PLS-3) was the best performing model in the training dataset cross-validation (root mean square error, RMSE = 0.443) and was validated further in an independent validation dataset (*n* = 13) obtaining a performance within the 95% CI of that obtained in the training dataset (RMSE = 0.645). Significantly overrepresented biological processes correlating with the SUVmean35 were identified beyond glycolysis, such as ribosome biogenesis and DNA replication (correlating with a higher SUVmean35) and cytoskeleton reorganization and autophagy (correlating with a lower SUVmean35).

**Conclusions:**

PLS-3 is a signature predicting accurately the intensity of FDG uptake in diverse metastatic tumors. FDG-PET might help in the design of specific targeted therapies directed to counteract the identified malignant biological processes more likely activated in a tumor as inferred from the SUVmean35 and also from its variations in response to antineoplastic treatments.

**Electronic supplementary material:**

The online version of this article (10.1186/s13550-017-0355-3) contains supplementary material, which is available to authorized users.

## Background

2 [18F] Fluoro-2-deoxy-D-glucose (FDG) positron emission tomography (PET) is a metabolic imaging technique commonly used in the clinic to evaluate the extension of primary or metastatic tumors prior to therapy. Another use of this technique that is gaining more acceptance in oncology is the assessment of early metabolic response to antineoplastic agents in advanced and metastatic tumors [1, 2].

At the molecular level, FDG uptake has been related mainly to aerobic glycolysis, but a full picture of the different biological pathways involved in this process is currently lacking. While the core molecular machinery of glycolysis is widespread in all tumors, the intensity of FDG uptake is quite variable among different tumor histologies and even among the same tumor histotypes according to specific tumor characteristics [3, 4]. Some studies have correlated the tumor FDG uptake with the expression of essential glycolytic enzymes such as hexokinase-2 or related proteins like glucose transporters Glut1-3 [5–9]. However, a good correlation between these biomarkers and the intensity of FDG uptake is not always found in all tumor types [9]. Other preclinical studies in cancer cell lines have also shown that other biological processes can also be concomitantly upregulated in the presence of higher uptake of FDG in tumors, as it happens in the activation of oncogenic pathways such as KRAS, PI3K, and c-MYC [10–12]. All these studies have focused in a limited number of selected genes and in specific tumor types, gathering thus a limited view of the biology of FDG uptake.

As metastases are the main cause of cancer-related death, a growing interest in metastatic cancer has been recently spurred on by a more thorough characterization of the genomic landscape of these tumors [13]. Hence, we reasoned that a better understanding of the biological processes involved in FDG uptake could be glimpsed by studying a representative sample of diverse human metastatic tumors, accounting thus for a greater tumor heterogeneity but retaining a number of common processes underlying the biology of FDG uptake beyond glycolysis.

The purpose of the present study was to build a genomic signature able to predict FDG uptake intensity in a diverse population of metastatic tumors, by using an unbiased gene expression profiling not limited to a predefined set of genes, but rather using whole human genome gene expression microarrays. To achieve this goal, a different methodology from that used previously in other signatures, that were trained on a single tumor type [14, 15], was required. Individual genes were selected exclusively by their strong association with FDG uptake by means of univariate linear regression. Then, these selected genomic features were used to build and validate the signature, chosen by comparing several state of the art predictive regression methods. The selected genes would also allow us to deepen into the overrepresented biological processes and signaling pathways common to glucose uptake in different metastatic tumors, as well as into the potential protein-protein interaction (PPI) subnetworks found among the selected features.

A deeper knowledge of the metabolic pathways beyond glycolysis involved in FDG uptake might contribute to establish the usefulness of FDG PET/CT in indications such as the evaluation of early metabolic response with different targeted therapies.

## Patients and Methods

### Inclusion criteria

The conditions that patients should meet to enter this study were (a) a diagnosis of metastatic tumor (all were solid except a single patient with non-Hodgkin lymphoma) with a baseline FDG-PET/CT in order to evaluate the extent of disease and at a later point treatment response, (b) a fresh frozen tumor biopsy taken at the same metastatic location in which FDG uptake was measured for a gene expression microarray, that was performed within a maximum interval of 8 weeks of the FDG-PET/CT, (c) the patients had not received chemotherapy treatment in the 3 weeks prior to the inclusion in the study, and (d) patients in whom no active tumor could be identified by FDG-PET were excluded from the study. Seventy-one cancer patients, seen between July 2010 and July 2015 at Hospital Quironsalud Torrevieja (Alicante, Spain), met these requirements and were retrospectively evaluated. In 3 of these patients, more than one microarray studies had been performed several months apart, but only the first one was included in this study. No other restrictions applied to the patients entering the study on the basis of sex, age, histology of the tumor, or previous treatments. Informed consents for the obtention of the diagnostic-therapeutic biopsy and for undergoing FDG-PET/CT in the patients included in this study were obtained. Approval of this study by the Institutional Review Board of Hospital Quironsalud Torrevieja (Alicante, Spain) was also obtained.

These 71 patients comprised the training set used to build the predictive genomic signature. A balanced proportion of some of the most frequent tumor histologies (eight tumor types comprising from 5 to 9 patients) along with a group of miscellaneous tumor types constituted this training set (see Table 2). The hypothesis made was that we would be able to capture the underlying common biological processes related to the intensity of FDG uptake shared by different solid tumors by selecting the microarray probes most strongly correlated with FDG uptake (by means of univariate linear regression) in order to build a predictive signature (see Additional file 1 for full details). Besides, 14 additional patients were seen at our institution after July 2015 and were evaluated prospectively to validate (external validation set) the predictive signature generated with the training set. The signature underwent first an internal validation (tenfold cross-validation × 5 in the training set) to choose the best performing model among the four tested as well as to estimate the performance of the signature in an unseen (by the model) dataset [16] and an external validation in an independent dataset (not used to build the model) to test it further. One patient was excluded from the external validation set as he was considered a clear-cut outlier presenting extremely high values of FDG uptake, as outliers have detrimental effects both in the generation and in the validation of the model. An outlier was defined as those measurement values of FDG uptake that, taking as reference the values of the training set, were either:

≤ P1 − 1.5 × (P3 − P1); or

≥ P3 + 1.5 × (P3 − P1)

where P1 was the 25% percentile and P3 was the 75% percentile.

Among the remaining 13 patients of the validation set, one had a value of FDG uptake just below the patient with the lowest limit in the training set, although it was not a low outlier as defined here. We called this patient sample an “influential observation.” This term is taken from the regression argot and used here with a similar meaning: how different would it be a model prediction if we were to exclude this observation. After logarithmic transformation, this observation was just a little outside the limit of the low outlier boundaries. Nevertheless, in spite of being aware of the limitations of including such a patient (with an uptake value below the prediction range of the training set and a borderline low outlier) for achieving an accurate prediction, we did not exclude her from the validation set in order to study the effect on the predictive accuracy of the signature of excluding this influential observation from the validation set.

### FDG-PET/CT imaging and quantification

All patients fasted for at least 6 h prior to imaging, and pre-examination blood glucose levels were obtained. Patients were injected with 444 MBq (12 mCi) pyrogen-free 18F-FDG. Imaging was performed 90 min (± 10) later on a Biograph 6 Hi-Rez (Siemens Medical Solutions). Whole body PET/CT scanners were acquired in accordance with the HQT PET protocol. CT data was used for attenuation correction (120 mAs Care Dose; 110 Kv, slice 5 mm) and X-ray contrast medium was injected (65 ml ULTRAVIST®, rate 1.6–1.8 ml/s and delay 50 s). All images were iteratively reconstructed using post-emission transmission attenuation-corrected datasets (size 168; zoom 1; full width at half maximum (FHWM) 5.0 mm; iterations 4; subsets 8).

FDG uptake in the biopsied location was quantified. Individual tumor VOIs (volume of interest) were automatically drawn threshold-based, one for each patient. A standard VOI analysis tool provided with the scanner was used to calculate the different quantitative parameters obtained (Leonardo workstation; TRUE D Syngo MMWP 2009B). We did not correct for partial volume effect based on the resolution of our Siemens FDG-PET scanner (< 5 mm.), considering that the minimum diameter of all the lesions studied were at least threefolds the FHWM (> 1.5 cm). The following parameters of FDG quantitation were obtained (as defined below): SUVmax, SUVmean35, SUL, SUVglu, MTV (metabolic tumor volume), TLG (total lesion glycolysis), and tumor to background index (T/B).

### Microarray processing and statistical methodology

The protocol followed for the obtention of the matched biopsies is the usual one at our institution and has been previously published [17]. Total RNA extraction was done with RNAeasy columns (QIAGEN), and the amount obtained was measured with the Nanodrop spectrophotometer (ND-1000). Quality of the RNA was measured with the Agilent 2100 Bioanalyzer. Microarray processing and the statistical methodology used to build and validate the signature is described in the Additional file 1.

### PET quantification parameters

SUVmean35 was defined based on our previous study (unpublished data) as the SUV mean in a thresholded VOI (3D isocontour at 35% of the maximum pixel value). To calculate T/B index, two identical circular ROIs (region of interest) 50% in size to corresponding VOIs were centered on the area with maximum uptake tumor localization and on the tumor-free neighboring area respectively.

SUL (SUV normalized to lean body mass) was calculated as follows:

$$ \mathrm{SUL}=\mathrm{LBM}\times \raisebox{1ex}{$\mathrm{SUVmean}35$}\!\left/ \!\raisebox{-1ex}{$\mathrm{patient}\  \mathrm{weight}\ \left(\mathrm{kg}\right)$}\right. $$, where

LBM (lean body mass) was calculated according to the formula of Janmahasatian et al. [18]

$$ \mathrm{LBM}\ \mathrm{male}=9270\times \raisebox{1ex}{$\mathrm{patient}\  \mathrm{weight}\ \left(\mathrm{kg}\right)$}\!\left/ \!\raisebox{-1ex}{$\left(6680+216\times \mathrm{BMI}\right)$}\right. $$

$$ \mathrm{LBM}\ \mathrm{female}=9270\times \raisebox{1ex}{$\mathrm{patient}\  \mathrm{weight}\ \left(\mathrm{kg}\right)$}\!\left/ \!\raisebox{-1ex}{$\left(8780+244\times \mathrm{BMI}\right)$}\right., $$

BMI (body mass index): weight/height^2^ (kg/m^2^).

SUV_glu_ (SUV corrected for the blood glucose level) was obtained as follows [19]:

$$ \mathrm{SUVglu}=\raisebox{1ex}{$\left(\mathrm{SUVmean}35\times \mathrm{basal}\  \mathrm{glucose}\right)$}\!\left/ \!\raisebox{-1ex}{$100\ \mathrm{mg}/\mathrm{dl}$}\right. $$

MTV was calculated as tumor volume in centimeter cube contained in the 35% thresholded VOI. TLG was calculated as (SUV mean) × (MTV).

### Selection of a representative FDG uptake value for the predictive signature

Among the different FDG quantification parameters mentioned above, a thorough descriptive statistic was carried out in the 71 patients belonging to the training set. This preliminary analysis showed that SUVmax, SUVmean35, and SUVglu had certain linearity and a data distribution close to a normal distribution as demonstrated by normality tests (Shapiro-Milk and Kolmogorov-Smirnov) and QQ-plots. However, the remaining parameters obtained (SUL, MTV, T/B, and TLG) did not follow a normal distribution and were not linear. We reasoned that it was convenient to choose a representative parameter that followed a normal distribution and that showed certain linearity as some methods making use of principal components are known to have a better fit to this kind of data. Hence, we preferred to use either the SUVmax, SUVmean35, or SUVglu as continuous dependent variable (the response or outcome variable). As expected, there was a very good correlation between SUVmean35, SUVmax, and SUVglu. The Pearson correlation coefficient was highest and most significant between SUVmean35 and SUVmax (*r* = 0.976, *p* < 0.001). Given the concordance between SUVmean35 and SUVmax and to avoid redundancies, SUVmean35 was chosen. Moreover, we selected the SUVmean35 as the dependent (or outcome) variable to be predicted as the parameter representing FDG uptake because of the higher intrinsic uncertainty associated with the calculation of SUVmax and also for the better reproducibility of SUVmean35 in accordance to our experience (unpublished data). Among these three quantification parameters mentioned above, SUVmean35 was also preferred because its calculation has demonstrated greater inter- and intra-observer reproducibility, in agreement with reports recommending the use of the SUV mean in quantifying the biological effects on tumor response [20, 21]. SPSS software version 15.0 for Windows was used for the descriptive statistics.

To get a better fit of the SUVmean35 to a normal distribution, and also to achieve a similar range as the predictors (probes), the SUVmean35 underwent a base 2 logarithmic transformation. The transformed data would be used in the elaboration of the predictive model. It is important to notice that the log-transformed SUVmean35 values from the 71 patients in the training set did not contain any outlier. To improve readability, the term SUV instead of SUVmean35 was used throughout the manuscript.

### Feature selection for building the genomic signature

A key factor to the good performance of predictive models containing a higher number of features (probes in our case) than observations (patient samples) (i.e., *p* ≫ *N*) is the selection of the most relevant features to the response. The algorithm of supervised principal components suggested by Hastie et al. [22] was followed with some modifications. In brief, first, with the predictors standardized, univariate regression coefficients for the outcome (the SUV) for each one of the 22,814 filtered probes was obtained. Second, reduced matrices were formed including only those features that exceeded certain absolute threshold in their regression coefficients, and the first three principal components of these matrices were calculated; then, these principal components were used in a regression model to predict the SUV. The absolute regression coefficient threshold used and the number of principal components were chosen by tenfold cross-validation (CV). The functions *superpc.cv* and *superpc.plotcv* from the *superpc* library (created by one of the authors of [22]) from the R statistical environment were used for this purpose. The third principal component and a regression coefficient threshold of ± 1 were selected. The selected threshold included 909 probes corresponding to 742 genes with gene symbol and 62 probes without it. As these 62 probes without symbol might also have an important contribution to the performance of the signature, they were also kept.

All the 909 probes selected were included to build the predictive model considering each one of them individually as predictor (independent variable). Thus, the statistical models tested were allowed to assign the coefficients (for three of the methods used in this study, see the Additional file 1) or proximity measures (for random forest, the fourth method tested in this study as shown in the Additional file 1) most appropriate for each probe, with the intention of increasing the overall accuracy of the tested models. Also, bias related to any form of summarization of the probes can be avoided in the comparison of the models tested. The full list of the 909 probes along with their regression coefficients is shown in Additional file 2: Table S4. As a measurement of importance of the 909 selected probes, variable importance of projection (VIP) values were calculated using the R library *plsVarSel*.

### Bioinformatics analysis of the selected probes

Hierarchical clustering with the selected 909 probes was performed with the function *hclust* of R, using the Spearman correlation coefficient as distance metric (more precisely 1—correlation coefficient) and complete linkage. A heatmap was generated with the *gplots* library from R.

For a correct interpretation of the results presented in this work, it is worth noticing that no patient in this study had a SUV = 0 (or “negative”). Thus, when we speak below (and throughout this work) about genes (or biological processes) positively or negatively correlated with the SUV, what is implied is that positive refers to a higher and negative to a lower SUV. In other words, all the genes and biological processes studied here have a clear relationship with the SUV.

The DAVID Bioinformatics Resources 6.7 (https://david.ncifcrf.gov/) was used to study the biological processes overrepresented among the signature selected genes. To include additional biological processes less represented in DAVID, another public resource used was the Consensus Pathway Database, release 31 (http://ConsensusPathDB.org) as a complement.

For the study and identification of potential protein-protein interaction (PPI) networks among the genes selected for the predictive signature, all the genes in each subnetwork (positive and negative correlation with the SUV) were first mapped to their respective protein products using the bioinformatics resource STRING 10.0 (https://string-db.org). The threshold used to establish the edges (interactions) among the nodes (proteins) of the PPI networks was 0.7 (“high confidence”). Two subnetworks were studied separately. One built using the genes with positive and another with those with negative correlation with the SUV (according to the sign of their regression coefficients). In addition, hierarchical clustering using a fastgreedy algorithm (done separately in the two subnetworks) was carried out with the libraries *STRINGdb* (http://www.bioconductor.org) as an API to the STRING database and *igraph* from R in order to assign membership in the two subnetworks obtained. The study of network characteristics such as those related with centrality and connectivity was done with the *igraph* library from R in the two subnetworks obtained.

Gene Set Enrichment Analysis (GSEA) was done using the method single-sample GSEA (ssGSEA) as implemented in the library *GSVA* (function gsva, method = “ssgsea”) from R. Default parameters of this method were used as described by Barbie et al. [23]. This method was applied to all the normalized and filtered microarray intensity data after summarization of the 22,814 probes in the training set (retaining only the maximum intensity value for those genes represented by more than one probe and eliminating those probes without a gene symbol). The C2 subset (curated gene sets) from the Molecular Signatures Database (MSigDB) v5.1 maintained by the Broad Institute (http://software.broadinstitute.org/gsea/msigdb/collections.jsp) was used. The scores obtained with ssGSEA for each patient and each signature used in the training set were then pairwise correlated independently with the corresponding transformed SUV values of each patient (Pearson correlation), and the corresponding correlation coefficients and probabilities were obtained for each signature of the C2 subset. To gain further insight into some specific findings obtained with the C2 subset of MSigDB v5.1, other subsets from this database were also used such as the H, C5, and C6 subsets. Using the same ssGSEA methodology described, we also used the 10 genesets containing highly selective and specific genes for 10 different cell populations. These genesets have been validated extensively in thousands of different human solid tumors (> 19,000) to estimate the abundance of immune and non-immune cells and have also been shown to have a good correlation with immunohistochemistry [24].

## Results

### FDG-PET quantification

The characteristics of the patients (demographics and quantification data) and biopsies are shown in Tables 1 and 2. The detailed tumor histologies can be found in Additional file 3: Table S6. No statistically significant differences were found between the training and validation set (U Mann-Whitney *p* > 0.05 for all variables shown). The only difference with the training set was the inclusion of two aggressive locally advanced primary tumors in the validation set: a patient with a pancreatic adenocarcinoma and another with a biliary duct carcinoma.

**Table 1:** Demographics and quantification data in the training and validation sets*;* mean and range values are given

	Training set (*n* = 71)	Validation set (*n* = 13)
Age (years)	58 (28–80)	58 (36–77)
Females/males	40/31	9/4
LBM	52.3 (29.5–81.8)	47.9 (32.8–60.7)
Baseline blood glucose (mg/dl)	100.8 (66–149)	100.1 (78–126)
Injected dose (mCi)	11.5 (9.9–13.4)	11.3 (10,0–12.9)
PET quantification data		
Diameter of the lesion (cm)	6.4 (1.5–18.9)	8.1 (2.1–18.3)
SUVmax	11.8 (3.7–31.3)	12.3 (2.7–21.7)
SUVmed35	6.7 (2.4–16.7)	6.5 (2.0–10.7)
SUL^a^	4.8 (1.5–11)	5.1 (2.3–8.8)
SUVglu	6.7 (2–14.9)	6.5 (2.1–11.5)
MTV (cm^3^)^a^	45.2 (0.7–434)	197.4 (2.1–1009)
TLG^a^	358.7 (2.3–3958.1)	1784 (4.1–9058.1)
T/B	9.5 (1.4–35.8)	10.9 (2.5–26.8)

Abbreviations: *LBM* lean body mass, *SUVmax* maximum standard uptake value, *SUVmed35* thresholded 35% medium standard uptake value, *SUVglu* standard uptake value corrected for plasma glucose levels, *SUL* standard uptake value normalized by lean body mass, *MTV* metabolic tumor value, *TLG* total lesion glycolysis, *T/B* tumor-to-background ratio

^a^Missing data: 3 in the training set and 1 in the validation set

**Table 2:** Tumor histologies and locations of the biopsies obtained for microarray analysis of the patients in the training set

Histology	Total (*n* = 71)	Percentage (%)
Colorectal cancer	9.0	12.7
Breast cancer	8.0	11.3
Soft tissue sarcoma	7.0	9.9
Genitourinary tumor	7.0	9.9
Ovarian cancer	7.0	9.9
Lung cancer	7.0	9.9
Pancreatic cancer	6.0	8.5
Head and neck cancer	5.0	7.0
Esophageal cancer	4.0	5.6
Thyroid cancer	2.0	2.8
Bile duct cancer	2.0	2.8
Carcinoma of unknown primary (CUP)	1.0	1.4
Gastric cancer	1.0	1.4
Lymphoma	1.0	1.4
Melanoma	1.0	1.4
Mesothelioma	1.0	1.4
Merkel cell carcinoma	1.0	1.4
Kidney cancer	1.0	1.4
Locations of biopsies	Total (*n* = 71)	Percentage (%)
Liver	25	35.2
Retroperitoneal	16	22.5
Lymphadenopathy	13	18.3
Head and neck mucosa	3	4.2
Skin	3	4.2
Pleural	3	4.2
Lung	3	4.2
Breast	2	2.8
Mediastinum	2	2.8
Pancreas	1	1.4

### Hierarchical clustering with the probes selected for the elaboration of the signature

**Fig. 1:**
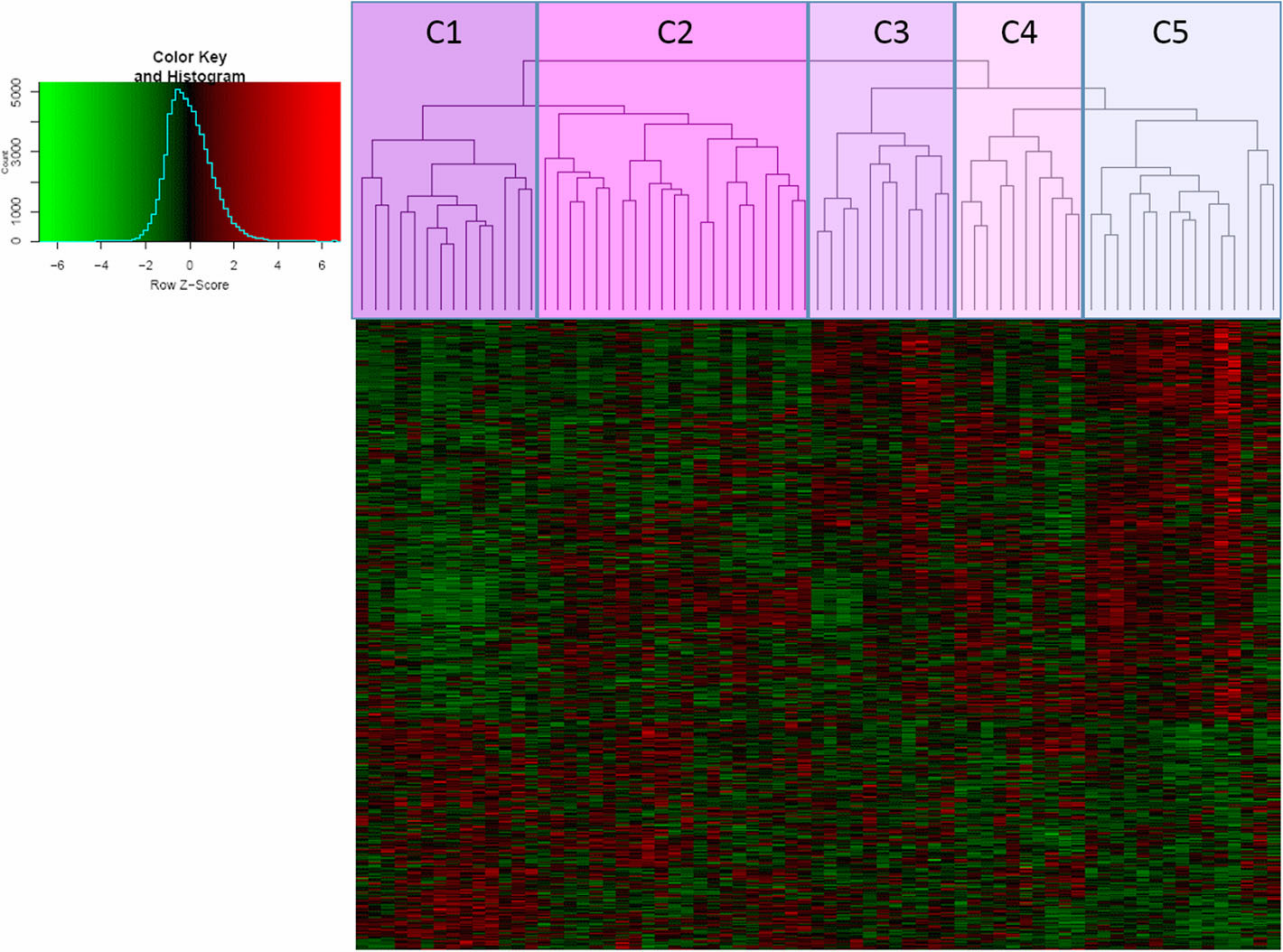
Hierarchical clustering and heatmap of samples in the training set with the 909 probes of the signature. Microarray samples of the 71 patients in the training set are in columns and standardized probes in rows. The five sample clusters obtained are denoted by C1 to C5 in the upper part of the dendrogram

Hierarchical clustering was performed in order to check whether the selected probes (the 909 most strongly correlated with FDG uptake as measured by the SUV) were able to discriminate different groups of patients in the training set according to the SUV values and not to other clinical or pathological data. In Fig. 1, a heatmap is shown with the results of the patient samples hierarchical clustering with the 909 probes (as described in the “Methods” section). Five main clusters could be easily distinguished (C1 to C5 in Fig. 1). Comparing the average SUV values of the patient samples of each of the five clusters (Table 3a), they were significantly different as shown by one-way ANOVA (*p* = 0.001). The SUV averages of the clusters were significantly different on account of significant differences between the higher average SUV of C1 samples versus the average SUV of the remaining clusters (i.e., C1 vs C2, C1 vs C3, C1 vs C4, and C1 vs C5, *p* < 0.05 for all comparisons by the Student *t* test). The C2 vs C5 comparison was found close to significance by *t* test (*p* = 0.076). Therefore, this unsupervised methodology is indeed able to discriminate clusters of patients with statistically significant average SUV values. Furthermore, none of the major tumor types in this series was grouped in a single cluster (for example, breast, colorectal, genitourinary, ovarian, lung cancers, or soft tissue sarcomas). We found that as a group in our training set, lung cancers (7 patients) had an average SUV value significantly higher than most of the other major tumor types. However, lung cancers were evenly distributed in three different clusters. The remainder of the most represented tumor types had average SUV values that were not significantly different among them, and nevertheless, they were distributed in a minimum of two or more clusters. In addition, no statistically significant differences were found between the average SUV values of the metastatic tumors located in the liver (liver biopsies) and the rest of metastatic locations (*t* test *p* = 0.34). Likewise, the samples coming from liver metastases were widely distributed among the five clusters. Overall, these results point to the suitability of these genes as building blocks of a multivariable model to predict the SUV. As a control, hierarchical clustering using the same methodology was also applied to the training set with all the filtered unselected probes to identify five clusters. However, the SUV averages of the clusters identified with all the unselected probes were not significantly different by one-way ANOVA (*p* = 0.357), as shown in Table 3b.

**Table 3:** SUVmean35 (SUV) averages, standard deviations (SD), minimum and maximum values of the samples of each of the five clusters identified using the indicated number of probes in the training set

Cluster	*n*	Average SUV	SD	Minimum SUV	Maximum SUV
a) 909 selected probes					
C1	14	9.28	2.88	5.27	16.69
C2	21	6.89	3.08	2.52	13.63
C3	11	6.46	2.86	3.78	12.08
C4	10	5.28	1.69	2.62	8.38
C5	15	5.21	2.08	2.35	9.16
One way ANOVA, *p* = 0.001					
b) 22,814 unselected probes					
C1	13	7.01	3.49	2.35	13.63
C2	11	6.38	2.74	2.61	12.08
C3	15	6.09	2.49	2.52	12.61
C4	19	7.77	3.51	2.62	16.69
C5	13	5.86	1.97	3.41	9.16
One way ANOVA, *p* = 0.357					

### Biological processes related to the selected genes

Tables 4 and 5 show the top 20 most significantly overrepresented biological processes related to the genes with positive and negative correlation with the SUV. Among the biological processes with positive correlation with the SUV, it was interesting to note the RNA processing, ncRNA processing, RNA splicing, ribosome biogenesis, and protein aminoacid N-linked glycosylation via asparagine. All these processes were related to the preliminary and required steps conducing to the synthesis and processing of proteins. Cellular growth rate is directly proportional to the number of new ribosomes formed in a cell [25]. Among the biological processes with negative correlation with the SUV, cell adhesion, actin cytoskeleton organization and its regulation, regulation of glycogen biosynthetic process, and ruffle organization were noticeable.

**Table 4:** Biological processes overrepresented in the genes with positive correlation with the SUV (from DAVID Bioinformatics Resources 6.7)

Term	Percent	*p* value	Benjamini
GO:0006396~RNA processing	10.3	1.87E−08	0.000
GO:0022613~ribonucleoprotein complex biogenesis	4.8	1.09E−05	0.007
GO:0034470~ncRNA processing	4.4	8.05E−05	0.036
GO:0034660~ncRNA metabolic process	4.8	1.21E−04	0.040
GO:0046148~pigment biosynthetic process	2.2	2.23E−04	0.059
GO:0008380~RNA splicing	5.1	2.32E−04	0.051
GO:0016071~mRNA metabolic process	5.9	2.91E−04	0.055
GO:0018279~protein amino acid N-linked glycosylation via asparagine	1.5	3.24E−04	0.054
GO:0018196~peptidyl-asparagine modification	1.5	3.24E−04	0.054
GO:0042254~ribosome biogenesis	3.3	3.70E−04	0.055
GO:0042440~pigment metabolic process	2.2	4.42E−04	0.058
GO:0006397~mRNA processing	5.1	7.42E−04	0.088
GO:0009101~glycoprotein biosynthetic process	3.3	0.002	0.203
GO:0070085~glycosylation	2.9	0.002	0.228
GO:0006486~protein amino acid glycosylation	2.9	0.002	0.228
GO:0043413~biopolymer glycosylation	2.9	0.002	0.228
GO:0065003~macromolecular complex assembly	7.4	0.003	0.273
GO:0006487~protein amino acid N-linked glycosylation	1.8	0.004	0.277
GO:0008033~tRNA processing	2.2	0.005	0.329
GO:0000375~RNA splicing, via transesterification reactions	2.9	0.007	0.409

**Table 5:** Biological processes overrepresented in the genes with negative correlation with the SUV (from DAVID Bioinformatics Resources 6.7)

Term	Percent	*p* value	Benjamini
GO:0007160~cell-matrix adhesion	2.9	9.20E−07	0.002
GO:0031589~cell-substrate adhesion	2.9	2.61E−06	0.002
GO:0030029~actin filament-based process	4.2	1.27E−05	0.008
GO:0007155~cell adhesion	8.0	1.46E−05	0.007
GO:0022610~biological adhesion	8.0	1.47E−05	0.005
GO:0007015~actin filament organization	2.2	4.01E−05	0.012
GO:0030036~actin cytoskeleton organization	3.8	7.40E−05	0.019
GO:0051493~regulation of cytoskeleton organization	2.9	7.46E−05	0.017
GO:0051017~actin filament bundle formation	1.3	9.63E−05	0.019
GO:0005979~regulation of glycogen biosynthetic process	1.1	2.34E−04	0.042
GO:0032885~regulation of polysaccharide biosynthetic process	1.1	2.34E−04	0.042
GO:0010962~regulation of glucan biosynthetic process	1.1	2.34E−04	0.042
GO:0048771~tissue remodeling	1.8	2.91E−04	0.047
GO:0032881~regulation of polysaccharide metabolic process	1.1	3.14E−04	0.046
GO:0031529~ruffle organization	0.9	9.29E−04	0.121
GO:0043244~regulation of protein complex disassembly	1.6	0.001	0.132
GO:0043255~regulation of carbohydrate biosynthetic process	1.1	0.001	0.138
GO:0008015~blood circulation	2.9	0.001	0.138
GO:0003013~circulatory system process	2.9	0.001	0.138
GO:0035150~regulation of tube size	1.6	0.001	0.134

We also checked the Consensus Pathway database with the same genes (see Additional file 4: Table S1). Other processes of potential interest not identified by DAVID were noted (all with *q* ≤ 0.1). Regarding the genes with positive correlation with the SUV, this database unveils biological processes such as scavenging by class A receptors, DNA replication, and its regulation. Other relevant processes identified are those related to the immune system: PD1 signaling, antigen processing and presentation, CD4 T cell receptor signaling, downstream TCR signaling, phosphorylation of CD3, and TCR zeta chains among others. Previous reports have shown that a high glucose uptake is required for T cell activation [26].

Although less statistically significant than the aforementioned biological processes, those related to the energetic metabolism of carbohydrates were apparent: glycolysis, pentose phosphate cycle, and insulin-mediated glucose transport. In common with DAVID, protein processing and N-linked glycosylation were also apparent. As far as the genes with negative correlation with the SUV were concerned, a deeper biological insight could be obtained from the Consensus Pathway database (all with *q* < 0.05). In common with DAVID, the regulation of the actin cytoskeleton scores high. However, a relevant contribution to this regulation can be envisaged in the identified biological pathways related to the small GTPases RHO, RAC1, and CDC42 as they are known potential controllers of dynamic processes affecting the cytoskeleton such as the formation of stress fibers (RHO), lamellipodia (RAC1), and filopodia (CDC42) as well as membrane ruffling (RAC1). E-cadherin signaling, integrin, integrin-linked kinase signaling, and focal adhesions also seem relevant to cell adhesion processes. Muscle and smooth muscle contraction processes cannot be overlooked as they may correlate with some of the cytoskeleton changes and with motility. Eukaryotic translation termination is also worth mentioning. Another potentially relevant group of biological processes are those related to common downstream signaling by different growth factors particularly through RAS and the RAF/MAPK cascade and last but not the least, signaling by the VEGFA-VEGFR2 pathway. Several of the processes mentioned may in fact occur in the tumor microenvironment, like those related to angiogenesis.

### Identification of protein-protein interaction (PPI) subnetworks among the selected genes

To identify in each subnetwork (positive and negative correlation with the SUV) modules of relevant functional (and/or physical) interactions assigning membership to each of the interacting proteins, we applied a fastgreedy clustering algorithm, disregarding the proteins with no interactions. After applying the procedure, we selected those clusters containing at least three proteins in each of the two subnetworks. Ten clusters of these characteristics were isolated in the subnetwork of proteins with positive correlation with the SUV and 16 clusters in the subnetwork of those with negative correlation. All the isolated clusters were highly significant in their PPI enrichment value, as defined by the authors of [27] (the cluster range of PPI enrichment *p* values was from 0.00277 to < 5 × 10^−16^). This means that there were more interactions among the proteins in each of the clusters considered than would be expected by chance alone in a random set of proteins of similar size extracted from the genome, suggesting a functional cooperation among them. Some potentially relevant clusters are shown in Additional file 5: Figure S1. Among the clusters obtained from the subnetwork of genes with positive correlation with the SUV, clusters 1, 4, 6, and 8 are shown. Cluster 1 is shown containing proteins related to the folding and processing of proteins (HSP90B1, DNAJA1, CALR), transport of proteins (CLPB), proteins with special relevance in hypoxia-like HYOU1, the gene that encodes ORP150, that is overexpressed in many tumors and it is tightly correlated with invasion and tumor progression [28–30] and proteins involved in the glycosylation of proteins (RPN1, STT3A, MAGT1, and DDOST). Overall, this cluster has to do with different stages of protein processing. Cluster 6 shows ribonucleoproteins with a role in the different stages of preparation of the pre-mRNA, like the assembly (NHP2L1 and HNRNPL), elongation (EFTUD2), and splicing (SUGP1, TXNL4A). Cluster 4 contains mainly proteins related to the biogenesis of ribosomes (PES1, RRP1, RRP1B, BMS1, EBNA1BP2). And cluster 8 shows glycolysis enzymes (GPI, PFKP, and HK3). Among the clusters obtained from the subnetwork of genes with negative correlation with the SUV in Additional file 5: Figure S1, cluster 11 is also shown. It contains proteins associated with cellular adhesion and the reorganization of the cytoskeleton (MAPK3, RHOC, RHOA, RAC1, MYL, CTNNB1, and CTNNA1 among others) and also angiogenesis (TEK, FGFR1, HGF). Some of these processes occur in the tumor microenvironment. Cluster 16 contains large ribosomal proteins (RPL9, RPL12, RPL14, RPL19, RPL21, RPL24) and translation termination (ETF1). Of particular interest is cluster 19 containing proteins related to autophagy (ATG5, ATG7, SQSTM1), a homeostatic process that can function in both tumor and stromal cells under conditions of low input of nutrients; in tumors, it has also been related to therapeutic resistance to some antineoplastic agents such as tyrosine kinase inhibitors [31]. It is also interesting to underline that autophagy is not a hit in any other method used in this study.

### Gene Set Enrichment Analysis (GSEA)

In order to characterize further the biological and signaling pathways related to the uptake of FDG as measured by the SUV, we performed single sample GSEA (ssGSEA) in our whole filtered training dataset as explained in the “Methods” section, with the C2 subset of the Molecular Signatures Database (MSigDB) v5.1. The significant results obtained (*p* < 0.05) are reported in Additional file 6: Table S2. This method (ssGSEA) can identify coordinated changes of genes belonging to a gene set in a more sensitive way than other over-representation methods (more centered on individual genes) like the ones mentioned above, that could miss some signaling or biological pathways. That is why we also used this method to complement the methods commented above. Nonetheless, the results were consistent with those of DAVID, Consensus Pathways, and STRING PPIs databases. Just to mention a few, in common with these databases pathways involved in motility (KEGG_vascular_smooth_muscle_contraction, *r* = − 0.3827), reorganization of the cytoskeleton (KEGG_regulation_of_actin_cytoskeleton, *r* = − 0.269), cell adhesion (st_integrin_signaling_pathway, *r* = − 0.3382), and angiogenesis (pid_lymph_angiogenesis_pathway, *r* = − 0.25) were identified as negatively correlated with the SUV in a statistically significant manner. Another significant gene set identified as positively correlated with the SUV that is worth mentioning is the reactome_facilitative_na_independent_glucose_transporters (*r* = 0.2496). ssGSEA indeed identified statistically significant relevant signaling pathways missed by the other methods used above. Particularly relevant, the activation of c-MYC is positively correlated with the SUV in some of the genesets of the C2 subset of MSigDB studied (dang_regulated_by_myc_up, *r* = 0.2839, coller_myc_targets_down, *r* = − 0.274, and dang_myc_targets_up, *r* = 0.2436). To strengthen this relationship, we also used ssGSEA with the C6 (oncogenic signatures) and the H (hallmark genesets) subsets of MSigDB v5.1. We found that the single geneset related to upregulation of MYC of the C6 subset, MYC_UP.V1_UP, was significantly associated (*p* = 0.031) with the SUV (*r* = 0.25) and that HALLMARK_MYC_TARGETS_V2 was also borderline significantly associated with the SUV (*r* = 0.23, *p* = 0.052). The hallmark subset contains just an additional HALLMARK_MYC_TARGETS_V1 geneset which, although not significant, it was also positively correlated with the SUV (*r* = 0.12). Overall, this in-silico results on MYC targets appear to suggest that MYC targets are positively associated with higher SUV levels, although experimental confirmation would be required for specific tumor types. MYC upregulation is related to the metabolism reprogramming in cancer cells, influencing a variety of aspects [32]. Overall, the biological processes mentioned above seem to fit the hallmark of several of the stress phenotypes of cancer [33].

As mentioned above in the section “Biological processes related to the selected genes,” several biological processes related to the immune system were found significantly associated with higher SUV values in the Consensus Pathway database. We explored whether these biological processes were related to a change in the population abundance of specific immune cells in association with the SUV. For this purpose, we also used likewise ssGSEA with 10 genesets reported by [24]. A statistically significant association of the abundance of cytotoxic lymphocytes with higher SUV values was found (*r* = 0.29, *p* value = 0.013). Cytotoxic lymphocytes comprise T cells and NK (natural killer) cells. This correlation appears to suggest that there is a trend towards a recruitment of these cytotoxic lymphocytes in tumors with a higher uptake of FDG. The other finding of interest was a borderline statistically significant association of the abundance of endothelial cells in tumors with lower SUV values (*r* = − 0.23, *p* value = 0.055). This result is in agreement with the findings reported above, linking angiogenesis with lower SUV values.

### Building a signature to predict the SUV

After the selection of features and the study of their biological meaning, we fitted and compared four different models: partial least squares (PLS), principal components regression (PCR), support vector machine (SVM), and random forest (RF) to the training set (*n* = 71) by tenfold cross-validation (CV) repeated five times (tenfold CV × 5), selecting the best parameters that for each model minimize the RMSE and maximize *R*^2^. Fifty resamples per model were generated with their respective RMSE and *R*^2^ values as metrics of performance. A summary of the results obtained is shown in Table 6. For the pairwise comparison between models of the RMSE and *R*^2^ values, a *t* test or a Wilcoxon test (both with Bonferroni correction) were used respectively, and the results are also shown in Table 7. PLS and PCR were the models with the best performance (lower RMSE and higher *R*^2^), and there were no statistically significant differences between them. For the final selection of the model, we also took into account the number of components needed to achieve the lowest RMSE between the two best performing models. Hence, PLS requiring three components (PLS-3) was preferred over PCR (which required 18) on the basis of the statistical principle of parsimony.

**Table 6:** Summary statistics of metrics RMSE and *R*^2^ in the four models tested in the training set (50 resamples)

Models	*R* ^2^	SD	CI (95%)	RMSE	SD	CI (95%)
PLS	0.567	± 0.234	(0.035–0.886)	0.443	± 0.119	(0.257–0.662)
PCR	0.576	± 0.228	(0.072–0.891)	0.431	± 0.111	(0.228–0.611)
RF	0.461	± 0.273	(0.043–0.873)	0.526	± 0.120	(0.337–0.719)
SVM	0.501	± 0.260	(0.037–0.895)	0.476	± 0.128	(0.274–0.659)

**Table 7:** Results of pairwise comparisons between methods

Models	RMSE (*t* test adjusted *p* values)	*R* ^2^ (Wilcoxon test adjusted *p* values)
PLS vs PCR	0.08936	0.3108
PLS vs RF	5.3E−07	0.01667
PLS vs SVM	0.0002238	0.000962
PCR vs RF	2.087E−11	5.925E−5
PCR vs SVM	2.198E−07	0.001221
RF vs SVM	5.05E−06	0.2839

Additional file 5: Figure S2 shows the goodness of fit of the predictions of the SUV made by PLS-3 in the training set, as this fit would be used to validate the model in our independent validation set (*n* = 13). As a more realistic estimate of the goodness of fit in an independent validation set, we performed a tenfold CV in the training set (Additional file 5: Figure S2c).

### Measurement of performance of the PLS-3 signature in an independent validation set

The characteristics of the patients of the validation set along with the actual measured and predicted SUV values (by PLS-3) are shown in Additional file 7: Table S3. As mentioned in the “Patients and Methods” section, one of the patients in this validation set can be considered an influential observation as her measured SUV was below the range of SUVs measured in the training set (patient 9 in Additional file 7: Table S3). The measured SUV in this patient was 1.96, and the prediction made by PLS-3 was actually suboptimal: 6.43 (i.e., 3.28-fold higher than the measured value). Taking into account this patient, the RMSE obtained in the validation set (*n* = 13) was 0.645 which nevertheless is within the 95% confidence interval of the tenfold CV × 5 used to select the best model (see Table 6). Excluding this patient, the RMSE of the validation set (*n* = 12) would be 0.454 and quite similar to the mean RMSE value estimated by tenfold CV × 5 (0.443 as seen in Table 6). Therefore, the estimates of performance of PLS-3 obtained by tenfold CV × 5 were accurate in predicting the performance of the signature in our independent test set. The inclusion of the influential observation worsens the performance of the signature. Using as a benchmark the RMSE values of the validation set with (*n* = 13) and without (*n* = 12) the influential observation using the 909 probes of the original signature, we next tested the performance and stability of the PLS-3 signature by both reducing and increasing the number of probes.

**Fig. 2:**
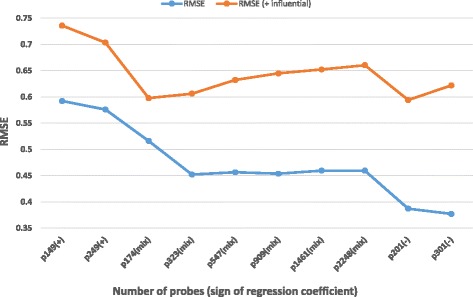
RMSE values in the validation set (with or without influential observation) of PLS-3 signatures with different number of probes ((+) only probes with positive regression coefficients, (mix) probes with both positive and negative regression coefficients, and(−) only probes with negative regression coefficients)

In addition to the 149 and 249 probe signatures (only with positive regression coefficients) and the 201 and 301 probe signatures (only with negative regression coefficients) commented in Additional file 1, we also tested the signatures containing probes with an absolute regression coefficient varying by 0.1 intervals (i.e., including probes with both positive and negative regression coefficients. In Fig. 2, a graph showing the RMSE results of the different PLS-3 signatures tested in the validation set with (*n* = 13) and without (*n* = 12) the influential observation is shown. All signatures were first trained by PLS-3 in the training set, and then, the model generated was tested in the validation set.

It was noticeable that all tested signatures perform worse in the full validation set containing the influential observation (*n* = 13) than in the same validation set without it (*n* = 12). Therefore, it is not advisable to include such observations in future testing of the predictive signature. Further, a trend towards a degradation of performance is apparent in the tested signatures with an increasing number of probes in the full validation set. However, a clear picture emerges by seeing the performance RMSE results of Fig. 2 in the validation set when the influential observation is omitted (*n* = 12). The signatures containing exclusively probes with positive regression coefficients (the 149 and 249 probe signatures) perform worse than all the remainder (RMSE = 0.59 and 0.57 respectively). The signatures containing exclusively probes with negative regression coefficients (the 201 and 301 probes signatures) perform the best in the validation dataset (RMSE = 0.39 and 0.38 respectively) and perform better than the results obtained with signatures containing a mixture of probes with both positive and negative regression coefficients. Moreover, the performance of the original signature (909 probes) is not degraded by either increasing (the 0.8 and 0.9 threshold signatures) or reducing (the 1.1 and 1.2 threshold signatures) the number of probes. The RMSE for the original PLS-3 signature with 909 probes (1.0 threshold) is approximately the same (between 0.45 and 0.46) as the 0.8 (2248 probes), 0.9 (1461 probes), 1.1 (547 probes), and 1.2 (323 probes) threshold signatures. Only the 1.3 threshold signature (174 probes) has a worse performance (RMSE = 0.52).

### Evaluation of the importance of the probes comprising the PLS-3 signature by using the variable importance of projection (VIP)

As a measurement of importance of the PLS-3 probes, we estimated the VIP values of the third component of our PLS model, which are useful to evaluate the relative contribution of each probe to the model. In Additional file 8: Table S5, the 320 probes of the signature with VIP values ≥ 1 are shown. This threshold is frequently chosen to select the most relevant features that contribute to PLS models. The higher the VIP value for a specific predictor (in our case probe), the more relevant it is for the PLS model. In Additional file 8: Table S5, 14 probes not matched to a symbol have VIP values ≥ 1, and 2 of them are among the top 10 probes with the highest VIP values. It is interesting to note that the proportion of probes not matched to a symbol in the whole signature is not significantly different from this proportion in the selected probes with VIP values ≥ 1 (*p* = 0.137, by Fisher exact test). These results show that the selection of probes according to VIP values is not enriched in probes matched to a symbol as compared with the whole 909 probe signature, and therefore that unmatched probes seem to also have a similar relevant contribution to the PLS-3 model as matched ones with the same characteristics of VIP values.

Just to mention a few putative relevant genes among the probes matched to a symbol, TEK (also called TIE2) scores high (VIP = 2.06). TEK is a kinase that is expressed in endothelial cells and is involved in angiogenesis. Other selected probes are also related to signaling pathways involving angiogenesis such as NRP2, HGF, and FGFR1. EDNRA and EDNRB have also been found expressed in endothelial cells in different human tumors and a role in angiogenesis and cancer metastasis has been reported [34, 35]. It is also interesting to find RHOJ that is known to be enriched in tumor endothelial cells and to be involved in their motility and in tumor progression, and it is also considered a selective antiangiogenic target [36]. Several genes related to autophagy (ATG7, SQSTM1, ULK2), the glycolytic enzyme HK3 and cytoskeleton organization like RAC1 among others were also selected.

## Discussion

One of the most relevant contributions of this study is the generation and validation of a novel genomic signature using methods of regression not previously reported for the prediction of FDG uptake in diverse metastatic tumors. We reasoned that by predicting the intensity of FDG uptake, we could derive a better understanding of the glucose requirements of the biological processes operating in different metastatic tumors. We found that the best performing model in our dataset was PLS-3. We acknowledge that the small size of our independent validation set (*n* = 13) might have been a limitation of our study. Notwithstanding the PLS-3 signature has also been thoroughly validated by tenfold CV × 5 in a balanced training set (*n* = 71). Tenfold CV × 5 was accurate in estimating the performance of the signature in the independent validation set. The PLS-3 signature has a high stability, as shown by similar performance in the validation set using a wide number of probes (from 323 to 2248, see Fig. 2 without the influential observation). And also, the possibility of using a reduced version of the signature with a lower number of probes seems to be feasible.

It was also of interest to note that PLS-3 explained 89% of the SUV variance. It must be taken into consideration that the SUV has some known and definite sources of irreducible error. Among these, methodological aspects related to the preparation of the patient and his/her own physiology, and also those related to the quantification and processing of the PET study which can account for up to 20% of the variation obtained when acquiring the SUV value [1]. The interobserver reproducibility of the SUV (≈ 10%) is also a known issue [37].

It was of interest to note that biological processes previously described as relevant to FDG uptake, like glycolysis and glucose transport, were identified in the bioinformatics analysis carried out in the present work. These findings lend credence to the methodological approach followed in this study. Moreover, another relevant contribution of this study is the identification in a variety of metastatic tumors of multiple common biological processes beyond glycolysis correlated with different SUV values (higher or lower) through a bioinformatics analysis of the signature genes. The knowledge acquired in this study could be of use to design specific targeted therapies based on SUV values or on its variation in response to antineoplastic treatments.

As could be expected, the different bioinformatics methods used (DAVID, Consensus Pathway and STRING databases, and ssGSEA) in this study show some degree of overlapping in the biological processes identified, but they are complementary as each one also finds some relevant unique biological processes not identified by the others. This makes worthwhile the combined use of all of them for a thorough view of the biological landscape of FDG uptake. There seems to be among the biological processes correlating with lower SUV values a preponderance of biological processes related with the tumor microenvironment and its interaction with tumor cells (such as cell adhesion, cell motility, cytoskeleton reorganization, autophagy, and lymphangiogenesis). Of special interest are the processes related to angiogenesis and specifically neolymphangiogenesis. Recently, it has been shown that in primary melanomas, distant lymph nodes and organs may increase their lymphatic vessel density as a pre-metastatic niche to favor and promote distant metastases through the tumor secretion of midkine (encoded by *MDK*) [38]. Consistent with these data, *MDK* is weakly correlated with lower SUV values in our training dataset, and it is known that this heparin-binding factor is also secreted by several cancer types (e.g., pancreatic carcinoma) [38]. Hence, it could be inferred that neolymphangiogenesis occurs not only at the pre-metastatic stage, but also at the initial stages of the metastatic process when apparently a lower uptake of glucose is required and/or available.

Among the biological processes correlated with higher SUV values, the predominant biological processes have to do mainly with the tumor compartment (like ribosome biogenesis, DNA replication, and RNA processing and splicing) and also with the immune system. These data suggest that as metastatic tumors evolve through the acquisition of new mutations towards more advanced stages of the metastatic process, which seem to require a higher glucose uptake, they also generate neoantigens capable of inducing effective T cell responses which may compete with the tumor for a higher glucose uptake.

To the best of our knowledge, only a few previous reports have characterized signatures predicting FDG uptake from microarray data [14, 15, 39]. In common with our study, Palaskas et al. [39] used samples (clinical and cell lines) from different histological origins finding in all, enrichment of glucose metabolic pathways (e.g., glycolysis/gluconeogenesis, pentose phosphate pathway) in samples with “high” versus “low” FDG uptake. They also elaborated a classifier by weighted gene voting [40] using as training set 11 primary breast cancer patients (5 with “high” and 6 with “low” FDG uptake) and tested it in 7 breast cancer cell lines. Although with different methodology, we also found among our samples from a diverse variety of metastatic tumors, most of the same top enriched metabolic pathways, including upregulation of MYC.

The other two studies on signatures predicting FDG uptake in non-small cell lung cancer (NSCLC), which included regression studies, were from the same group [14, 15]. In [14], models are built to predict radiologic image features (114 plus PET SUVmax) in terms of 56 metagenes (defined as the first principal component of each one of the corresponding 56 most homogeneous clusters of coexpressed genes) derived from matched microarray and CT imaging data. Using the same methodology and developing further this model to predict specifically 14 FDG uptake features, Nair et al. [15] trained a linear regression model in their study cohort of patients (*n* = 25) with NSCLC with the metagenes most significantly associated with the FDG uptake features. The range of accuracies reported in their study cohort (as defined in (14)) was from 0.725 to 0.875. Using this metric, the higher accuracy values the better performance of the model tested (maximum around 1). When using the same metric (the accuracy) in our dataset (instead of RMSE), we obtained values of 0.95 in our training set and 0.78 in our validation set. These values compare favorably with those reported by Nair et al. [15].

Only one of the signatures reported in [15] was found statistically significant in a multivariable Cox regression model in their external cohort (*n* = 63), but not in their validation cohort (*n* = 84). This signature predicted the SUVmax by means of a linear regression of 15 metagenes, comprising 508 genes.

We wondered whether the genes selected for the elaboration of our predictive signature were able, in an unsupervised manner, to separate patients with different prognosis. For this purpose, we performed hierarchical clustering in a previously published large microarray series of primary breast cancer patients (*n* = 850) with availability of distant metastases-free survival data (DMFS) [41]. The clustering technique was the same used in the training set of this study. Statistically significant differences in DMFS were found among the clusters identified with the signature probes by logrank test (*p* = 0.001). However, in agreement with Nair et al. [15] in NSCLC, we also found that a multivariable Cox regression analysis adjusted for known prognostic factors in breast cancer failed to show an independent prognostic value in this primary breast cancer series (data not shown).

## Conclusions

In summary, we obtained and validated PLS-3 predicting accurately FDG uptake intensity in different metastatic tumors. The PLS-3 genes allowed us to understand better the biological processes underlying the different requirements of FDG uptake in such tumors. FDG-PET might help in the design of specific targeted therapies directed to counteract the malignant biological processes more likely activated in a tumor as inferred from the SUV and also from its variations in response to antineoplastic treatments.

## Additional files

Additional file 1: Supplementary Methods. (DOCX 29 kb)

Additional file 2: Table S4. Complete list of the 909 probes selected for the generation of the multivariable model along with their correspondent regression coefficient. (DOCX 110 kb)

Additional file 3: Table S6. Detailed tumor histologies of the patients in the training and validation datasets. (DOCX 17 kb)

Additional file 4: Table S1. Biological processes related to the signature genes (*Consensus Pathway Database, release 31* (http://ConsensusPathDB.org)). (DOCX 33 kb)

Additional file 5: Figure S1. Selected clusters identified in the Protein Protein Interaction (PPI) subnetworks obtained from the signature genes in the STRING 10.0 PPI database. Figure S2. Goodness of fit of PLS-3 in the training set. a) Goodnes of fit including Pearson correlation of measured vs predicted SUV values b) Residuals of third component. No pattern is apparent in the residuals distribution c) Estimated goodness of fit after 10-fold CV. (ZIP 242 kb)

Additional file 6: Table S2. Correlation coefficient (CC) on SUV of ssGSEA scores with the C2 subset from the MSigDB v5.1 in the training dataset (*p* < 0.05) (DOCX 31 kb)

Additional file 7: Table S3. Characteristics of the patients in the validation set along with their measured and predicted (SUVPLS) SUV values. (DOCX 13 kb)

Additional file 8: Table S5. List of PLS-3 probes with VIP values equal or greater than 1 along with their regression coefficients. (XLSX 34 kb)
