# Supplementary material for: A novel genomic signature predicting FDG uptake in diverse metastatic tumors
Source: EJNMMI Res. 2018 Jan 18;8:4. doi: 10.1186/s13550-017-0355-3 (PMC5773462; doi:10.1186/s13550-017-0355-3)
Supplement: Supplementary file 1 — Supplementary Methods. (DOCX 29 kb) [file 13550_2017_355_MOESM1_ESM.docx]

**Supplementary Methods**

*Gene expression microarrays*

A two-color protocol was used as previously reported (see reference 17 in main **Patients and Methods** section). In the present study, the information provided by the control RNA labeled with Cy3 was not used (except for its contribution to dye bias correction). Only the information provided by the Cy5 labeled tumor RNAs was reanalyzed after dye bias correction. Dye bias correction consisted of both a global approach to dye normalization (using a linear normalization) and a local approach (using lowess normalization), assuming that dye bias may (local) or may not (global) be intensity dependent using rank consistent probes. This procedure was used as implemented in the Agilent Feature Extraction software version 9.5.1 with default settings.

In addition, further preprocessing of the microarrays consisted of: 1) background correction using the normexp method (offset= 50), 2) inter-array quantile normalization of the Cy5 expression data after dye bias correction, 3) base 2 logarithmic transformation and 4) obtention of the median intensity values of the 264 non-control probes that are replicated 9 times in each microarray. In this way, all microarrays were reduced to 41000 unique probes. All the described preprocessing steps were performed using the R statistical environment (<https://cran.r-project.org>) version 3.2.5 and the *limma* library from the Bioconductor project (<http://www.bioconductor.org>).

Given the fact that the preprocessed microarrays contain probes that either are not expressed or show little variation among the different samples of this study, a low variance filtering step was added to eliminate them as they will not have any contribution to the model building step. For this purpose those probes with an interquantile range (IQR) < 0.5 were excluded from further analysis, using the *genefilter* library from the Bioconductor project. The filtering brought down the number of probes from 41000 to 22814 per microarray.

*Selection of the multivariable model to predict the SUV*

After selecting the features (probes) that would be used to build the signature predicting the SUV (as a continuous dependent variable), we decided to compare four regression methods among which we would select the one with the best performance. The 4 regression methods compared were: Partial least squares (PLS), principal components regression (PCR), support vector machines (SVM) and random forest (RF). PLS and PCR are linear methods that were chosen on the basis of the considerations made regarding the selection of the response variable (the SUV). As the log2 transformed SUV has a good fit to a normal distribution and the predictors (probe expression values) were also log2 transformed (making a larger number of probes have a good fit to a normal distribution), it was expected that methods making use of principal components, like PLS and PCR, might be a good choice. SVM and RF are non-linear methods of regression that are considered state of the art in predictive modelling. Both work quite well in diverse situations, so we also decided to include them in the comparative study.

The PLS regression method with the orthogonal scores algorithm was used as implemented in the library *pls* of R (1). This is a supervised method that makes use not only of the variance found in the predictors but also of that in the response (SUV), finding directions (components) in the data that help to understand simultaneously both variances. To fit the model, the only parameter required is the number of components used. The predictors were standardized prior to fitting the model (i.e. probewise the mean intensity value was subtracted and the result divided by the standard deviation of each probe).

The PCR regression method used here is also implemented in the library *pls* of R (1). It makes use of the multidimensional reduction technique of principal components, obtained by singular value decomposition. This method is not supervised as it only takes into account the directions of maximal variability found in the predictors which do not necessarily correlate with the response. For this reason a higher number of components is usually required to predict a response as compared with PLS. The number of principal components is the parameter needed to fit this model. The predictors were standardized prior to fitting the model.

The SVM regression used in this study (the epsilon support vector regression) is implemented in the library *kernlab* of R (2). The selected kernel was one of the most commonly used for all purpose predictions: the kernel of basic radial function. This regression method tries to minimize the effect of large absolute residuals in the regression by using a function that establishes a threshold according to which absolute residual values within the threshold do not contribute to fitting the regression model, while those absolute residual values beyond the threshold do contribute to the fitting process. The two parameters needed are σ (for the basic radial function kernel) and the cost parameter that is chosen by the user as a penalty for large residuals. The predictors were standardized prior to fitting the model.

The RF algorithm (3) used here is implemented in the library *randomForest* of R. RF is an ensemble of decisory trees. Different trained models predict better than each model considered individually. The algorithm consists of the generation of bootstrap samples from the original data, which are used to train several decisory trees models. Partitions are made, and at each one a specific number of predictors are randomly selected among the available ones, selecting the best to partition the data. This process is repeated until the tree is completed. For this study the number of trees used was fixed to 1000. The other parameter needed to fit the model was the specific number of predictors randomly selected at each partition.

Both, to select the optimal parameters of each model and to compare the performance of the 4 regression models tested, we used as metrics (and in the following order of precedence): a) the minimization of the Root Mean Square Error (RMSE) and b) the maximization of R2, by means of using a 10-fold cross-validation (CV) repeated 5 times. RMSE was defined in this study as:

$$\sqrt{\frac{1}{n}\sum_{i=1}^{n} \left( y_{i}-\hat{y}_{i} \right)^{2}}$$

Where “n” is the total number of patient samples; “*y_i_*” is the measured transformed SUV in sample “i”, and “$\hat{y}$_i_” is the predicted transformed SUV in sample “i”.

R2 was defined here as the square of the Pearson correlation coefficient between the predicted and the measured values of SUV. We gave more importance to RMSE because it is a true measurement of performance whereas R2 is not. R2 has to do with the correlation between the measured and predicted outcomes and reflects the percent of variance explained by the model.

From the 71 patients of the training set, fifty resamples of each of the models tested were taken by 10-fold CV repeated 5 times. In brief, for all models tested, the exact same starting seed of pseudorandom number generation (function *set.seed* of R) was used so as to generate, in a reproducible manner, the same partitions of the training data for each of the 4 models. Each model was trained in 9 out of 10 roughly equal partitions generated after a 10-fold CV and tested in the spare one. In turn each of the 9 remaining generated partitions was used once to test each model trained in the 9 remaining partitions. The whole process was repeated sequentially 5 times, ending with 50 different partitions of the data. For each of the 50 resamples a value of RMSE and a value of R2 was obtained, and in this way the metrics of the models trained in the same partitions of the training data would be comparable. In order to achieve this goal, the 4 models were trained by 10-fold CV repeated 5 times using the function *train* from the *caret* package of R (4) as a convenient wrapper for each one of the libraries of the models tested (i.e. *pls*, *kernlab* and *randomForest*). The 50 values of RMSE and R2 obtained in the CV process for each model were available for pairwise comparison. To compare the differences of RMSE values between the models a paired t-test with Bonferroni correction was applied using the function *diff.resamples* from the *caret* library (these RMSE data did not depart significantly from a normal distribution, as shown by normality tests). For the differences of R2 values a paired Wilcoxon test with Bonferroni correction was applied using likewise the function *diff.resamples* (the R2 data departed significantly from a normal distribution, as shown by normality tests).

From these all against all pairwise comparisons of the 4 models tested, the model with the best performance will be chosen for further validation. After this process of internal validation (10-fold CV repeated 5 times), the chosen model would be tested in our (external) validation set (n=13), as an independent test set by calculating the RMSE obtained after applying the signature.

The performance of the original signature (with 909 probes) in the validation set will be used as a benchmark to test further the effect of both reducing and increasing the number of probes, on the performance (and stability) of the signature with (n=13) and without (n=12) the influential observation contained in the validation set. The microarray and clinical data of the datasets used in this study have been deposited in the Gene Expression Omnibus (GEO) repository under accession number GSE107754. First, we attempted to reduce the number of probes by using only probes from the original signature with a positive regression coefficient (≥1). Specifically from these probes, we tested by 10-fold CV (in the training set) the reduced signatures containing the 49, 99, 149, 199, 249 and 332 probes with the highest positive regression coefficients to estimate the RMSE of each of these reduced signatures. Among these signatures the 2 with the lowest RMSE were those containing 149 and 249 probes, which by 10-fold CV had the third component as the optimal component to minimize RMSE, and hence those signatures were tested in our validation set.

Second, we tested the signatures with the following thresholds of absolute regression coefficients: 1.3, 1.2, 1.1, 1.0 (the original signature), 0.9 and 0.8. To make it clear, the signature of the 1.3 absolute regression coefficient threshold includes the probes with positive regression coefficients (≥1.3) and also those with negative regression coefficients (≤-1.3) and so on for the rest of the thresholds mentioned. For the thresholds tested the number of probes corresponding to each one was: 174 (for the 1.3 threshold), 323 (for 1.2), 547 (for 1.1), 909 (for 1.0), 1461 (for 0.9) and 2248 (for 0.8). In addition, 10-fold CV of each tested signature was carried out to estimate the number of components required to minimize RMSE in the training set. Only two of the signatures tested required more than 3 components to minimize RMSE: 6 components for the 547 probes signature and 5 components for the 174 probes signature. Making use of additional components in those two signatures did not improve the prediction made with the remainder of signatures that required 3 components to minimize RMSE.

Third, we also tested probes from the original signature with a negative regression coefficient (≤ -1). Specifically from these probes, we tested by 10-fold CV (in the training set) the reduced signatures containing the 101, 201, 301, 401 and 501 probes with the lowest negative regression coefficients to estimate the RMSE of each of these reduced signatures. Among these signatures the 2 with the lowest RMSE were those containing 201 and 301 probes, which by 10-fold CV had the fourth and the third component respectively as the optimal components to minimize RMSE. Those signatures were tested in our validation set. The addition of a fourth component to the 201 probes signature did not significantly improve the prediction performance of this signature.

We also checked and confirmed that by reducing substantially the number of probes (up to 150) of the signature, the performance of the selected model continued being the best one (and statistically significant) as compared with the other models tested, following the same method described above for the 909 probes.

**Supplementary Methods References**

1. Mevik B-H, Wehrens R. The pls Package: Principal Component and Partial Least Squares Regression in R. J Stat Software 2007;18(2):23.

2. Karatzoglou A, Smola A, Hornik K, Zeileis A. kernlab - An S4 Package for Kernel Methods in R. J Stat Software 2004;11(9):20.

3. Breiman L. Random Forests. Machine Learning. 2001;45(1):5-32.

4. Kuhn M. Building Predictive Models in R Using the caret Package. J Stat Software 2008;28(5):26.
