# Supplementary material for: A novel genomic signature predicting FDG uptake in diverse metastatic tumors
Source: EJNMMI Res. 2018 Jan 18;8:4. doi: 10.1186/s13550-017-0355-3 (PMC5773462; doi:10.1186/s13550-017-0355-3)
Supplement: Supplementary file 2 — Complete list of the 909 probes selected for the generation of the multivariable model along with their correspondent regression coefficient. (DOCX 110 kb) [file 13550_2017_355_MOESM2_ESM.docx]

**Table S4.** Complete list of the 909 probes selected for the generation of the multivariable model along with their correspondent regression coefficient

| **ProbeID** | **Gene symbol** | **Gene name** | **Regression Coefficient** |
| --- | --- | --- | --- |
| A_23_P134225 | LRRC61 | leucine rich repeat containing 61 | 1,71 |
| A_23_P90130 | NAPSA | napsin A aspartic peptidase | 1,69 |
| A_23_P127533 | DCUN1D5 | DCN1, defective in cullin neddylation 1, domain containing 5 (S. cerevisiae) | 1,68 |
| A_23_P217637 | TIMM8A | translocase of inner mitochondrial membrane 8 homolog A (yeast) | 1,66 |
| A_23_P16908 | DHX57 | DEAH (Asp-Glu-Ala-Asp/His) box polypeptide 57 | 1,62 |
| A_23_P67288 | CALR | calreticulin | 1,62 |
| A_23_P28318 | C2orf56 | chromosome 2 open reading frame 56 | 1,57 |
| A_23_P358417 | TIMM8A | translocase of inner mitochondrial membrane 8 homolog A (yeast) | 1,56 |
| A_24_P653603 | C17orf89 | chromosome 17 open reading frame 89 | 1,55 |
| A_23_P213584 | HK3 | hexokinase 3 (white cell) | 1,54 |
| A_23_P110504 | CLPTM1L | CLPTM1-like | 1,51 |
| A_23_P319719 | PGAM5 | phosphoglycerate mutase family member 5 | 1,51 |
| A_23_P90589 | MRPL44 | mitochondrial ribosomal protein L44 | 1,50 |
| A_23_P211659 | CERK | ceramide kinase | 1,50 |
| A_23_P55190 | EFTUD2 | elongation factor Tu GTP binding domain containing 2 | 1,49 |
| A_23_P29303 | RRP7A | ribosomal RNA processing 7 homolog A (S. cerevisiae) | 1,49 |
| A_23_P77360 | CIAO1 | cytosolic iron-sulfur protein assembly 1 homolog (S. cerevisiae) | 1,48 |
| A_23_P76969 | SIPA1L1 | signal-induced proliferation-associated 1 like 1 | 1,48 |
| A_23_P44195 | MSI2 | musashi homolog 2 (Drosophila) | 1,46 |
| A_23_P211488 | APOL2 | apolipoprotein L, 2 | 1,44 |
| A_24_P71021 | GNB1 | guanine nucleotide binding protein (G protein), beta polypeptide 1 | 1,44 |
| A_23_P150583 | SCGB1A1 | secretoglobin, family 1A, member 1 (uteroglobin) | 1,43 |
| A_32_P171143 | LOC389458 | hypothetical LOC389458 | 1,43 |
| A_24_P37962 | HK3 | hexokinase 3 (white cell) | 1,43 |
| A_23_P321703 | BCL2A1 | BCL2-related protein A1 | 1,42 |
| A_24_P83158 | RRP7B | ribosomal RNA processing 7 homolog B (S. cerevisiae) | 1,42 |
| A_23_P119337 | ATF5 | activating transcription factor 5 | 1,42 |
| A_23_P385034 | E2F3 | E2F transcription factor 3 | 1,42 |
| A_23_P137665 | CHI3L1 | chitinase 3-like 1 (cartilage glycoprotein-39) | 1,41 |
| A_24_P196827 | HLA-DQA1 | major histocompatibility complex, class II, DQ alpha 1 | 1,41 |
| A_23_P253561 | TTPAL | tocopherol (alpha) transfer protein-like | 1,40 |
| A_24_P219785 | CALM3 | calmodulin 3 (phosphorylase kinase, delta) | 1,40 |
| A_23_P98431 | HMBS | hydroxymethylbilane synthase | 1,40 |
| A_23_P51187 | PRKCZ | protein kinase C, zeta | 1,40 |
| A_23_P103885 | AURKAIP1 | aurora kinase A interacting protein 1 | 1,40 |
| A_32_P107029 | NAPSA | napsin A aspartic peptidase | 1,40 |
| A_23_P155106 | CCDC134 | coiled-coil domain containing 134 | 1,39 |
| A_23_P136573 | ST3GAL5 | ST3 beta-galactoside alpha-2,3-sialyltransferase 5 | 1,38 |
| A_23_P82642 | LANCL2 | LanC lantibiotic synthetase component C-like 2 (bacterial) | 1,36 |
| A_23_P40975 | EDEM1 | ER degradation enhancer, mannosidase alpha-like 1 | 1,36 |
| A_32_P194264 | CHAC2 | ChaC, cation transport regulator homolog 2 (E. coli) | 1,36 |
| A_23_P93360 | AGER | advanced glycosylation end product-specific receptor | 1,36 |
| A_23_P168629 | RBM28 | RNA binding motif protein 28 | 1,35 |
| A_23_P42302 | HLA-DQA2 | major histocompatibility complex, class II, DQ alpha 2 | 1,35 |
| A_23_P163630 | ZNF276 | zinc finger protein 276 | 1,34 |
| A_23_P136683 | HLA-DQB1 | major histocompatibility complex, class II, DQ beta 1 | 1,34 |
| A_23_P774 | DENND4B | DENN/MADD domain containing 4B | 1,33 |
| A_23_P17044 | SFTPB | surfactant protein B | 1,33 |
| A_23_P115861 | ZNF485 | zinc finger protein 485 | 1,33 |
| A_24_P274111 | CHI3L1 | chitinase 3-like 1 (cartilage glycoprotein-39) | 1,33 |
| A_23_P165707 | LYG1 | lysozyme G-like 1 | 1,32 |
| A_23_P27894 | SAFB2 | scaffold attachment factor B2 | 1,32 |
| A_23_P76435 | GATC | glutamyl-tRNA(Gln) amidotransferase, subunit C homolog (bacterial) | 1,32 |
| A_32_P122793 | C22orf27 | chromosome 22 open reading frame 27 | 1,32 |
| A_23_P27840 | SUPT5H | suppressor of Ty 5 homolog (S. cerevisiae) | 1,31 |
| A_23_P28068 | GTPBP3 | GTP binding protein 3 (mitochondrial) | 1,31 |
| A_23_P138465 | NOLC1 | nucleolar and coiled-body phosphoprotein 1 | 1,30 |
| A_24_P81789 | RHOF | ras homolog gene family, member F (in filopodia) | 1,30 |
| A_23_P109304 | SLC5A3 | solute carrier family 5 (sodium/myo-inositol cotransporter), member 3 | 1,30 |
| A_23_P369479 | MSI2 | musashi homolog 2 (Drosophila) | 1,30 |
| A_23_P80389 | TRMU | tRNA 5-methylaminomethyl-2-thiouridylate methyltransferase | 1,30 |
| A_32_P102300 |  |  | 1,30 |
| A_23_P116187 | BCO2 | beta-carotene oxygenase 2 | 1,30 |
| A_23_P75978 | CLPB | ClpB caseinolytic peptidase B homolog (E. coli) | 1,29 |
| A_32_P359110 | KLHL17 | kelch-like 17 (Drosophila) | 1,29 |
| A_24_P335620 | SLC7A5 | solute carrier family 7 (cationic amino acid transporter, y+ system), member 5 | 1,29 |
| A_23_P209954 | GNLY | granulysin | 1,29 |
| A_23_P42802 | PDIA4 | protein disulfide isomerase family A, member 4 | 1,28 |
| A_23_P27493 | PPAN | peter pan homolog (Drosophila) | 1,28 |
| A_23_P34325 | LRP8 | low density lipoprotein receptor-related protein 8, apolipoprotein e receptor | 1,27 |
| A_24_P67681 | LOC441795 | high mobility group protein B3-like protein-like | 1,27 |
| A_24_P184230 | AASDHPPT | aminoadipate-semialdehyde dehydrogenase-phosphopantetheinyl transferase | 1,27 |
| A_23_P60517 | FXN | frataxin | 1,27 |
| A_24_P206343 | MYO1G | myosin IG | 1,27 |
| A_24_P289376 | C2orf68 | chromosome 2 open reading frame 68 | 1,26 |
| A_23_P426398 | PGAM5 | phosphoglycerate mutase family member 5 | 1,26 |
| A_24_P51517 | FGR | Gardner-Rasheed feline sarcoma viral (v-fgr) oncogene homolog | 1,26 |
| A_23_P410059 | CIAO1 | cytosolic iron-sulfur protein assembly 1 homolog (S. cerevisiae) | 1,26 |
| A_23_P15174 | MT1F | metallothionein 1F | 1,26 |
| A_23_P418051 | LOC339803 | hypothetical protein LOC339803 | 1,26 |
| A_23_P102117 | WNT10A | wingless-type MMTV integration site family, member 10A | 1,25 |
| A_23_P161297 | OGDHL | oxoglutarate dehydrogenase-like | 1,25 |
| A_24_P31583 | LOC729991-MEF2B | LOC729991-MEF2B readthrough transcript | 1,25 |
| A_24_P303594 | ACTR3B | ARP3 actin-related protein 3 homolog B (yeast) | 1,24 |
| A_32_P224253 | DNAH11 | dynein, axonemal, heavy chain 11 | 1,24 |
| A_23_P64010 | MTCH2 | mitochondrial carrier homolog 2 (C. elegans) | 1,24 |
| A_23_P254340 | RPN1 | ribophorin I | 1,24 |
| A_23_P251248 | TBCC | tubulin folding cofactor C | 1,24 |
| A_23_P104734 | STT3A | STT3, subunit of the oligosaccharyltransferase complex, homolog A (S. cerevisiae) | 1,24 |
| A_23_P316460 | C7orf29 | chromosome 7 open reading frame 29 | 1,24 |
| A_24_P21727 | C11orf57 | chromosome 11 open reading frame 57 | 1,23 |
| A_24_P298846 | C6orf120 | chromosome 6 open reading frame 120 | 1,23 |
| A_23_P336015 | NOC2L | nucleolar complex associated 2 homolog (S. cerevisiae) | 1,23 |
| A_23_P17769 | DDT | D-dopachrome tautomerase | 1,23 |
| A_23_P59507 | SH2B2 | SH2B adaptor protein 2 | 1,23 |
| A_24_P93901 | SIN3B | SIN3 homolog B, transcription regulator (yeast) | 1,23 |
| A_23_P11390 | VCY | variable charge, Y-linked | 1,22 |
| A_23_P161474 | MCM10 | minichromosome maintenance complex component 10 | 1,22 |
| A_23_P1292 | ERCC6 | excision repair cross-complementing rodent repair deficiency, complementation group 6 | 1,22 |
| A_32_P130536 |  |  | 1,22 |
| A_23_P216043 | DERL1 | Der1-like domain family, member 1 | 1,22 |
| A_23_P357780 | FDX1 | ferredoxin 1 | 1,21 |
| A_23_P46928 | PFKP | phosphofructokinase, platelet | 1,21 |
| A_23_P323783 | LSM14B | LSM14B, SCD6 homolog B (S. cerevisiae) | 1,21 |
| A_23_P74688 | ATAD3B | ATPase family, AAA domain containing 3B | 1,20 |
| A_23_P127995 | CLNS1A | chloride channel, nucleotide-sensitive, 1A | 1,20 |
| A_23_P39445 | MEX3D | mex-3 homolog D (C. elegans) | 1,20 |
| A_23_P32463 | EXOSC4 | exosome component 4 | 1,20 |
| A_23_P200222 | LRP8 | low density lipoprotein receptor-related protein 8, apolipoprotein e receptor | 1,19 |
| A_24_P418386 | LOC644422 | similar to arginine/serine-rich splicing factor 6 | 1,19 |
| A_24_P152713 |  |  | 1,19 |
| A_24_P781757 | LOC148413 | hypothetical LOC148413 | 1,19 |
| A_32_P93391 | NUDT19 | nudix (nucleoside diphosphate linked moiety X)-type motif 19 | 1,18 |
| A_23_P211302 | WDR4 | WD repeat domain 4 | 1,18 |
| A_24_P344053 | SIPA1L1 | signal-induced proliferation-associated 1 like 1 | 1,18 |
| A_23_P417363 | CLIP4 | CAP-Gly domain containing linker protein family member 4 | 1,18 |
| A_23_P24068 | BMS1 | BMS1 homolog, ribosome assembly protein (yeast) | 1,18 |
| A_23_P25194 | HRK | harakiri, BCL2 interacting protein (contains only BH3 domain) | 1,18 |
| A_32_P192842 | LOC100293208 | hypothetical protein LOC100293208 | 1,17 |
| A_24_P250666 | SFTPC | surfactant protein C | 1,17 |
| A_23_P86002 | TMED5 | transmembrane emp24 protein transport domain containing 5 | 1,17 |
| A_23_P39215 | NUDT19 | nudix (nucleoside diphosphate linked moiety X)-type motif 19 | 1,17 |
| A_23_P102113 | WNT10A | wingless-type MMTV integration site family, member 10A | 1,17 |
| A_23_P122563 | PFDN6 | prefoldin subunit 6 | 1,16 |
| A_23_P116123 | CHEK1 | CHK1 checkpoint homolog (S. pombe) | 1,16 |
| A_24_P222365 | LARP4 | La ribonucleoprotein domain family, member 4 | 1,16 |
| A_23_P168080 | SKIV2L | superkiller viralicidic activity 2-like (S. cerevisiae) | 1,16 |
| A_24_P195714 |  |  | 1,16 |
| A_23_P155123 | CYP2D6 | cytochrome P450, family 2, subfamily D, polypeptide 6 | 1,16 |
| A_23_P168576 | POMZP3 | POM (POM121 homolog, rat) and ZP3 fusion | 1,16 |
| A_23_P206441 | FANCA | Fanconi anemia, complementation group A | 1,16 |
| A_23_P105475 | SLCO1B3 | solute carrier organic anion transporter family, member 1B3 | 1,16 |
| A_23_P25313 | BRI3BP | BRI3 binding protein | 1,16 |
| A_23_P208310 | CD3EAP | CD3e molecule, epsilon associated protein | 1,15 |
| A_24_P464238 |  |  | 1,15 |
| A_23_P65651 | WARS | tryptophanyl-tRNA synthetase | 1,15 |
| A_24_P175347 | DERL1 | Der1-like domain family, member 1 | 1,15 |
| A_24_P52168 |  |  | 1,15 |
| A_24_P92367 | YDJC | YdjC homolog (bacterial) | 1,15 |
| A_23_P2601 | HSP90B1 | heat shock protein 90kDa beta (Grp94), member 1 | 1,15 |
| A_32_P207360 | AK2 | adenylate kinase 2 | 1,15 |
| A_32_P8402 | SYNCRIP | synaptotagmin binding, cytoplasmic RNA interacting protein | 1,14 |
| A_24_P67063 |  |  | 1,14 |
| A_24_P413437 | NONO | non-POU domain containing, octamer-binding | 1,14 |
| A_23_P126727 | NOL9 | nucleolar protein 9 | 1,14 |
| A_23_P15123 | UBFD1 | ubiquitin family domain containing 1 | 1,14 |
| A_23_P119143 | ICAM5 | intercellular adhesion molecule 5, telencephalin | 1,14 |
| A_24_P390668 | FMNL1 | formin-like 1 | 1,14 |
| A_24_P162929 | FAM119A | family with sequence similarity 119, member A | 1,14 |
| A_23_P103172 | TYW3 | tRNA-yW synthesizing protein 3 homolog (S. cerevisiae) | 1,14 |
| A_23_P119677 | HNRNPL | heterogeneous nuclear ribonucleoprotein L | 1,14 |
| A_23_P206707 | MT1G | metallothionein 1G | 1,14 |
| A_24_P917951 | NOC2L | NOC2 like nucleolar associated transcriptional repressor | 1,14 |
| A_23_P126103 | CTH | cystathionase (cystathionine gamma-lyase) | 1,13 |
| A_23_P19712 | GMNN | geminin, DNA replication inhibitor | 1,13 |
| A_23_P74290 | GBP5 | guanylate binding protein 5 | 1,13 |
| A_23_P95027 | ANKRD39 | ankyrin repeat domain 39 | 1,13 |
| A_23_P97365 | LRRC8D | leucine rich repeat containing 8 family, member D | 1,13 |
| A_23_P422193 | SUV39H1 | suppressor of variegation 3-9 homolog 1 (Drosophila) | 1,13 |
| A_24_P108451 | GPI | glucose phosphate isomerase | 1,13 |
| A_23_P119617 | SF4 | splicing factor 4 | 1,12 |
| A_32_P115122 | LOC100289373 | similar to hCG2041645 | 1,12 |
| A_23_P383278 | PYCRL | pyrroline-5-carboxylate reductase-like | 1,12 |
| A_23_P329286 | ZNHIT2 | zinc finger, HIT type 2 | 1,12 |
| A_23_P53288 | CNPY2 | canopy 2 homolog (zebrafish) | 1,12 |
| A_24_P24685 | LOC729595 | similar to high mobility group box 3 | 1,12 |
| A_23_P201357 | ATAD3A | ATPase family, AAA domain containing 3A | 1,12 |
| A_23_P8513 | SNX10 | sorting nexin 10 | 1,12 |
| A_24_P298877 | C1orf174 | chromosome 1 open reading frame 174 | 1,12 |
| A_23_P74097 | TCEB3 | transcription elongation factor B (SIII), polypeptide 3 (110kDa, elongin A) | 1,12 |
| A_32_P62342 | GXYLT1 | glucoside xylosyltransferase 1 | 1,12 |
| A_23_P399292 |  |  | 1,12 |
| A_23_P115523 | JMJD4 | jumonji domain containing 4 | 1,12 |
| A_23_P373119 | HMGB3L1 | high-mobility group box 3-like 1 | 1,11 |
| A_23_P41528 | FGFBP2 | fibroblast growth factor binding protein 2 | 1,11 |
| A_23_P101992 | MARCO | macrophage receptor with collagenous structure | 1,11 |
| A_24_P50937 | LOC100128842 | hypothetical protein LOC100128842 | 1,11 |
| A_23_P49972 | CDC6 | cell division cycle 6 homolog (S. cerevisiae) | 1,11 |
| A_23_P60943 | TIMM13 | translocase of inner mitochondrial membrane 13 homolog (yeast) | 1,11 |
| A_23_P15202 | DHODH | dihydroorotate dehydrogenase | 1,11 |
| A_23_P136787 | GINS4 | GINS complex subunit 4 (Sld5 homolog) | 1,11 |
| A_23_P120270 | MCFD2 | multiple coagulation factor deficiency 2 | 1,11 |
| A_23_P47735 | RELT | RELT tumor necrosis factor receptor | 1,11 |
| A_23_P165301 | RPE | ribulose-5-phosphate-3-epimerase | 1,11 |
| A_23_P339098 | SLC35F2 | solute carrier family 35, member F2 | 1,11 |
| A_24_P389994 | NADK | NAD kinase | 1,11 |
| A_23_P108054 | TMEM86B | transmembrane protein 86B | 1,11 |
| A_23_P150092 | SEPHS1 | selenophosphate synthetase 1 | 1,11 |
| A_32_P8653 | TPRG1-AS1 | TPRG1 antisense RNA 1 | 1,10 |
| A_23_P314151 | NOLC1 | nucleolar and coiled-body phosphoprotein 1 | 1,10 |
| A_23_P379630 | SLC38A10 | solute carrier family 38, member 10 | 1,10 |
| A_24_P20292 | B3GNT7 | UDP-GlcNAc:betaGal beta-1,3-N-acetylglucosaminyltransferase 7 | 1,10 |
| A_23_P130304 | TXNL4A | thioredoxin-like 4A | 1,10 |
| A_23_P82316 | PRKRIP1 | PRKR interacting protein 1 (IL11 inducible) | 1,10 |
| A_23_P311869 | ST3GAL5 | ST3 beta-galactoside alpha-2,3-sialyltransferase 5 | 1,10 |
| A_23_P212458 | SEC61A1 | Sec61 alpha 1 subunit (S. cerevisiae) | 1,10 |
| A_23_P133216 | NLN | neurolysin (metallopeptidase M3 family) | 1,10 |
| A_32_P235159 | MSL3L2 | male-specific lethal 3-like 2 (Drosophila) | 1,10 |
| A_23_P52298 | NPM3 | nucleophosmin/nucleoplasmin 3 | 1,09 |
| A_23_P100469 | TXNL4B | thioredoxin-like 4B | 1,09 |
| A_23_P74115 | RAD54L | RAD54-like (S. cerevisiae) | 1,09 |
| A_23_P132027 | SPAG4 | sperm associated antigen 4 | 1,09 |
| A_23_P253421 | AUP1 | ancient ubiquitous protein 1 | 1,09 |
| A_24_P521544 |  |  | 1,09 |
| A_23_P47426 | ACAD8 | acyl-Coenzyme A dehydrogenase family, member 8 | 1,09 |
| A_23_P257542 | MYO1G | myosin IG | 1,09 |
| A_23_P164650 | APOE | apolipoprotein E | 1,08 |
| A_23_P90587 | HS6ST1 | heparan sulfate 6-O-sulfotransferase 1 | 1,08 |
| A_23_P138058 | NOC2L | nucleolar complex associated 2 homolog (S. cerevisiae) | 1,08 |
| A_23_P56567 | GEMIN6 | gem (nuclear organelle) associated protein 6 | 1,08 |
| A_23_P217236 | HMGB3 | high-mobility group box 3 | 1,08 |
| A_23_P414771 |  |  | 1,08 |
| A_23_P163380 | MTHFS | 5,10-methenyltetrahydrofolate synthetase (5-formyltetrahydrofolate cyclo-ligase) | 1,08 |
| A_23_P79416 | FBXO11 | F-box protein 11 | 1,08 |
| A_23_P28375 | GMCL1 | germ cell-less homolog 1 (Drosophila) | 1,08 |
| A_23_P257593 | LOH12CR1 | loss of heterozygosity, 12, chromosomal region 1 | 1,08 |
| A_24_P376339 | CCNL2 | cyclin L2 | 1,08 |
| A_32_P105773 | SERBP1 | SERPINE1 mRNA binding protein 1 | 1,08 |
| A_23_P35021 | B3GALT6 | UDP-Gal:betaGal beta 1,3-galactosyltransferase polypeptide 6 | 1,08 |
| A_24_P257099 | HJURP | Holliday junction recognition protein | 1,08 |
| A_23_P19510 | HLA-DQB2 | major histocompatibility complex, class II, DQ beta 2 | 1,08 |
| A_24_P85099 | HMGA2 | high mobility group AT-hook 2 | 1,08 |
| A_23_P143535 | WDR4 | WD repeat domain 4 | 1,07 |
| A_23_P75647 | HYOU1 | hypoxia up-regulated 1 | 1,07 |
| A_24_P133671 | ZNF276 | zinc finger protein 276 | 1,07 |
| A_23_P104798 | IL18 | interleukin 18 (interferon-gamma-inducing factor) | 1,07 |
| A_24_P118247 | CTU2 | cytosolic thiouridylase subunit 2 homolog (S. pombe) | 1,07 |
| A_32_P137336 | MPP6 | membrane palmitoylated protein 6 | 1,07 |
| A_32_P353677 | ATAD3B | ATPase family, AAA domain containing 3B | 1,07 |
| A_23_P251771 | PUS1 | pseudouridylate synthase 1 | 1,07 |
| A_23_P65481 | TEP1 | telomerase-associated protein 1 | 1,07 |
| A_24_P595223 | MIAT | myocardial infarction associated transcript (non-protein coding) | 1,07 |
| A_23_P145006 | SCGB3A2 | secretoglobin, family 3A, member 2 | 1,07 |
| A_23_P107994 | TMEM160 | transmembrane protein 160 | 1,06 |
| A_23_P68970 | ARFGAP3 | ADP-ribosylation factor GTPase activating protein 3 | 1,06 |
| A_23_P93082 | NUS1 | nuclear undecaprenyl pyrophosphate synthase 1 homolog (S. cerevisiae) | 1,06 |
| A_23_P106562 | GALNS | galactosamine (N-acetyl)-6-sulfate sulfatase | 1,06 |
| A_24_P139773 | PPP1R8 | protein phosphatase 1, regulatory (inhibitor) subunit 8 | 1,06 |
| A_23_P105212 | THRSP | thyroid hormone responsive (SPOT14 homolog, rat) | 1,06 |
| A_23_P202715 | PHF21A | PHD finger protein 21A | 1,06 |
| A_24_P51588 | CCNL2 | cyclin L2 | 1,06 |
| A_24_P123347 | PPAT | phosphoribosyl pyrophosphate amidotransferase | 1,05 |
| A_24_P76288 |  |  | 1,05 |
| A_24_P213034 |  |  | 1,05 |
| A_24_P406693 | P4HA1 | prolyl 4-hydroxylase, alpha polypeptide I | 1,05 |
| A_32_P27327 |  |  | 1,05 |
| A_23_P95213 | SFTPC | surfactant protein C | 1,05 |
| A_23_P258071 | RNF113A | ring finger protein 113A | 1,05 |
| A_23_P41255 | G3BP2 | GTPase activating protein (SH3 domain) binding protein 2 | 1,05 |
| A_23_P369987 | KIAA0317 | KIAA0317 | 1,05 |
| A_32_P172917 | LOC100288755 | hypothetical LOC100288755 | 1,05 |
| A_23_P97952 | C10orf125 | chromosome 10 open reading frame 125 | 1,05 |
| A_23_P206369 | TMEM208 | transmembrane protein 208 | 1,05 |
| A_23_P203075 | USP28 | ubiquitin specific peptidase 28 | 1,05 |
| A_24_P204515 |  |  | 1,05 |
| A_32_P12065 |  |  | 1,05 |
| A_23_P27656 | C19orf48 | chromosome 19 open reading frame 48 | 1,05 |
| A_23_P117933 | GCSH | glycine cleavage system protein H (aminomethyl carrier) | 1,05 |
| A_23_P60479 | DNAJA1 | DnaJ (Hsp40) homolog, subfamily A, member 1 | 1,05 |
| A_23_P315336 | TAPBP | TAP binding protein (tapasin) | 1,05 |
| A_23_P73992 | USP24 | ubiquitin specific peptidase 24 | 1,05 |
| A_24_P63262 | RPN1 | ribophorin I | 1,05 |
| A_32_P139654 | SS18L1 | synovial sarcoma translocation gene on chromosome 18-like 1 | 1,04 |
| A_24_P4212 | PYCRL | pyrroline-5-carboxylate reductase-like | 1,04 |
| A_24_P105164 | MAGT1 | magnesium transporter 1 | 1,04 |
| A_23_P50455 | POLD1 | polymerase (DNA directed), delta 1, catalytic subunit 125kDa | 1,04 |
| A_24_P331711 | THEM5 | thioesterase superfamily member 5 | 1,04 |
| A_23_P4798 | ZNF581 | zinc finger protein 581 | 1,04 |
| A_32_P86739 | C10orf114 | chromosome 10 open reading frame 114 | 1,04 |
| A_32_P98979 | LRP8 | LDL receptor related protein 8 (LRP8) | 1,04 |
| A_23_P214425 | NFYA | nuclear transcription factor Y, alpha | 1,04 |
| A_23_P108641 | FAM136A | family with sequence similarity 136, member A | 1,04 |
| A_24_P943843 |  |  | 1,03 |
| A_23_P334103 | LOH12CR1 | loss of heterozygosity, 12, chromosomal region 1 | 1,03 |
| A_23_P162256 | DENR | density-regulated protein | 1,03 |
| A_23_P149099 | DDOST | dolichyl-diphosphooligosaccharide-protein glycosyltransferase | 1,03 |
| A_23_P345139 | C19orf28 | chromosome 19 open reading frame 28 | 1,03 |
| A_24_P272073 |  |  | 1,03 |
| A_23_P142380 | AKAP8 | A kinase (PRKA) anchor protein 8 | 1,03 |
| A_23_P155049 | APOL6 | apolipoprotein L, 6 | 1,03 |
| A_32_P93807 |  |  | 1,03 |
| A_23_P41280 | PAICS | phosphoribosylaminoimidazole carboxylase, phosphoribosylaminoimidazole succinocarboxamide synthetase | 1,03 |
| A_24_P307424 |  |  | 1,03 |
| A_24_P77364 | BOLA3 | bolA homolog 3 (E. coli) | 1,03 |
| A_24_P878419 | LOC645360 | high mobility group box 3 pseudogene 5 | 1,03 |
| A_24_P944040 | RRP1B | ribosomal RNA processing 1 homolog B (S. cerevisiae) | 1,03 |
| A_23_P71053 | MPP6 | membrane protein, palmitoylated 6 (MAGUK p55 subfamily member 6) | 1,03 |
| A_24_P298464 |  |  | 1,03 |
| A_23_P74914 | URB2 | URB2 ribosome biogenesis 2 homolog (S. cerevisiae) | 1,03 |
| A_23_P50357 | ARHGEF18 | Rho/Rac guanine nucleotide exchange factor (GEF) 18 | 1,02 |
| A_23_P202988 | AASDHPPT | aminoadipate-semialdehyde dehydrogenase-phosphopantetheinyl transferase | 1,02 |
| A_23_P123022 | YWHAG | tyrosine 3-monooxygenase/tryptophan 5-monooxygenase activation protein, gamma polypeptide | 1,02 |
| A_24_P405054 | C1orf144 | chromosome 1 open reading frame 144 | 1,02 |
| A_23_P130900 | NCLN | nicalin homolog (zebrafish) | 1,02 |
| A_24_P412088 | MCM10 | minichromosome maintenance complex component 10 | 1,02 |
| A_23_P80129 | RRP1 | ribosomal RNA processing 1 homolog (S. cerevisiae) | 1,02 |
| A_23_P145817 | C7orf30 | chromosome 7 open reading frame 30 | 1,02 |
| A_23_P28105 | TSN | translin | 1,02 |
| A_23_P59528 | ACN9 | ACN9 homolog (S. cerevisiae) | 1,02 |
| A_23_P132260 | PES1 | pescadillo homolog 1, containing BRCT domain (zebrafish) | 1,02 |
| A_24_P780609 | LOC390618 | ribosomal protein L7 like 1 pseudogene | 1,02 |
| A_23_P156842 | EEF1E1 | eukaryotic translation elongation factor 1 epsilon 1 | 1,02 |
| A_23_P58337 | FIP1L1 | FIP1 like 1 (S. cerevisiae) | 1,02 |
| A_23_P207905 | SECTM1 | secreted and transmembrane 1 | 1,02 |
| A_24_P64401 | LOC84856 | hypothetical LOC84856 | 1,02 |
| A_23_P101392 | TMEM38A | transmembrane protein 38A | 1,01 |
| A_23_P104651 | CDCA5 | cell division cycle associated 5 | 1,01 |
| A_23_P143242 | CEBPB | CCAAT/enhancer binding protein (C/EBP), beta | 1,01 |
| A_23_P336040 | GOPC | golgi associated PDZ and coiled-coil motif containing | 1,01 |
| A_23_P165171 | TMEM161A | transmembrane protein 161A | 1,01 |
| A_32_P213002 |  |  | 1,01 |
| A_23_P203463 | TAF10 | TAF10 RNA polymerase II, TATA box binding protein (TBP)-associated factor, 30kDa | 1,01 |
| A_23_P115246 | FCN3 | ficolin (collagen/fibrinogen domain containing) 3 (Hakata antigen) | 1,01 |
| A_23_P103631 | EBNA1BP2 | EBNA1 binding protein 2 | 1,01 |
| A_24_P282363 | KIAA1875 | KIAA1875 | 1,01 |
| A_32_P169353 |  |  | 1,01 |
| A_23_P20732 | GTF3C4 | general transcription factor IIIC, polypeptide 4, 90kDa | 1,01 |
| A_32_P107617 | SFTPD | surfactant protein D | 1,01 |
| A_23_P89798 | FECH | ferrochelatase (protoporphyria) | 1,01 |
| A_24_P103803 | B4GALT1 | UDP-Gal:betaGlcNAc beta 1,4- galactosyltransferase, polypeptide 1 | 1,01 |
| A_32_P149967 |  |  | 1,01 |
| A_24_P941167 | APOL6 | apolipoprotein L, 6 | 1,01 |
| A_23_P409386 | SLC25A22 | solute carrier family 25 (mitochondrial carrier: glutamate), member 22 | 1,01 |
| A_24_P367397 |  |  | 1,01 |
| A_24_P358116 |  |  | 1,01 |
| A_23_P80362 | NHP2L1 | NHP2 non-histone chromosome protein 2-like 1 (S. cerevisiae) | 1,01 |
| A_24_P42122 | HNRNPL | heterogeneous nuclear ribonucleoprotein L | 1,00 |
| A_24_P12932 | MRPS16 | mitochondrial ribosomal protein S16 | 1,00 |
| A_24_P38895 | H2AFX | H2A histone family, member X | 1,00 |
| A_23_P393620 | TFPI2 | tissue factor pathway inhibitor 2 | 1,00 |
| A_24_P277295 | RAB43 | RAB43, member RAS oncogene family | 1,00 |
| A_24_P916195 | GTSE1 | G-2 and S-phase expressed 1 | 1,00 |
| A_23_P48886 | ADAM10 | ADAM metallopeptidase domain 10 | 1,00 |
| A_23_P145024 | ADRB2 | adrenergic, beta-2-, receptor, surface | -1,00 |
| A_23_P37497 | MYO1E | Human myosin-IC mRNA, complete cds. [U14391] | -1,00 |
| A_23_P96285 | REEP1 | receptor accessory protein 1 | -1,00 |
| A_23_P29257 | H1F0 | H1 histone family, member 0 | -1,00 |
| A_23_P218965 | LARS | leucyl-tRNA synthetase | -1,00 |
| A_23_P252403 | COMMD10 | COMM domain containing 10 | -1,00 |
| A_23_P35414 | PPP1R3C | protein phosphatase 1, regulatory (inhibitor) subunit 3C | -1,00 |
| A_23_P87421 | PRSS23 | protease, serine 23 | -1,00 |
| A_24_P295379 | GPR107 | G protein-coupled receptor 107 | -1,00 |
| A_23_P151368 | N6AMT2 | N-6 adenine-specific DNA methyltransferase 2 (putative) | -1,00 |
| A_23_P166633 | ITGB5 | integrin, beta 5 | -1,00 |
| A_23_P125157 | NFATC2IP | nuclear factor of activated T-cells, cytoplasmic, calcineurin-dependent 2 interacting protein | -1,00 |
| A_23_P381449 | SP2 | Sp2 transcription factor | -1,00 |
| A_23_P372255 | ITPKB | inositol 1,4,5-trisphosphate 3-kinase B | -1,00 |
| A_24_P12181 | UBTD2 | ubiquitin domain containing 2 | -1,00 |
| A_24_P830667 | RPL21 | ribosomal protein L21 | -1,00 |
| A_23_P136777 | APOD | apolipoprotein D | -1,01 |
| A_23_P71867 | IL11RA | interleukin 11 receptor, alpha | -1,01 |
| A_24_P575336 | RPL6P3 | ribosomal protein L6 pseudogene 3 | -1,01 |
| A_23_P108157 | TJP3 | tight junction protein 3 (zona occludens 3) | -1,01 |
| A_23_P157527 | LRRCC1 | leucine rich repeat and coiled-coil domain containing 1 | -1,01 |
| A_24_P25354 | TACC1 | transforming, acidic coiled-coil containing protein 1 | -1,01 |
| A_32_P147324 |  |  | -1,01 |
| A_23_P347131 | TRAPPC12 | tetratricopeptide repeat domain 15 | -1,01 |
| A_24_P84808 | RPL7P50 | ribosomal protein L7 pseudogene 50 | -1,01 |
| A_23_P304682 | EMP2 | epithelial membrane protein 2 | -1,01 |
| A_23_P90762 | STK39 | serine threonine kinase 39 (STE20/SPS1 homolog, yeast) | -1,01 |
| A_24_P142151 | LRRC28 | leucine rich repeat containing 28 | -1,01 |
| A_24_P264772 | PPIAL4A | peptidylprolyl isomerase A (cyclophilin A)-like 4A | -1,01 |
| A_24_P850187 |  |  | -1,01 |
| A_24_P157342 | C3orf10 | chromosome 3 open reading frame 10 | -1,01 |
| A_23_P367610 | SESTD1 | SEC14 and spectrin domains 1 | -1,01 |
| A_23_P170273 | SEC31A | SEC31 homolog A (S. cerevisiae) | -1,01 |
| A_32_P42574 | C1orf198 | chromosome 1 open reading frame 198 | -1,01 |
| A_23_P43337 | FREM1 | FRAS1 related extracellular matrix 1 | -1,01 |
| A_24_P940125 | CNOT6 | CCR4-NOT transcription complex, subunit 6 | -1,01 |
| A_23_P44505 | KLF11 | Kruppel-like factor 11 | -1,01 |
| A_23_P110712 | DUSP1 | dual specificity phosphatase 1 | -1,01 |
| A_23_P218928 | FAM198B | family with sequence similarity 198, member B | -1,01 |
| A_23_P374695 | TEK | TEK tyrosine kinase, endothelial | -1,01 |
| A_23_P140928 | TMC7 | transmembrane channel-like 7 | -1,01 |
| A_32_P4608 |  |  | -1,01 |
| A_23_P344421 | ROBO4 | roundabout homolog 4, magic roundabout (Drosophila) | -1,01 |
| A_24_P127159 | LOC346329 | similar to guanine nucleotide binding protein, alpha 11 | -1,01 |
| A_24_P29885 | NOV | nephroblastoma overexpressed gene | -1,02 |
| A_24_P944570 | PXDN | peroxidasin homolog (Drosophila) | -1,02 |
| A_24_P56270 | DYRK2 | dual-specificity tyrosine-(Y)-phosphorylation regulated kinase 2 | -1,02 |
| A_24_P296772 | PPP1R14A | protein phosphatase 1, regulatory (inhibitor) subunit 14A | -1,02 |
| A_23_P7655 | BNIP1 | BCL2/adenovirus E1B 19kDa interacting protein 1 | -1,02 |
| A_23_P134827 | ASH2L | ash2 (absent, small, or homeotic)-like (Drosophila) | -1,02 |
| A_23_P305292 | LOC728264 | hypothetical LOC728264 | -1,02 |
| A_23_P501831 | C5orf4 | chromosome 5 open reading frame 4 | -1,02 |
| A_23_P47077 | BAG3 | BCL2-associated athanogene 3 | -1,02 |
| A_24_P923113 | TMOD3 | tropomodulin 3 (ubiquitous) | -1,02 |
| A_23_P311192 | SPTLC1 | serine palmitoyltransferase, long chain base subunit 1 | -1,02 |
| A_23_P253345 | C8orf4 | chromosome 8 open reading frame 4 | -1,02 |
| A_32_P137632 | FBXL17 | F-box and leucine-rich repeat protein 17 | -1,02 |
| A_23_P395493 | CFLP1 | cofilin pseudogene 1 | -1,02 |
| A_32_P59475 | MYL9 | Homo sapiens myosin light chain 9 | -1,02 |
| A_23_P30307 | MED7 | mediator complex subunit 7 | -1,02 |
| A_23_P24260 | ENTPD1 | ectonucleoside triphosphate diphosphohydrolase 1 | -1,02 |
| A_24_P157424 | NCBP2 | nuclear cap binding protein subunit 2, 20kDa | -1,02 |
| A_32_P50834 | DIAPH2 | diaphanous homolog 2 (Drosophila) | -1,02 |
| A_23_P205841 | MYO9A | myosin IXA | -1,02 |
| A_23_P111583 | CD36 | CD36 molecule (thrombospondin receptor) | -1,02 |
| A_23_P106617 | WFDC1 | WAP four-disulfide core domain 1 | -1,03 |
| A_24_P221375 |  |  | -1,03 |
| A_32_P135818 | RPS3A | ribosomal protein S3A | -1,03 |
| A_24_P288993 | LOC402175 | hypothetical gene supported by AF044957; NM_004547; NADH dehydrogenase (ubiquinone) 1 beta subcomplex, 4, 15kDa | -1,03 |
| A_23_P257144 | PXDN | peroxidasin homolog (Drosophila) | -1,03 |
| A_23_P114839 | FHL3 | four and a half LIM domains 3 | -1,03 |
| A_24_P615462 |  |  | -1,03 |
| A_23_P23194 | PINK1 | PTEN induced putative kinase 1 | -1,03 |
| A_23_P133543 | KLHL3 | kelch-like 3 (Drosophila) | -1,03 |
| A_24_P75008 | LOC346329 | similar to guanine nucleotide binding protein, alpha 11 | -1,03 |
| A_24_P193295 | RAB15 | RAB15, member RAS onocogene family | -1,03 |
| A_23_P201386 | DDAH1 | dimethylarginine dimethylaminohydrolase 1 | -1,03 |
| A_23_P123848 | DAB2IP | DAB2 interacting protein | -1,03 |
| A_23_P393425 | PAPD4 | PAP associated domain containing 4 | -1,03 |
| A_24_P186764 |  |  | -1,03 |
| A_23_P207939 | C18orf1 | chromosome 18 open reading frame 1 | -1,03 |
| A_24_P237778 | MAN1C1 | mannosidase, alpha, class 1C, member 1 | -1,03 |
| A_23_P27649 | ZNF433 | zinc finger protein 433 | -1,03 |
| A_24_P25137 | CHRM3 | cholinergic receptor, muscarinic 3 | -1,03 |
| A_23_P58796 | RGMB | RGM domain family, member B | -1,03 |
| A_23_P81399 | SQSTM1 | sequestosome 1 | -1,04 |
| A_23_P18017 | CPA3 | carboxypeptidase A3 (mast cell) | -1,04 |
| A_23_P70060 | PPAP2A | phosphatidic acid phosphatase type 2A | -1,04 |
| A_32_P3385 | FLJ37798 | hypothetical gene supported by AK095117 | -1,04 |
| A_23_P43276 | GPR124 | G protein-coupled receptor 124 | -1,04 |
| A_24_P341279 | TRAK1 | trafficking protein, kinesin binding 1 | -1,04 |
| A_23_P335452 | ZCCHC24 | zinc finger, CCHC domain containing 24 | -1,04 |
| A_23_P432583 | OAZ3 | ornithine decarboxylase antizyme 3 | -1,04 |
| A_24_P358305 |  |  | -1,04 |
| A_23_P151870 | GLCE | glucuronic acid epimerase | -1,04 |
| A_32_P40288 | TMEM200A | transmembrane protein 200A | -1,04 |
| A_23_P391906 | TMEM200A | transmembrane protein 200A | -1,04 |
| A_24_P89971 | SURF4 | surfeit 4 | -1,04 |
| A_23_P344973 | MYL6 | myosin, light chain 6, alkali, smooth muscle and non-muscle | -1,04 |
| A_24_P68222 | CD99P1 | CD99 molecule pseudogene 1 | -1,04 |
| A_24_P354523 | AIMP1 | aminoacyl tRNA synthetase complex-interacting multifunctional protein 1 | -1,04 |
| A_23_P133582 | ETF1 | eukaryotic translation termination factor 1 | -1,04 |
| A_23_P14184 | THSD1 | thrombospondin, type I, domain containing 1 | -1,04 |
| A_24_P313186 | CALM1 | calmodulin 1 (phosphorylase kinase, delta) | -1,04 |
| A_23_P170733 | ANTXR2 | anthrax toxin receptor 2 | -1,04 |
| A_23_P50426 | KANK2 | KN motif and ankyrin repeat domains 2 | -1,04 |
| A_32_P32722 | LOC729678 | hypothetical protein LOC729678 | -1,04 |
| A_23_P99920 | HACD3 | 3-hydroxyacyl-CoA dehydratase 3 | -1,04 |
| A_23_P70719 | LAMA2 | laminin, alpha 2 | -1,04 |
| A_24_P153803 | RHOJ | ras homolog gene family, member J | -1,04 |
| A_24_P377144 | ANTXR2 | anthrax toxin receptor 2 | -1,05 |
| A_24_P316939 | LRRFIP1 | leucine rich repeat (in FLII) interacting protein 1 | -1,05 |
| A_23_P361014 | TSHZ3 | teashirt zinc finger homeobox 3 | -1,05 |
| A_24_P226970 | ZNF365 | zinc finger protein 365 | -1,05 |
| A_23_P316974 | SYNJ2 | synaptojanin 2 | -1,05 |
| A_24_P37519 | LZTFL1 | leucine zipper transcription factor-like 1 | -1,05 |
| A_23_P74359 | CSRP1 | cysteine and glycine-rich protein 1 | -1,05 |
| A_23_P215459 | ELN | elastin | -1,05 |
| A_24_P260582 | TRIM52 | tripartite motif-containing 52 | -1,05 |
| A_24_P192840 |  |  | -1,05 |
| A_24_P920319 | ZBED5 | zinc finger BED-type containing 5 | -1,05 |
| A_23_P147245 | OSBPL10 | oxysterol binding protein-like 10 | -1,05 |
| A_23_P89343 | SNX11 | sorting nexin 11 | -1,05 |
| A_23_P14124 | RASL11A | RAS-like, family 11, member A | -1,05 |
| A_24_P646168 |  |  | -1,05 |
| A_24_P220485 | OLFML2A | olfactomedin-like 2A | -1,05 |
| A_23_P58390 | C4orf32 | chromosome 4 open reading frame 32 | -1,05 |
| A_24_P186065 | DHFRL1 | dihydrofolate reductase-like 1 | -1,05 |
| A_24_P11791 | KPNA4 | karyopherin alpha 4 (importin alpha 3) | -1,06 |
| A_23_P55107 | ULK2 | unc-51-like kinase 2 (C. elegans) | -1,06 |
| A_23_P2831 | EDNRB | endothelin receptor type B | -1,06 |
| A_24_P304760 | SIN3A | SIN3 homolog A, transcription regulator (yeast) | -1,06 |
| A_23_P206661 | NQO1 | NAD(P)H dehydrogenase, quinone 1 | -1,06 |
| A_24_P15765 | RPS7P5 | ribosomal protein S7 pseudogene 5 | -1,06 |
| A_24_P399980 | HEPH | hephaestin | -1,06 |
| A_32_P514599 |  |  | -1,06 |
| A_23_P70867 | SBDS | Shwachman-Bodian-Diamond syndrome | -1,06 |
| A_23_P92025 | CIDEC | cell death-inducing DFFA-like effector c | -1,06 |
| A_24_P100673 | TMEM85 | transmembrane protein 85 | -1,06 |
| A_24_P69691 | ZNF25 | zinc finger protein 25 | -1,06 |
| A_23_P82990 | OGN | osteoglycin | -1,06 |
| A_23_P213944 | HBEGF | heparin-binding EGF-like growth factor | -1,06 |
| A_23_P77859 | TMEM88 | transmembrane protein 88 | -1,06 |
| A_23_P77145 | RAB11A | RAB11A, member RAS oncogene family | -1,06 |
| A_23_P113777 | ITGBL1 | integrin, beta-like 1 (with EGF-like repeat domains) | -1,06 |
| A_23_P156880 | ENPP1 | ectonucleotide pyrophosphatase/phosphodiesterase 1 | -1,06 |
| A_23_P134167 | PDSS2 | prenyl (decaprenyl) diphosphate synthase, subunit 2 | -1,06 |
| A_23_P103765 | FCER1A | Fc fragment of IgE, high affinity I, receptor for; alpha polypeptide | -1,06 |
| A_23_P70020 | PFDN1 | prefoldin subunit 1 | -1,06 |
| A_24_P811014 |  |  | -1,06 |
| A_23_P4223 | CALCOCO2 | calcium binding and coiled-coil domain 2 | -1,06 |
| A_23_P212535 | CHMP2B | chromatin modifying protein 2B | -1,07 |
| A_24_P213487 | SMG1P2 | PI-3-kinase-related kinase SMG-1 pseudogene | -1,07 |
| A_32_P24832 | OLFML3 | olfactomedin-like 3 | -1,07 |
| A_23_P112652 | CNOT10 | CCR4-NOT transcription complex, subunit 10 | -1,07 |
| A_23_P252283 | RNF135 | ring finger protein 135 | -1,07 |
| A_32_P220472 | ZFAND6 | zinc finger, AN1-type domain 6 | -1,07 |
| A_24_P538567 |  |  | -1,07 |
| A_24_P92973 | LOC400061 | hypothetical gene supported by NM_014886 | -1,07 |
| A_23_P151267 | LIMA1 | LIM domain and actin binding 1 | -1,07 |
| A_23_P421638 | ZBTB47 | zinc finger and BTB domain containing 47 | -1,07 |
| A_24_P226554 | ACTB | actin, beta | -1,07 |
| A_23_P85004 | DIAPH2 | diaphanous homolog 2 (Drosophila) | -1,07 |
| A_24_P286054 | ZFYVE16 | zinc finger, FYVE domain containing 16 | -1,07 |
| A_23_P132595 | VGLL4 | vestigial like 4 (Drosophila) | -1,07 |
| A_24_P391431 | TAF9B | TAF9B RNA polymerase II, TATA box binding protein (TBP)-associated factor, 31kDa | -1,07 |
| A_23_P258582 | GK5 | glycerol kinase 5 (putative) | -1,07 |
| A_24_P6903 | ACTBL2 | actin, beta-like 2 | -1,07 |
| A_24_P566701 | SKP1 | S-phase kinase-associated protein 1 | -1,08 |
| A_24_P393312 | KIRREL | kin of IRRE like (Drosophila) | -1,08 |
| A_23_P30275 | PCYOX1L | prenylcysteine oxidase 1 like | -1,08 |
| A_23_P415401 | KLF9 | Kruppel-like factor 9 | -1,08 |
| A_32_P123168 | MYO9A | myosin IXA | -1,08 |
| A_23_P213966 | TIGD6 | tigger transposable element derived 6 | -1,08 |
| A_24_P117866 | ADD1 | adducin 1 (alpha) | -1,08 |
| A_24_P76210 |  |  | -1,08 |
| A_23_P95823 | NSMCE1 | non-SMC element 1 homolog (S. cerevisiae) | -1,08 |
| A_24_P298834 | RPL32P10 | ribosomal protein L32 pseudogene 10 | -1,08 |
| A_24_P917457 |  |  | -1,08 |
| A_24_P229766 |  |  | -1,08 |
| A_23_P96965 | SYNC | syncoilin, intermediate filament protein | -1,08 |
| A_23_P50535 | DMPK | dystrophia myotonica-protein kinase | -1,08 |
| A_23_P387630 | STARD8 | StAR-related lipid transfer (START) domain containing 8 | -1,08 |
| A_32_P56525 | FAM115A | family with sequence similarity 115, member A | -1,08 |
| A_23_P212728 | TBC1D23 | TBC1 domain family, member 23 | -1,08 |
| A_23_P51397 | ENAH | enabled homolog (Drosophila) | -1,08 |
| A_24_P83544 | TCEB1P3 | transcription elongation factor B subunit 1 pseudogene 3 | -1,08 |
| A_23_P381577 | ZNF25 | zinc finger protein 25 | -1,08 |
| A_23_P6891 | EIF1B | eukaryotic translation initiation factor 1B | -1,08 |
| A_24_P592012 | ZBTB46 | zinc finger and BTB domain containing 46 | -1,08 |
| A_23_P318420 | CTTNBP2NL | CTTNBP2 N-terminal like | -1,09 |
| A_23_P348383 | CC2D2A | coiled-coil and C2 domain containing 2A | -1,09 |
| A_24_P381555 | SAP18 | Sin3A-associated protein, 18kDa | -1,09 |
| A_23_P140648 | CYFIP1 | cytoplasmic FMR1 interacting protein 1 | -1,09 |
| A_24_P497244 | MALAT1 | metastasis associated lung adenocarcinoma transcript 1 (non-protein coding) | -1,09 |
| A_23_P45106 | QRICH1 | glutamine-rich 1 | -1,09 |
| A_32_P14610 | PDLIM5 | PDZ and LIM domain 5 | -1,09 |
| A_24_P11315 | OLFML3 | olfactomedin-like 3 | -1,09 |
| A_23_P502343 | ADAM33 | ADAM metallopeptidase domain 33 | -1,09 |
| A_24_P253676 |  |  | -1,09 |
| A_23_P212768 | ADD1 | adducin 1 (alpha) | -1,09 |
| A_32_P192376 | ENPP1 | ectonucleotide pyrophosphatase/phosphodiesterase 1 | -1,09 |
| A_24_P56130 | MYL6 | myosin, light chain 6, alkali, smooth muscle and non-muscle | -1,09 |
| A_23_P128230 | NR4A1 | nuclear receptor subfamily 4, group A, member 1 | -1,09 |
| A_23_P118705 | SDF2 | stromal cell-derived factor 2 | -1,09 |
| A_24_P290585 | UACA | uveal autoantigen with coiled-coil domains and ankyrin repeats | -1,09 |
| A_23_P94889 | FAM82B | family with sequence similarity 82, member B | -1,09 |
| A_23_P383910 | KIAA1191 | KIAA1191 | -1,09 |
| A_24_P173754 | C1orf21 | chromosome 1 open reading frame 21 | -1,10 |
| A_24_P931944 | PARVA | parvin alpha | -1,10 |
| A_23_P58622 | UBE2D2 | ubiquitin-conjugating enzyme E2D 2 (UBC4/5 homolog, yeast) | -1,10 |
| A_24_P113926 | DPT | dermatopontin | -1,10 |
| A_23_P19142 | KCNMB1 | potassium large conductance calcium-activated channel, subfamily M, beta member 1 | -1,10 |
| A_23_P500364 | BCL7B | B-cell CLL/lymphoma 7B | -1,10 |
| A_23_P8185 | DYNLT1 | dynein, light chain, Tctex-type 1 | -1,10 |
| A_23_P93442 | SASH1 | SAM and SH3 domain containing 1 | -1,10 |
| A_23_P256473 | SEMA3C | sema domain, immunoglobulin domain (Ig), short basic domain, secreted, (semaphorin) 3C | -1,10 |
| A_23_P39237 | ZFP36 | zinc finger protein 36, C3H type, homolog (mouse) | -1,10 |
| A_24_P102203 | UBR1 | ubiquitin protein ligase E3 component n-recognin 1 | -1,10 |
| A_23_P163455 | MAP1A | microtubule-associated protein 1A | -1,10 |
| A_23_P385206 | STX12 | syntaxin 12 | -1,10 |
| A_24_P79712 | FAM36A | family with sequence similarity 36, member A | -1,10 |
| A_24_P929570 | RAPH1 | Ras association (RalGDS/AF-6) and pleckstrin homology domains 1 | -1,10 |
| A_24_P388810 | SRP19 | signal recognition particle 19kDa | -1,10 |
| A_24_P753161 | BMPR2 | bone morphogenetic protein receptor, type II (serine/threonine kinase) | -1,10 |
| A_23_P137909 | HIST3H3 | histone cluster 3, H3 | -1,10 |
| A_24_P86993 | JAM3 | junctional adhesion molecule 3 | -1,10 |
| A_24_P114551 | LPP | LIM domain containing preferred translocation partner in lipoma | -1,10 |
| A_23_P423012 | DUSP27 | dual specificity phosphatase 27 (putative) | -1,10 |
| A_24_P179044 | SNX9 | sorting nexin 9 | -1,10 |
| A_23_P315892 | ST6GALNAC6 | ST6 (alpha-N-acetyl-neuraminyl-2,3-beta-galactosyl-1,3)-N-acetylgalactosaminide alpha-2,6-sialyltransferase 6 | -1,11 |
| A_24_P46689 | SASH1 | SAM and SH3 domain containing 1 | -1,11 |
| A_24_P47467 | TMEM109 | transmembrane protein 109 | -1,11 |
| A_24_P943815 | PNPLA4 | patatin-like phospholipase domain containing 4 | -1,11 |
| A_23_P258151 | FGD5 | FYVE, RhoGEF and PH domain containing 5 | -1,11 |
| A_23_P324813 | BCL6B | B-cell CLL/lymphoma 6, member B (zinc finger protein) | -1,11 |
| A_24_P205874 | SEC22C | SEC22 vesicle trafficking protein homolog C (S. cerevisiae) | -1,11 |
| A_24_P71700 | ZBTB47 | zinc finger and BTB domain containing 47 | -1,11 |
| A_24_P368023 | TCAF1 | TRPM8 channel associated factor 1 | -1,11 |
| A_23_P251611 | TBC1D9B | TBC1 domain family, member 9B (with GRAM domain) | -1,11 |
| A_24_P333857 | SGIP1 | SH3-domain GRB2-like (endophilin) interacting protein 1 | -1,11 |
| A_24_P796652 | RBPJP7 | RBPJ pseudogene 7 | -1,11 |
| A_23_P210425 | MYL9 | myosin, light chain 9, regulatory | -1,11 |
| A_24_P277349 | SEC31A | SEC31 homolog A (S. cerevisiae) | -1,11 |
| A_23_P429082 | SPTY2D1 | SPT2, Suppressor of Ty, domain containing 1 (S. cerevisiae) | -1,11 |
| A_23_P391586 | TPM1 | tropomyosin 1 (alpha) | -1,11 |
| A_24_P484797 | CIDECP | cell death-inducing DFFA-like effector c pseudogene | -1,11 |
| A_24_P19544 | STK39 | serine threonine kinase 39 (STE20/SPS1 homolog, yeast) | -1,11 |
| A_23_P21976 | CSPG4 | chondroitin sulfate proteoglycan 4 | -1,11 |
| A_32_P38426 | C10orf46 | chromosome 10 open reading frame 46 | -1,12 |
| A_24_P358205 |  |  | -1,12 |
| A_23_P41512 | HAUS3 | HAUS augmin-like complex, subunit 3 | -1,12 |
| A_24_P143171 | TMEM47 | transmembrane protein 47 | -1,12 |
| A_23_P206018 | TPM1 | tropomyosin 1 (alpha) | -1,12 |
| A_23_P141055 | TGFB1I1 | transforming growth factor beta 1 induced transcript 1 | -1,12 |
| A_23_P214789 | SNX9 | sorting nexin 9 | -1,12 |
| A_23_P16953 | HTR2B | 5-hydroxytryptamine (serotonin) receptor 2B | -1,12 |
| A_23_P14957 | CDR2 | cerebellar degeneration-related protein 2, 62kDa | -1,12 |
| A_23_P124084 | LOXL1 | lysyl oxidase-like 1 | -1,12 |
| A_23_P144827 | FBXL7 | F-box and leucine-rich repeat protein 7 | -1,12 |
| A_24_P111912 | FAM172A | family with sequence similarity 172, member A | -1,12 |
| A_24_P414952 | TMEM168 | transmembrane protein 168 | -1,12 |
| A_23_P133656 | LAMA4 | laminin, alpha 4 | -1,13 |
| A_24_P315256 |  |  | -1,13 |
| A_24_P944827 | ATG7 | ATG7 autophagy related 7 homolog (S. cerevisiae) | -1,13 |
| A_32_P188860 | IL17RD | interleukin 17 receptor D | -1,13 |
| A_24_P398972 | COQ7 | coenzyme Q7 homolog, ubiquinone (yeast) | -1,13 |
| A_23_P121011 | CSRNP1 | cysteine-serine-rich nuclear protein 1 | -1,13 |
| A_32_P137075 | KIAA1217 | Homo sapiens KIAA1217 (KIAA1217) | -1,13 |
| A_23_P256432 | PPP2R5A | protein phosphatase 2, regulatory subunit B', alpha isoform | -1,13 |
| A_24_P291978 | ADCK2 | aarF domain containing kinase 2 | -1,13 |
| A_23_P63908 | TRUB1 | TruB pseudouridine (psi) synthase homolog 1 (E. coli) | -1,13 |
| A_23_P212436 | CTDSPL | CTD (carboxy-terminal domain, RNA polymerase II, polypeptide A) small phosphatase-like | -1,14 |
| A_23_P205910 | SLC24A1 | solute carrier family 24 (sodium/potassium/calcium exchanger), member 1 | -1,14 |
| A_23_P211806 | LRRFIP2 | leucine rich repeat (in FLII) interacting protein 2 | -1,14 |
| A_23_P213562 | F2R | coagulation factor II (thrombin) receptor | -1,14 |
| A_24_P560909 | RNF115 | ring finger protein 115 | -1,14 |
| A_23_P258972 | GOLGA1 | golgi autoantigen, golgin subfamily a, 1 | -1,14 |
| A_24_P362850 | PBRM1 | polybromo 1 | -1,14 |
| A_32_P140656 | IL6ST | interleukin 6 signal transducer (gp130, oncostatin M receptor) | -1,14 |
| A_23_P30614 | PLN | phospholamban | -1,14 |
| A_23_P206140 | DNAJA4 | DnaJ (Hsp40) homolog, subfamily A, member 4 | -1,14 |
| A_24_P296508 | SLC43A2 | solute carrier family 43, member 2 | -1,14 |
| A_23_P21618 | PDZRN3 | PDZ domain containing ring finger 3 | -1,14 |
| A_32_P65371 |  |  | -1,14 |
| A_23_P5550 | PUM2 | pumilio homolog 2 (Drosophila) | -1,14 |
| A_23_P102364 | NGEF | neuronal guanine nucleotide exchange factor | -1,14 |
| A_23_P53623 | P2RX4 | purinergic receptor P2X, ligand-gated ion channel, 4 | -1,14 |
| A_24_P227993 | UBE2I | ubiquitin-conjugating enzyme E2I (UBC9 homolog, yeast) | -1,14 |
| A_23_P61854 | TTC37 | tetratricopeptide repeat domain 37 | -1,14 |
| A_24_P409971 | NEXN | nexilin (F actin binding protein) | -1,14 |
| A_24_P608330 | CTNNB1 | Catenin beta 1 | -1,14 |
| A_23_P426305 | AOC3 | amine oxidase, copper containing 3 (vascular adhesion protein 1) | -1,14 |
| A_24_P131752 | DLG1 | discs, large homolog 1 (Drosophila) | -1,14 |
| A_24_P244800 | NDRG2 | NDRG family member 2 | -1,14 |
| A_24_P925505 | CD36 | CD36=collagen type I/thrombospondin receptor {one exon} [human, mRNA Partial, 369 nt]. [S67044] | -1,14 |
| A_23_P382065 | EMCN | endomucin | -1,14 |
| A_23_P48217 | APOLD1 | apolipoprotein L domain containing 1 | -1,15 |
| A_24_P191971 | SAP30L | SAP30-like | -1,15 |
| A_23_P83277 | IL11RA | interleukin 11 receptor, alpha | -1,15 |
| A_24_P351420 | ZDHHC3 | zinc finger, DHHC-type containing 3 | -1,15 |
| A_24_P85181 | ZFYVE20 | zinc finger, FYVE domain containing 20 | -1,15 |
| A_24_P160874 | DUT | deoxyuridine triphosphatase | -1,15 |
| A_23_P73239 | NCKAP1 | NCK-associated protein 1 | -1,15 |
| A_23_P329353 | CNRIP1 | cannabinoid receptor interacting protein 1 | -1,15 |
| A_24_P587803 |  |  | -1,15 |
| A_32_P226907 | LOC284112 | Homo sapiens cDNA FLJ25640 fis, clone STM04823. [AK098506] | -1,15 |
| A_24_P178106 | YTHDC2 | YTH domain containing 2 | -1,15 |
| A_23_P326319 | C16orf45 | chromosome 16 open reading frame 45 | -1,16 |
| A_24_P152188 | PRICKLE2 | prickle homolog 2 (Drosophila) | -1,16 |
| A_23_P73457 | RUFY1 | RUN and FYVE domain containing 1 | -1,16 |
| A_24_P801197 | LOC283788 | FSHD region gene 1 pseudogene | -1,16 |
| A_24_P419211 | MTMR6 | myotubularin related protein 6 | -1,16 |
| A_24_P643776 |  |  | -1,16 |
| A_23_P26124 | RORA | RAR-related orphan receptor A | -1,16 |
| A_24_P658584 | SASH1 | SAM and SH3 domain containing 1 | -1,16 |
| A_32_P169179 | MSX2P1 | msh homeobox 2 pseudogene 1 | -1,16 |
| A_23_P212339 | FYCO1 | FYVE and coiled-coil domain containing 1 | -1,16 |
| A_24_P123632 |  |  | -1,16 |
| A_23_P77228 | CRTC3 | CREB regulated transcription coactivator 3 | -1,16 |
| A_23_P100602 | TBCD | tubulin folding cofactor D | -1,16 |
| A_23_P213551 | CSNK1A1 | casein kinase 1, alpha 1 | -1,17 |
| A_23_P144896 | PDLIM7 | PDZ and LIM domain 7 (enigma) | -1,17 |
| A_24_P225719 | MOBKL3 | MOB1, Mps One Binder kinase activator-like 3 (yeast) | -1,17 |
| A_23_P7337 | EDNRA | endothelin receptor type A | -1,17 |
| A_32_P6139 | C4orf19 | chromosome 4 open reading frame 19 | -1,17 |
| A_24_P334005 | C1orf123 | chromosome 1 open reading frame 123 | -1,17 |
| A_23_P212475 | SHISA5 | shisa homolog 5 (Xenopus laevis) | -1,17 |
| A_23_P53390 | PTPRB | protein tyrosine phosphatase, receptor type, B | -1,17 |
| A_23_P303671 | ECM2 | extracellular matrix protein 2, female organ and adipocyte specific | -1,17 |
| A_23_P73801 | TCEAL1 | transcription elongation factor A (SII)-like 1 | -1,17 |
| A_24_P75708 | LOC390996 | cofilin 1 pseudogene 6 | -1,17 |
| A_23_P42575 | CALD1 | caldesmon 1 | -1,17 |
| A_23_P160318 | COL16A1 | collagen, type XVI, alpha 1 | -1,17 |
| A_32_P74206 | LOC400707 | uncharacterized LOC400707 | -1,17 |
| A_23_P54953 | SAP30BP | SAP30 binding protein | -1,18 |
| A_24_P140608 | HBEGF | heparin-binding EGF-like growth factor | -1,18 |
| A_23_P52552 | BAG3 | BCL2-associated athanogene 3 | -1,18 |
| A_32_P56001 | CD93 | CD93 molecule | -1,18 |
| A_24_P641673 |  |  | -1,18 |
| A_24_P23411 | ARMCX3 | armadillo repeat containing, X-linked 3 | -1,18 |
| A_23_P69491 | RHOA | ras homolog gene family, member A | -1,18 |
| A_32_P164477 | GLIPR2 | GLI pathogenesis related 2 | -1,18 |
| A_24_P230675 | SOCS2 | suppressor of cytokine signaling 2 | -1,18 |
| A_24_P237686 |  |  | -1,18 |
| A_23_P204979 | MBNL2 | muscleblind-like 2 (Drosophila) | -1,19 |
| A_23_P46045 | RGS5 | regulator of G-protein signaling 5 | -1,19 |
| A_32_P81149 | RPL14 | ribosomal protein L14 | -1,19 |
| A_23_P62901 | BTG2 | BTG family, member 2 | -1,19 |
| A_24_P273726 | MSRB3 | methionine sulfoxide reductase B3 | -1,19 |
| A_23_P27724 | SEPW1 | selenoprotein W, 1 | -1,19 |
| A_23_P216429 | ASPN | asporin | -1,19 |
| A_24_P191781 | PARM1 | prostate androgen-regulated mucin-like protein 1 | -1,19 |
| A_23_P18692 | ADH5 | alcohol dehydrogenase 5 (class III), chi polypeptide | -1,19 |
| A_32_P213418 | PLN | phospholamban | -1,19 |
| A_24_P355267 | SLC25A25 | solute carrier family 25 (mitochondrial carrier; phosphate carrier), member 25 | -1,19 |
| A_23_P57323 | ERG | v-ets erythroblastosis virus E26 oncogene homolog (avian) | -1,20 |
| A_24_P283395 | ADCK2 | aarF domain containing kinase 2 | -1,20 |
| A_23_P251945 | DCTN4 | dynactin 4 (p62) | -1,20 |
| A_24_P75879 | SEPT7P2 | septin 7 pseudogene 2 | -1,20 |
| A_24_P357465 | TP53INP2 | tumor protein p53 inducible nuclear protein 2 | -1,20 |
| A_24_P358245 | LOC158381 | ATPase, Class I, type 8B family pseudogene | -1,20 |
| A_24_P940149 | C2CD2 | C2 calcium-dependent domain containing 2 | -1,20 |
| A_23_P160559 | ECM1 | extracellular matrix protein 1 | -1,21 |
| A_24_P400760 | LOC643454 | adaptor-related protein complex 3, sigma 1 subunit pseudogene | -1,21 |
| A_24_P937405 | PRSS23 | protease, serine, 23 | -1,21 |
| A_23_P119593 | EPHX3 | epoxide hydrolase 3 | -1,21 |
| A_23_P166663 | APPL1 | adaptor protein, phosphotyrosine interaction, PH domain and leucine zipper containing 1 | -1,21 |
| A_32_P70519 | LPP | LIM domain containing preferred translocation partner in lipoma | -1,21 |
| A_23_P433188 | GZF1 | GDNF-inducible zinc finger protein 1 | -1,21 |
| A_23_P150053 | ACTA2 | actin, alpha 2, smooth muscle, aorta | -1,21 |
| A_23_P303833 | SCN4B | sodium channel, voltage-gated, type IV, beta | -1,21 |
| A_24_P362737 | FOXP1 | forkhead box P1 | -1,21 |
| A_23_P148297 | SH3BGRL | SH3 domain binding glutamic acid-rich protein like | -1,22 |
| A_24_P921366 | CALD1 | caldesmon 1 | -1,22 |
| A_24_P943781 | C7orf58 | chromosome 7 open reading frame 58 | -1,22 |
| A_24_P101271 |  |  | -1,22 |
| A_23_P410233 | FILIP1 | filamin A interacting protein 1 | -1,22 |
| A_23_P27584 | MYADM | myeloid-associated differentiation marker | -1,22 |
| A_23_P31124 | COL21A1 | collagen, type XXI, alpha 1 | -1,22 |
| A_32_P68408 | CSNK1A1P | casein kinase 1, alpha 1 pseudogene | -1,22 |
| A_23_P164436 | ASPA | aspartoacylase (Canavan disease) | -1,22 |
| A_24_P214625 | PPA2 | pyrophosphatase (inorganic) 2 | -1,22 |
| A_23_P10902 | FRZB | frizzled-related protein | -1,22 |
| A_24_P410952 | PEA15 | phosphoprotein enriched in astrocytes 15 | -1,22 |
| A_23_P110643 | CDKL3 | cyclin-dependent kinase-like 3 | -1,23 |
| A_23_P24555 | PHLDB1 | pleckstrin homology-like domain, family B, member 1 | -1,23 |
| A_23_P163408 | SCAPER | S-phase cyclin A-associated protein in the ER | -1,23 |
| A_23_P253350 | C8orf4 | chromosome 8 open reading frame 4 | -1,23 |
| A_23_P211985 | SNRK | SNF related kinase | -1,23 |
| A_24_P930796 | C10orf72 | chromosome 10 open reading frame 72 | -1,23 |
| A_23_P201790 | PPP1R12B | protein phosphatase 1, regulatory (inhibitor) subunit 12B | -1,23 |
| A_24_P326511 | SORBS1 | sorbin and SH3 domain containing 1 | -1,23 |
| A_23_P215406 | RAC1 | ras-related C3 botulinum toxin substrate 1 (rho family, small GTP binding protein Rac1) | -1,23 |
| A_24_P375599 |  |  | -1,23 |
| A_23_P152028 | TRIP4 | thyroid hormone receptor interactor 4 | -1,23 |
| A_23_P105144 | SCUBE2 | signal peptide, CUB domain, EGF-like 2 | -1,23 |
| A_24_P583007 | FGD5-AS1 | FGD5 antisense RNA 1 | -1,23 |
| A_23_P12514 | RHOC | ras homolog gene family, member C | -1,23 |
| A_23_P202594 | C10orf119 | chromosome 10 open reading frame 119 | -1,24 |
| A_32_P64928 | LOC100287016 | similar to destrin | -1,24 |
| A_23_P127911 | PAMR1 | peptidase domain containing associated with muscle regeneration 1 | -1,24 |
| A_23_P20566 | TPM2 | tropomyosin 2 (beta) | -1,24 |
| A_23_P257043 | GEM | GTP binding protein overexpressed in skeletal muscle | -1,24 |
| A_24_P175059 | ATG5 | ATG5 autophagy related 5 homolog (S. cerevisiae) | -1,24 |
| A_23_P126706 | ANGPTL1 | angiopoietin-like 1 | -1,24 |
| A_24_P367329 | LOC440292 | similar to COMM domain containing 4 | -1,25 |
| A_24_P375237 | RPL12 | ribosomal protein L12 | -1,25 |
| A_24_P252575 | RABGAP1 | RAB GTPase activating protein 1 | -1,25 |
| A_23_P32233 | KLF4 | Kruppel-like factor 4 (gut) | -1,25 |
| A_24_P191664 | GOLIM4 | golgi integral membrane protein 4 | -1,25 |
| A_32_P128701 | USP53 | ubiquitin specific peptidase 53 | -1,25 |
| A_23_P309865 | ZNF449 | zinc finger protein 449 | -1,25 |
| A_23_P217088 | AK1 | adenylate kinase 1 | -1,25 |
| A_23_P19663 | CTGF | connective tissue growth factor | -1,25 |
| A_23_P43283 | GPR124 | G protein-coupled receptor 124 | -1,26 |
| A_23_P135437 | EXOC4 | exocyst complex component 4 | -1,26 |
| A_23_P144005 | C3orf32 | chromosome 3 open reading frame 32 | -1,26 |
| A_23_P209669 | NRP2 | neuropilin 2 | -1,26 |
| A_32_P183022 | ENAH | enabled homolog (Drosophila) | -1,26 |
| A_23_P429998 | FOSB | FBJ murine osteosarcoma viral oncogene homolog B | -1,26 |
| A_32_P142991 | PRKAR2A | protein kinase, cAMP-dependent, regulatory, type II, alpha | -1,26 |
| A_23_P423543 | REEP3 | receptor accessory protein 3 | -1,26 |
| A_23_P37205 | NDRG2 | NDRG family member 2 | -1,26 |
| A_24_P98249 | TACC1 | transforming, acidic coiled-coil containing protein 1 | -1,26 |
| A_23_P155848 | DKK2 | dickkopf homolog 2 (Xenopus laevis) | -1,26 |
| A_23_P360340 | UACA | uveal autoantigen with coiled-coil domains and ankyrin repeats | -1,26 |
| A_23_P53193 | SYTL2 | synaptotagmin-like 2 | -1,26 |
| A_23_P163458 | EHD4 | EH-domain containing 4 | -1,26 |
| A_23_P148204 | USP47 | ubiquitin specific peptidase 47 | -1,26 |
| A_24_P408424 | MYH9 | myosin, heavy chain 9, non-muscle | -1,27 |
| A_24_P69654 | KLF6 | Kruppel-like factor 6 | -1,27 |
| A_24_P150580 | RASL12 | RAS-like, family 12 | -1,27 |
| A_23_P414273 | C5orf62 | chromosome 5 open reading frame 62 | -1,27 |
| A_23_P112554 | COL15A1 | collagen, type XV, alpha 1 | -1,27 |
| A_32_P100974 | RPL24 | ribosomal protein L24 | -1,27 |
| A_32_P18251 | FUNDC2 | FUN14 domain containing 2 | -1,27 |
| A_32_P220798 | CD34 | CD34 molecule | -1,27 |
| A_24_P329795 | C10orf10 | chromosome 10 open reading frame 10 | -1,27 |
| A_24_P330773 | CALCOCO2 | calcium binding and coiled-coil domain 2 | -1,28 |
| A_24_P59220 | POTEF | POTE ankyrin domain family, member F | -1,28 |
| A_23_P423695 | MXD4 | MAX dimerization protein 4 | -1,28 |
| A_23_P310094 | SYNPO2 | synaptopodin 2 | -1,28 |
| A_24_P251534 | CTDSPL | CTD (carboxy-terminal domain, RNA polymerase II, polypeptide A) small phosphatase-like | -1,28 |
| A_23_P93780 | HGF | hepatocyte growth factor (hepapoietin A; scatter factor) | -1,28 |
| A_23_P102706 | SNPH | syntaphilin | -1,28 |
| A_23_P256334 | ITGA1 | integrin, alpha 1 | -1,28 |
| A_24_P278192 | AK1 | adenylate kinase 1 | -1,29 |
| A_23_P155989 | CENPK | centromere protein K | -1,29 |
| A_23_P316012 | RHOJ | ras homolog gene family, member J | -1,29 |
| A_23_P51518 | RGS5 | regulator of G-protein signaling 5 | -1,29 |
| A_24_P366656 | SH3D19 | SH3 domain containing 19 | -1,29 |
| A_24_P233786 | FAM129A | family with sequence similarity 129, member A | -1,29 |
| A_24_P943613 | TBC1D1 | TBC1 (tre-2/USP6, BUB2, cdc16) domain family, member 1 | -1,29 |
| A_24_P497437 | LOC283788 | FSHD region gene 1 pseudogene | -1,29 |
| A_23_P363778 | FRZB | frizzled-related protein | -1,29 |
| A_24_P867111 | LOC283177 | hypothetical protein LOC283177 | -1,29 |
| A_23_P355295 | FMO2 | flavin containing monooxygenase 2 (non-functional) | -1,30 |
| A_23_P213102 | PALLD | palladin, cytoskeletal associated protein | -1,30 |
| A_32_P19840 |  |  | -1,30 |
| A_23_P79360 | NOSTRIN | nitric oxide synthase trafficker | -1,30 |
| A_24_P315120 | COL15A1 | collagen, type XV, alpha 1 | -1,30 |
| A_23_P107257 | SPOP | speckle-type POZ protein | -1,30 |
| A_23_P373819 | TUSC1 | tumor suppressor candidate 1 | -1,30 |
| A_23_P90062 | DNAJB1 | DnaJ (Hsp40) homolog, subfamily B, member 1 | -1,31 |
| A_32_P167239 | AFAP1L1 | actin filament associated protein 1-like 1 | -1,31 |
| A_24_P126890 | RPL9 | ribosomal protein L9 | -1,31 |
| A_24_P916965 | ITGBL1 | integrin subunit beta like 1 | -1,31 |
| A_23_P132536 | TRAK1 | trafficking protein, kinesin binding 1 | -1,32 |
| A_23_P154740 | PLK1S1 | polo-like kinase 1 substrate 1 | -1,32 |
| A_24_P90774 | UACA | uveal autoantigen with coiled-coil domains and ankyrin repeats | -1,32 |
| A_23_P67299 | DOCK6 | dedicator of cytokinesis 6 | -1,32 |
| A_24_P943802 | SEC31A | SEC31 homolog A (S. cerevisiae) | -1,32 |
| A_24_P401321 |  |  | -1,32 |
| A_23_P144807 | 08-sep | septin 8 | -1,32 |
| A_23_P17152 |  |  | -1,33 |
| A_23_P87013 | TAGLN | transgelin | -1,33 |
| A_24_P134955 | PPP2R5A | protein phosphatase 2, regulatory subunit B', alpha isoform | -1,33 |
| A_24_P217572 | EDNRA | endothelin receptor type A | -1,33 |
| A_23_P6746 | IQSEC1 | IQ motif and Sec7 domain 1 | -1,34 |
| A_32_P7721 | RORA | RAR-related orphan receptor A | -1,34 |
| A_32_P133884 | TUSC1 | tumor suppressor candidate 1 | -1,34 |
| A_24_P168925 | CHRDL1 | chordin-like 1 | -1,34 |
| A_23_P23017 | C1orf123 | chromosome 1 open reading frame 123 | -1,34 |
| A_24_P124370 | PARVA | parvin, alpha | -1,35 |
| A_23_P211816 | MAP4 | microtubule-associated protein 4 | -1,35 |
| A_23_P72387 | AFAP1 | actin filament associated protein 1 | -1,35 |
| A_23_P250930 | CRBN | cereblon | -1,35 |
| A_23_P86461 | PLAC9 | placenta-specific 9 | -1,35 |
| A_32_P43349 | LOC400456 | hypothetical protein LOC400456 | -1,35 |
| A_24_P84408 |  |  | -1,36 |
| A_23_P431933 | CAMKK1 | calcium/calmodulin-dependent protein kinase kinase 1, alpha | -1,36 |
| A_23_P13740 | NAV3 | neuron navigator 3 | -1,36 |
| A_24_P39101 | KCTD10 | potassium channel tetramerisation domain containing 10 | -1,36 |
| A_32_P213521 | EMCN | endomucin | -1,37 |
| A_23_P395365 | ENTPD2 | ectonucleoside triphosphate diphosphohydrolase 2 | -1,37 |
| A_24_P270890 | SPOP | speckle-type POZ protein | -1,37 |
| A_32_P57717 | LRRFIP1 | Homo sapiens leucine rich repeat (in FLII) interacting protein 1 (LRRFIP1) | -1,37 |
| A_23_P310022 | KIAA1217 | KIAA1217 | -1,37 |
| A_24_P233917 | KIAA0494 | KIAA0494 | -1,37 |
| A_23_P33364 | SH3D19 | SH3 domain containing 19 | -1,37 |
| A_23_P51548 | MGST3 | microsomal glutathione S-transferase 3 | -1,38 |
| A_32_P193218 | LPP | LIM domain containing preferred translocation partner in lipoma | -1,38 |
| A_24_P324787 | KANK2 | KN motif and ankyrin repeat domains 2 | -1,38 |
| A_23_P342654 | PALM2-AKAP2 | PALM2-AKAP2 readthrough transcript | -1,38 |
| A_24_P250964 | PEA15 | phosphoprotein enriched in astrocytes 15 | -1,38 |
| A_23_P53081 | OSBPL5 | oxysterol binding protein-like 5 | -1,38 |
| A_23_P128384 | VPS29 | vacuolar protein sorting 29 homolog (S. cerevisiae) | -1,39 |
| A_24_P212389 | MSRB3 | methionine sulfoxide reductase B3 | -1,40 |
| A_24_P225907 | DPH3 | DPH3, KTI11 homolog (S. cerevisiae) | -1,40 |
| A_23_P423309 | PCDH12 | protocadherin 12 | -1,40 |
| A_23_P103672 | NES | nestin | -1,40 |
| A_32_P191285 |  |  | -1,40 |
| A_24_P375949 | RPSAP11 | ribosomal protein SA pseudogene 11 | -1,41 |
| A_24_P15823 |  |  | -1,41 |
| A_23_P134041 | TBX18 | T-box 18 | -1,41 |
| A_24_P408981 |  |  | -1,41 |
| A_23_P125788 | TCEAL7 | transcription elongation factor A (SII)-like 7 | -1,41 |
| A_24_P206121 | KCNMB1 | potassium large conductance calcium-activated channel, subfamily M, beta member 1 | -1,41 |
| A_32_P230736 | LOC389033 | placenta-specific 9 pseudogene | -1,41 |
| A_24_P690235 |  |  | -1,41 |
| A_24_P925422 |  |  | -1,42 |
| A_23_P301304 | FGFR1 | fibroblast growth factor receptor 1 | -1,42 |
| A_23_P6688 | ANO10 | anoctamin 10 | -1,42 |
| A_24_P832426 | B3GALTL | beta 1,3-galactosyltransferase-like | -1,42 |
| A_23_P63798 | KLF6 | Kruppel-like factor 6 | -1,42 |
| A_23_P217428 | ARHGAP6 | Rho GTPase activating protein 6 | -1,42 |
| A_23_P167017 | POPDC2 | popeye domain containing 2 | -1,43 |
| A_24_P101248 | PARD3B | par-3 family cell polarity regulator beta | -1,43 |
| A_24_P727868 | LOC728003 | similar to vesicle-associated membrane protein-associated protein A | -1,43 |
| A_23_P211973 | NEK11 | NIMA (never in mitosis gene a)- related kinase 11 | -1,43 |
| A_23_P502913 | WDR1 | WD repeat domain 1 | -1,44 |
| A_24_P385280 | ALDH9A1 | aldehyde dehydrogenase 9 family, member A1 | -1,44 |
| A_23_P37910 | MAPK3 | mitogen-activated protein kinase 3 | -1,44 |
| A_23_P408363 | ITGBL1 | integrin, beta-like 1 (with EGF-like repeat domains) | -1,45 |
| A_23_P129144 | GCOM1 | GRINL1A complex locus | -1,45 |
| A_23_P218858 | ABI3BP | ABI family, member 3 (NESH) binding protein | -1,45 |
| A_23_P34915 | ATF3 | activating transcription factor 3 | -1,47 |
| A_23_P10506 | HPGDS | hematopoietic prostaglandin D synthase | -1,47 |
| A_23_P349083 | FCHO2 | FCH domain only 2 | -1,47 |
| A_23_P420361 | C3orf10 | chromosome 3 open reading frame 10 | -1,48 |
| A_23_P201940 | LMOD1 | leiomodin 1 (smooth muscle) | -1,48 |
| A_24_P317907 | SORBS1 | sorbin and SH3 domain containing 1 | -1,49 |
| A_23_P21907 |  |  | -1,49 |
| A_24_P256603 | LYRM7 | Lyrm7 homolog (mouse) | -1,49 |
| A_23_P200928 | NID1 | nidogen 1 | -1,49 |
| A_23_P301521 | KIAA1462 | KIAA1462 | -1,50 |
| A_24_P410797 | KALRN | kalirin, RhoGEF kinase | -1,50 |
| A_32_P221452 | RABL3 | RAB, member of RAS oncogene family-like 3 | -1,50 |
| A_24_P109652 | SGK269 | NKF3 kinase family member | -1,50 |
| A_24_P174641 | PDLIM5 | PDZ and LIM domain 5 | -1,51 |
| A_23_P217832 | FAM129A | family with sequence similarity 129, member A | -1,52 |
| A_23_P58647 | CTNNA1 | catenin (cadherin-associated protein), alpha 1, 102kDa | -1,52 |
| A_24_P272313 | C2orf55 | chromosome 2 open reading frame 55 | -1,53 |
| A_32_P112592 | LOC339524 | hypothetical LOC339524 | -1,55 |
| A_23_P213000 | WDR1 | WD repeat domain 1 | -1,55 |
| A_24_P247175 |  |  | -1,56 |
| A_24_P942773 | SLMAP | sarcolemma associated protein | -1,57 |
| A_32_P118586 | FAM116A | family with sequence similarity 116, member A | -1,59 |
| A_32_P218228 | FAM109B | family with sequence similarity 109, member B | -1,62 |
| A_24_P33895 | ATF3 | activating transcription factor 3 | -1,62 |
| A_24_P47182 | VCL | vinculin | -1,64 |
| A_24_P105933 | VIPR1 | vasoactive intestinal peptide receptor 1 | -1,66 |
| A_32_P89709 | TPM1 | tropomyosin 1 (alpha) | -1,66 |
| A_23_P24215 | TBC1D12 | TBC1 domain family, member 12 | -1,72 |
| A_23_P408095 | DSTN | destrin (actin depolymerizing factor) | -1,72 |
| A_24_P53150 | TRAF7 | TNF receptor-associated factor 7 | -1,73 |
| A_32_P198731 | NEURL1B | neuralized homolog 1B (Drosophila) | -1,74 |
| A_23_P502915 | WDR1 | WD repeat domain 1 | -1,79 |
| A_24_P174367 | PPP1R2 | protein phosphatase 1, regulatory (inhibitor) subunit 2 | -1,81 |
| A_24_P945194 | PDCD6IP | programmed cell death 6 interacting protein | -1,82 |
| A_23_P71316 | RBPMS | RNA binding protein with multiple splicing | -1,83 |
| A_24_P26897 | INPP5A | inositol polyphosphate-5-phosphatase, 40kDa | -1,84 |
| A_23_P92903 | C1QTNF2 | C1q and tumor necrosis factor related protein 2 | -1,85 |
| A_23_P92899 | C1QTNF2 | C1q and tumor necrosis factor related protein 2 | -1,91 |
| A_23_P415827 | 08-sep | septin 8 | -2,14 |
