# Supplementary material for: A novel genomic signature predicting FDG uptake in diverse metastatic tumors
Source: EJNMMI Res. 2018 Jan 18;8:4. doi: 10.1186/s13550-017-0355-3 (PMC5773462; doi:10.1186/s13550-017-0355-3)
Supplement: Supplementary file 3 — Detailed tumor histologies of the patients in the training and validation datasets. (DOCX 17 kb) [file 13550_2017_355_MOESM3_ESM.docx]

**Table S6.** Detailed tumor histologies of the patients in the training and validation datasets.

| **Training set (n=71)** |  |
| --- | --- |
| COLORECTAL CANCER | Colorectal adenocarcinoma |
| COLORECTAL CANCER | Colorectal adenocarcinoma |
| COLORECTAL CANCER | Colorectal adenocarcinoma |
| COLORECTAL CANCER | Colorectal adenocarcinoma |
| COLORECTAL CANCER | Colorectal adenocarcinoma |
| COLORECTAL CANCER | Colorectal adenocarcinoma |
| COLORECTAL CANCER | Colorectal adenocarcinoma |
| COLORECTAL CANCER | Colorectal adenocarcinoma |
| COLORECTAL CANCER | Anal squamous cell carcinoma |
| BREAST CANCER | Breast adenocarcinoma |
| BREAST CANCER | Breast adenocarcinoma |
| BREAST CANCER | Breast adenocarcinoma |
| BREAST CANCER | Invasive ductal carcinoma |
| BREAST CANCER | Invasive ductal carcinoma |
| BREAST CANCER | Invasive ductal carcinoma |
| BREAST CANCER | Invasive ductal carcinoma |
| BREAST CANCER | Medullary breast carcinoma |
| SARCOMA | Angiosarcoma |
| SARCOMA | Leiomyosarcoma |
| SARCOMA | Rhabdomyosarcoma |
| SARCOMA | Rhabdomyosarcoma |
| SARCOMA | Soft tissue sarcoma |
| SARCOMA | Soft tissue sarcoma |
| SARCOMA | Soft tissue sarcoma |
| GENITOURINARY TUMOR | Endometrial adenocarcinoma |
| GENITOURINARY TUMOR | Cervical squamous cell carcinoma |
| GENITOURINARY TUMOR | Testicular germ cell tumor |
| GENITOURINARY TUMOR | Testicular germ cell tumor |
| GENITOURINARY TUMOR | Clear cell vaginal adenocarcinoma |
| GENITOURINARY TUMOR | Cervical squamous cell carcinoma |
| GENITOURINARY TUMOR | Urothelial carcinoma |
| OVARIAN CANCER | Epithelial ovarian cancer |
| OVARIAN CANCER | Epithelial ovarian cancer |
| OVARIAN CANCER | Epithelial ovarian cancer |
| OVARIAN CANCER | Epithelial ovarian cancer |
| OVARIAN CANCER | Epithelial ovarian cancer |
| OVARIAN CANCER | Epithelial ovarian cancer |
| OVARIAN CANCER | Epithelial ovarian cancer |
| LUNG CANCER | Non small cell lung carcinoma |
| LUNG CANCER | Non small cell lung carcinoma |
| LUNG CANCER | Non small cell lung carcinoma |
| LUNG CANCER | Non small cell lung carcinoma |
| LUNG CANCER | Non small cell lung carcinoma |
| LUNG CANCER | Non small cell lung carcinoma |
| LUNG CANCER | Small cell lung carcinoma |
| PANCREATIC CANCER | Pancreatic adenocarcinoma |
| PANCREATIC CANCER | Pancreatic adenocarcinoma |
| PANCREATIC CANCER | Pancreatic adenocarcinoma |
| PANCREATIC CANCER | Pancreatic adenocarcinoma |
| PANCREATIC CANCER | Pancreatic adenocarcinoma |
| PANCREATIC CANCER | Pancreatic adenocarcinoma |
| HEAD AND NECK CANCER | Auditive external meatus squamous cell carcinoma |
| HEAD AND NECK CANCER | Laryngeal squamous cell carcinoma |
| HEAD AND NECK CANCER | Oral cavity squamous cell carcinoma |
| HEAD AND NECK CANCER | Salivary gland carcinoma (cystic adenoid) |
| HEAD AND NECK CANCER | Salivary gland carcinoma |
| ESOPHAGEAL CANCER | Esophageal adenocarcinoma |
| ESOPHAGEAL CANCER | Esophageal adenocarcinoma |
| ESOPHAGEAL CANCER | Esophageal squamous cell carcinoma |
| ESOPHAGEAL CANCER | Esophageal squamous cell carcinoma |
| THYROID CANCER | Medullary thyroid cancer |
| THYROID CANCER | Papillary thyroid cancer |
| BILE DUCT CANCER | Biliary tract cancer |
| BILE DUCT CANCER | Biliary tract cancer |
| CUP | Carcinoma of Unknown Primary |
| GASTRIC CANCER | Gastric adenocarcinoma |
| LYMPHOMA | T-Cell non-Hodgkin lymphoma |
| MELANOMA | Melanoma |
| MESOTHELIOMA | Malignant mesothelioma |
| SKIN CANCER | Merkel cell carcinoma |
| KIDNEY CANCER | Papillary renal cell carcinoma |

| **Validation set (n=13)** |  |
| --- | --- |
| BREAST CANCER | Invasive ductal carcinoma |
| BREAST CANCER | Breast adenocarcinoma |
| GENITOURINARY TUMOR | Endometrial adenocarcinoma |
| OVARIAN CANCER | Epithelial ovarian cancer |
| OVARIAN CANCER | Epithelial ovarian cancer |
| OVARIAN CANCER | Epithelial ovarian cancer |
| LUNG CANCER | Non small cell lung carcinoma |
| PANCREATIC CANCER | Pancreatic adenocarcinoma |
| PANCREATIC CANCER | Pancreatic adenocarcinoma |
| BILE DUCT CANCER | Biliary tract cancer |
| BILE DUCT CANCER | Biliary tract cancer |
| ESOPHAGEAL CANCER | Esophageal adenocarcinoma |
| KIDNEY CANCER | Renal cell carcinoma |
