# Supplementary material for: A novel genomic signature predicting FDG uptake in diverse metastatic tumors
Source: EJNMMI Res. 2018 Jan 18;8:4. doi: 10.1186/s13550-017-0355-3 (PMC5773462; doi:10.1186/s13550-017-0355-3)
Supplement: Supplementary file 4 — Biological processes related to the signature genes (Consensus Pathway Database, release 31 (http://ConsensusPathDB.org)). (DOCX 33 kb) [file 13550_2017_355_MOESM4_ESM.docx]

**Table S1. Biological processes related to the signature genes (*Consensus Pathway Database, release 31*** (<http://ConsensusPathDB.org>)).

Biological processes of the genes with positive correlation with the SUV

| **pathway** | **q-value** | **source** |
| --- | --- | --- |
| Scavenging by Class A Receptors | 0.0022 | Reactome |
| Binding and Uptake of Ligands by Scavenger Receptors | 0.0036 | Reactome |
| Protein processing in endoplasmic reticulum - Homo sapiens (human) | 0.0036 | KEGG |
| Asparagine N-linked glycosylation | 0.0045 | Reactome |
| Translocation of ZAP-70 to Immunological synapse | 0.0233 | Reactome |
| Phosphorylation of CD3 and TCR zeta chains | 0.0272 | Reactome |
| PD-1 signaling | 0.0364 | Reactome |
| Antigen processing and presentation - Homo sapiens (human) | 0.0396 | KEGG |
| CD4 T cell receptor signaling-JNK cascade | 0.0412 | INOH |
| Metabolism of carbohydrates | 0.0468 | Reactome |
| DNA Replication | 0.0550 | Reactome |
| Generation of second messenger molecules | 0.0621 | Reactome |
| ATF6-alpha activates chaperone genes | 0.0629 | Wikipathways |
| CD4 T cell receptor signaling-ERK cascade | 0.0713 | INOH |
| N-Glycan biosynthesis | 0.0715 | EHMN |
| DNA Replication | 0.0715 | Wikipathways |
| Scavenging by Class F Receptors | 0.0715 | Reactome |
| Porphyrin metabolism | 0.0937 | INOH |
| Pentose phosphate cycle | 0.0954 | INOH |
| glycolysis | 0.0954 | HumanCyc |
| Downstream TCR signaling | 0.0954 | Reactome |
| N-Glycan biosynthesis - Homo sapiens (human) | 0.0954 | KEGG |
| Allograft Rejection | 0.0988 | Wikipathways |
| Phagosome - Homo sapiens (human) | 0.0988 | KEGG |
| Vesicle-mediated transport | 0.0988 | Reactome |
| heme biosynthesis | 0.0988 | HumanCyc |
| Heme Biosynthesis | 0.0988 | Wikipathways |
| Mitochondrial iron-sulfur cluster biogenesis | 0.0988 | Reactome |
| Removal of licensing factors from origins | 0.0988 | Reactome |
| Regulation of DNA replication | 0.0988 | Reactome |
| Pentose phosphate pathway - Homo sapiens (human) | 0.1000 | KEGG |
| Insulin-mediated glucose transport | 0.1000 | PID |
| Sulfur relay system - Homo sapiens (human) | 0.1000 | KEGG |
| Acetaminophen Metabolism Pathway | 0.1000 | SMPDB |
| Galactose metabolism - Homo sapiens (human) | 0.1000 | KEGG |
| Asthma - Homo sapiens (human) | 0.1000 | KEGG |
| MHC class II antigen presentation | 0.1030 | Reactome |
| Glycolysis | 0.1030 | Reactome |

Biological processes of the genes with negative correlation with the SUV

| **pathway** | **q-value** | **source** |
| --- | --- | --- |
| Smooth Muscle Contraction | 3.29E-10 | Reactome |
| Muscle contraction | 6.72E-07 | Reactome |
| Stabilization and expansion of the E-cadherin adherens junction | 5.21E-06 | PID |
| RHO GTPases activate CIT | 0.0002 | Reactome |
| RHO GTPases Activate ROCKs | 0.0002 | Reactome |
| Adherens junction - Homo sapiens (human) | 0.0006 | KEGG |
| Regulation of actin cytoskeleton - Homo sapiens (human) | 0.0006 | KEGG |
| RHO GTPases activate PAKs | 0.0008 | Reactome |
| Signaling by Rho GTPases | 0.0008 | Reactome |
| Axon guidance | 0.0008 | Reactome |
| Sema4D induced cell migration and growth-cone collapse | 0.0008 | Reactome |
| E-cadherin signaling in the nascent adherens junction | 0.0008 | PID |
| Arf6 downstream pathway | 0.0008 | PID |
| Developmental Biology | 0.0008 | Reactome |
| Sema4D in semaphorin signaling | 0.0013 | Reactome |
| TGF beta Signaling Pathway | 0.0018 | Wikipathways |
| Signaling by VEGF | 0.0018 | Reactome |
| RHO GTPase Effectors | 0.0033 | Reactome |
| Vascular smooth muscle contraction - Homo sapiens (human) | 0.0033 | KEGG |
| VEGFA-VEGFR2 Pathway | 0.0035 | Reactome |
| Integrin | 0.0037 | INOH |
| N-cadherin signaling events | 0.0046 | PID |
| Signaling events mediated by PRL | 0.0046 | PID |
| MAPK family signaling cascades | 0.0047 | Reactome |
| RHO GTPases activate IQGAPs | 0.0049 | Reactome |
| Focal adhesion - Homo sapiens (human) | 0.0066 | KEGG |
| EPH-Ephrin signaling | 0.0091 | Reactome |
| Regulation of Actin Cytoskeleton | 0.0158 | Wikipathways |
| Semaphorin interactions | 0.0197 | Reactome |
| Androgen receptor signaling pathway | 0.0219 | Wikipathways |
| EPHA-mediated growth cone collapse | 0.0230 | Reactome |
| Regulation of cytoplasmic and nuclear SMAD2/3 signaling | 0.0230 | PID |
| Signaling by Interleukins | 0.0241 | Reactome |
| RHO GTPases activate PKNs | 0.0241 | Reactome |
| E-cadherin signaling in keratinocytes | 0.0248 | PID |
| MAPK1/MAPK3 signaling | 0.0248 | Reactome |
| RHO GTPases Activate WASPs and WAVEs | 0.0253 | Reactome |
| RAF-independent MAPK1/3 activation | 0.0327 | Reactome |
| Laminin interactions | 0.0327 | Reactome |
| Nonsense Mediated Decay (NMD) independent of the Exon Junction Complex (EJC) | 0.0327 | Reactome |
| TGF_beta_Receptor | 0.0327 | NetPath |
| Rho GTPase cycle | 0.0327 | Reactome |
| Physiological and Pathological Hypertrophy of the Heart | 0.0350 | Wikipathways |
| BMP receptor signaling | 0.0387 | PID |
| Signalling by NGF | 0.0398 | Reactome |
| EGFR1 | 0.0398 | NetPath |
| RAF/MAP kinase cascade | 0.0398 | Reactome |
| SHC1 events in EGFR signaling | 0.0398 | Reactome |
| SOS-mediated signalling | 0.0398 | Reactome |
| GRB2 events in EGFR signaling | 0.0398 | Reactome |
| SHC1 events in ERBB2 signaling | 0.0398 | Reactome |
| SHC1 events in ERBB4 signaling | 0.0398 | Reactome |
| GRB2 events in ERBB2 signaling | 0.0398 | Reactome |
| Neurotrophic factor-mediated Trk receptor signaling | 0.0398 | PID |
| FRS-mediated FGFR2 signaling | 0.0398 | Reactome |
| FRS-mediated FGFR1 signaling | 0.0398 | Reactome |
| FRS-mediated FGFR3 signaling | 0.0398 | Reactome |
| FRS-mediated FGFR4 signaling | 0.0398 | Reactome |
| Nonsense Mediated Decay (NMD) enhanced by the Exon Junction Complex (EJC) | 0.0416 | Reactome |
| Nonsense-Mediated Decay (NMD) | 0.0416 | Reactome |
| Signalling to p38 via RIT and RIN | 0.0416 | Reactome |
| ARMS-mediated activation | 0.0416 | Reactome |
| Shigellosis - Homo sapiens (human) | 0.0416 | KEGG |
| VEGFR2 mediated vascular permeability | 0.0418 | Reactome |
| Frs2-mediated activation | 0.0418 | Reactome |
| Integrin-linked kinase signaling | 0.0428 | PID |
| Tight junction - Homo sapiens (human) | 0.0428 | KEGG |
| Prolonged ERK activation events | 0.0434 | Reactome |
| Signaling by Leptin | 0.0437 | Reactome |
| Arf6 trafficking events | 0.0437 | PID |
| agrin in postsynaptic differentiation | 0.0437 | BioCarta |
| Signalling to RAS | 0.0437 | Reactome |
| Dilated cardiomyopathy - Homo sapiens (human) | 0.0437 | KEGG |
| Signaling events mediated by VEGFR1 and VEGFR2 | 0.0437 | PID |
| DAP12 signaling | 0.0437 | Reactome |
| Interleukin receptor SHC signaling | 0.0437 | Reactome |
| D-<i>myo</i>-inositol (1,3,4)-trisphosphate biosynthesis | 0.0441 | HumanCyc |
| 1D-<i>myo</i>-inositol hexakisphosphate biosynthesis II (mammalian) | 0.0441 | HumanCyc |
| mcalpain and friends in cell motility | 0.0443 | BioCarta |
| Spinal Cord Injury | 0.0445 | Wikipathways |
| VEGFR2 mediated cell proliferation | 0.0457 | Reactome |
| Lissencephaly gene (LIS1) in neuronal migration and development | 0.0457 | PID |
| activation of camp-dependent protein kinase pka | 0.0457 | BioCarta |
| erk and pi-3 kinase are necessary for collagen binding in corneal epithelia | 0.0457 | BioCarta |
| Leukocyte transendothelial migration - Homo sapiens (human) | 0.0457 | KEGG |
| LRR FLII-interacting protein 1 (LRRFIP1) activates type I IFN production | 0.0457 | Reactome |
| Gastrin-CREB signalling pathway via PKC and MAPK | 0.0457 | Reactome |
| Eukaryotic Translation Termination | 0.0457 | Reactome |
| CDC42 signaling events | 0.0457 | PID |
| Signalling to ERKs | 0.0464 | Reactome |
| Interleukin-2 signaling | 0.0476 | Reactome |
| rho cell motility signaling pathway | 0.0476 | BioCarta |
| role of -arrestins in the activation and targeting of map kinases | 0.0476 | BioCarta |
| Wnt Signaling Pathway Netpath | 0.0476 | Wikipathways |
| Signaling by ERBB4 | 0.0480 | Reactome |
| Proteoglycans in cancer - Homo sapiens (human) | 0.0480 | KEGG |
| Signaling by SCF-KIT | 0.0480 | Reactome |
| integrin signaling pathway | 0.0480 | BioCarta |
| role of egf receptor transactivation by gpcrs in cardiac hypertrophy | 0.0480 | BioCarta |
| Alpha 6 Beta 4 signaling pathway | 0.0480 | Wikipathways |
| ucalpain and friends in cell spread | 0.0480 | BioCarta |
| Beta-catenin phosphorylation cascade | 0.0480 | Reactome |
| Arrhythmogenic Right Ventricular Cardiomyopathy | 0.0480 | Wikipathways |
| Arrhythmogenic right ventricular cardiomyopathy (ARVC) - Homo sapiens (human) | 0.0480 | KEGG |
| Downstream signaling of activated FGFR2 | 0.0490 | Reactome |
| Downstream signaling of activated FGFR1 | 0.0490 | Reactome |
| Downstream signaling of activated FGFR3 | 0.0490 | Reactome |
| Downstream signaling of activated FGFR4 | 0.0490 | Reactome |
| DAP12 interactions | 0.0490 | Reactome |
| L1CAM interactions | 0.0490 | Reactome |
| Calcium signaling pathway - Homo sapiens (human) | 0.0491 | KEGG |
| Signaling by FGFR3 | 0.0491 | Reactome |
| Signaling by FGFR4 | 0.0491 | Reactome |
| BMP Signalling Pathway | 0.0491 | HumanCyc |
| Degradation of beta catenin | 0.0491 | PID |
| er associated degradation (erad) pathway | 0.0491 | BioCarta |
| CREB phosphorylation through the activation of CaMKK | 0.0491 | Reactome |
| Regulation of ornithine decarboxylase (ODC) | 0.0491 | Reactome |
| Interleukin-3, 5 and GM-CSF signaling | 0.0491 | Reactome |
| Signaling by FGFR1 | 0.0491 | Reactome |
| Degradation of beta-catenin by the destruction complex | 0.0491 | Reactome |
| Signaling by FGFR2 | 0.0491 | Reactome |
| Bacterial invasion of epithelial cells - Homo sapiens (human) | 0.0491 | KEGG |
| Signaling by FGFR | 0.0491 | Reactome |
| roles of arrestin dependent recruitment of src kinases in gpcr signaling | 0.0491 | BioCarta |
| S1P1 pathway | 0.0491 | PID |
| role of mal in rho-mediated activation of srf | 0.0491 | BioCarta |
| Signaling by ERBB2 | 0.0491 | Reactome |
| FCERI mediated MAPK activation | 0.0491 | Reactome |
| NGF signalling via TRKA from the plasma membrane | 0.0491 | Reactome |
| Downstream signal transduction | 0.0491 | Reactome |
| Signaling events mediated by Hepatocyte Growth Factor Receptor (c-Met) | 0.0491 | PID |
| Cytokine Signaling in Immune system | 0.0491 | Reactome |
| Hypertrophy Model | 0.0491 | Wikipathways |
| superpathway of D-<i>myo</i>-inositol (1,4,5)-trisphosphate metabolism | 0.0491 | HumanCyc |
| Phenytoin (Antiarrhythmic) Action Pathway | 0.0491 | SMPDB |
| NrCAM interactions | 0.0491 | Reactome |
| Ubiquinol biosynthesis | 0.0491 | Reactome |
| ubiquinol-10 biosynthesis | 0.0491 | HumanCyc |
| G13 Signaling Pathway | 0.0491 | Wikipathways |
| Striated Muscle Contraction | 0.0491 | Wikipathways |
| Regulation of RAC1 activity | 0.0491 | PID |
| Focal Adhesion | 0.0491 | Wikipathways |
| Hypertrophic cardiomyopathy (HCM) - Homo sapiens (human) | 0.0491 | KEGG |
