# Supplementary figures and images for: A novel genomic signature predicting FDG uptake in diverse metastatic tumors
Source: EJNMMI Res. 2018 Jan 18;8:4. doi: 10.1186/s13550-017-0355-3 (PMC5773462; doi:10.1186/s13550-017-0355-3)

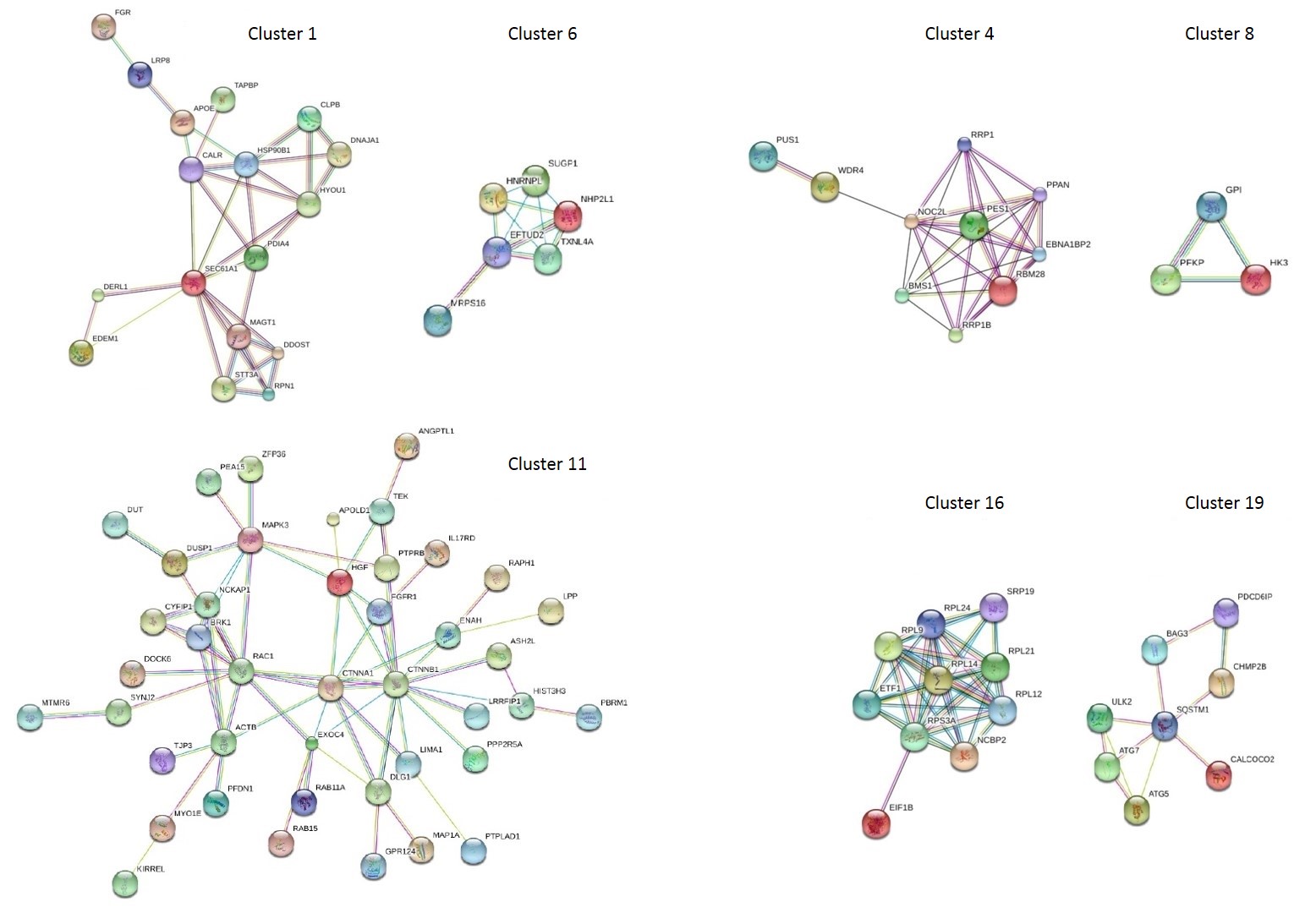

Supplement: Supplementary file 5 — Selected clusters identified in the Protein Protein Interaction (PPI) subnetworks obtained from the signature genes in the STRING 10.0 PPI database. Figure S2. Goodness of fit of PLS-3 in the training set. a) Goodnes of fit including Pearson correlation of measured vs predicted SUV values b) Residuals of third component. No pattern is apparent in the residuals distribution c) Estimated goodness of fit after 10-fold CV. (ZIP 242 kb) [file 13550_2017_355_MOESM5_ESM.zip › Suplementary Figure 1 and 2/Suplementary Figure 1.jpg]

## Slide 1
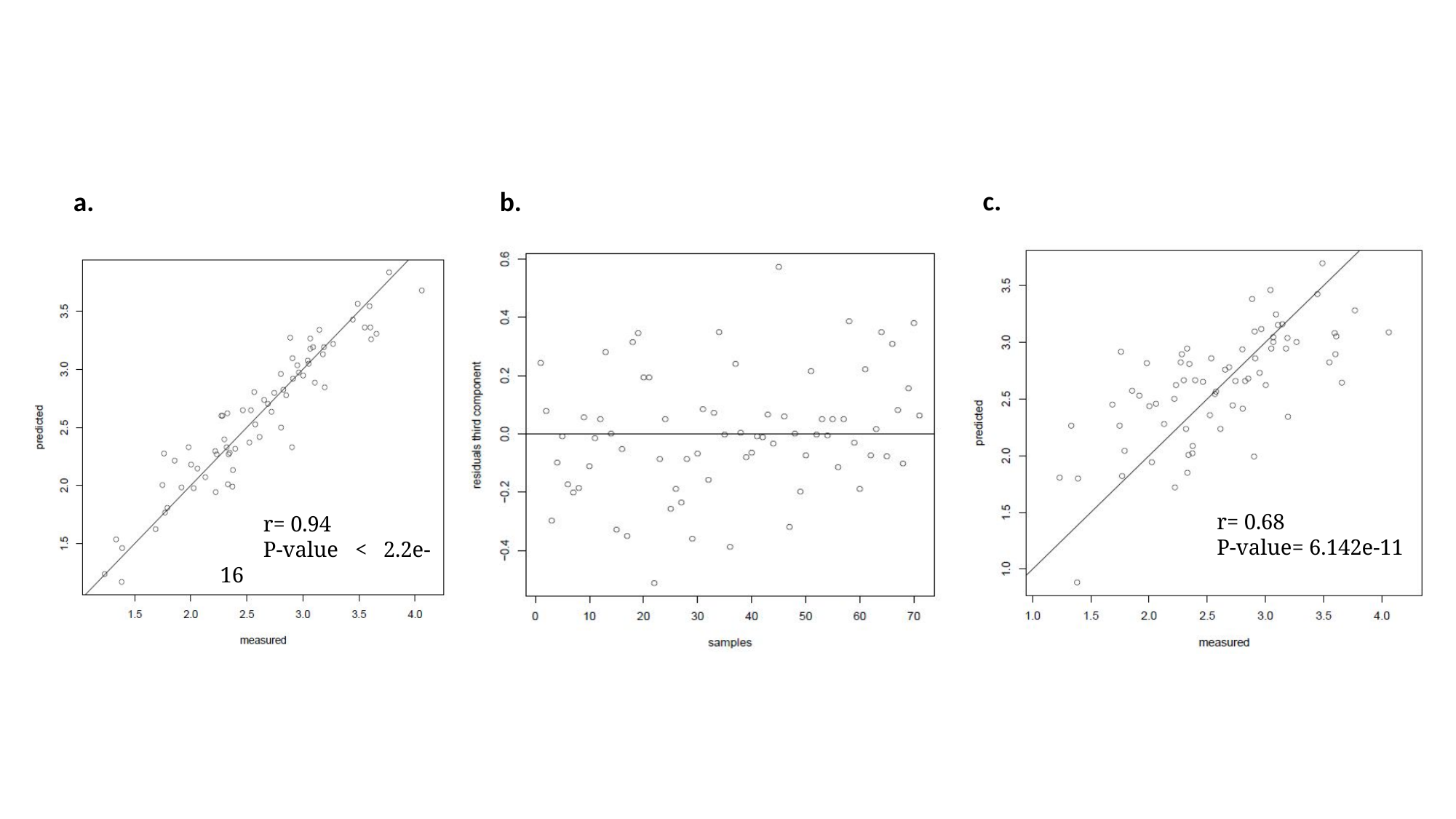

c.
b.
a.
r= 0.68
P-value= 6.142e-11
r= 0.94
P-value < 2.2e-16

Supplement: Supplementary file 5 — Selected clusters identified in the Protein Protein Interaction (PPI) subnetworks obtained from the signature genes in the STRING 10.0 PPI database. Figure S2. Goodness of fit of PLS-3 in the training set. a) Goodnes of fit including Pearson correlation of measured vs predicted SUV values b) Residuals of third component. No pattern is apparent in the residuals distribution c) Estimated goodness of fit after 10-fold CV. (ZIP 242 kb) [file 13550_2017_355_MOESM5_ESM.zip › Suplementary Figure 1 and 2/Supplementary Figure 2.pptx]
