# Supplementary material for: A novel genomic signature predicting FDG uptake in diverse metastatic tumors
Source: EJNMMI Res. 2018 Jan 18;8:4. doi: 10.1186/s13550-017-0355-3 (PMC5773462; doi:10.1186/s13550-017-0355-3)
Supplement: Supplementary file 6 — Correlation coefficient (CC) on SUV of ssGSEA scores with the C2 subset from the MSigDB v5.1 in the training dataset (p < 0.05) (DOCX 31 kb) [file 13550_2017_355_MOESM6_ESM.docx]

**Table S2. Correlation coefficient (CC) on SUV of ssGSEA scores with the C2 subset from the MSigDB v5.1 in the training dataset (p<0.05)**

| **Curated gene sets signatures (C2)** | **CC** | **p-value** |
| --- | --- | --- |
| BIOCARTA_PTEN_PATHWAY | -0.3956 | 0.0006 |
| SMID_BREAST_CANCER_LUMINAL_A_UP | -0.3845 | 0.0009 |
| KEGG_VASCULAR_SMOOTH_MUSCLE_CONTRACTION | -0.3827 | 0.001 |
| PID_AR_TF_PATHWAY | -0.3816 | 0.001 |
| KEGG_CALCIUM_SIGNALING_PATHWAY | -0.3673 | 0.0016 |
| CHANDRAN_METASTASIS_DN | -0.3588 | 0.0021 |
| BIOCARTA_ECM_PATHWAY | -0.3508 | 0.0027 |
| PID_LYSOPHOSPHOLIPID_PATHWAY | -0.3461 | 0.0031 |
| VANTVEER_BREAST_CANCER_BRCA1_DN | -0.3404 | 0.0037 |
| PID_S1P_S1P3_PATHWAY | -0.3395 | 0.0038 |
| ST_INTEGRIN_SIGNALING_PATHWAY | -0.3382 | 0.0039 |
| REACTOME_AMINE_LIGAND_BINDING_RECEPTORS | -0.338 | 0.0039 |
| PID_ARF6_TRAFFICKING_PATHWAY | -0.3304 | 0.0049 |
| ASTON_MAJOR_DEPRESSIVE_DISORDER_DN | -0.3301 | 0.0049 |
| PID_ERBB4_PATHWAY | -0.3275 | 0.0053 |
| LOPEZ_TRANSLATION_VIA_FN1_SIGNALING | -0.3255 | 0.0056 |
| REACTOME_PI3K_EVENTS_IN_ERBB4_SIGNALING | -0.3232 | 0.006 |
| FERRARI_RESPONSE_TO_FENRETINIDE_DN | -0.3218 | 0.0062 |
| DOANE_RESPONSE_TO_ANDROGEN_DN | -0.3206 | 0.0064 |
| OUELLET_CULTURED_OVARIAN_CANCER_INVASIVE_VS_LMP_DN | -0.3187 | 0.0068 |
| ZHENG_GLIOBLASTOMA_PLASTICITY_DN | -0.3186 | 0.0068 |
| KYNG_WERNER_SYNDROM_UP | -0.3178 | 0.0069 |
| BERTUCCI_MEDULLARY_VS_DUCTAL_BREAST_CANCER_DN | -0.3161 | 0.0072 |
| BIOCARTA_AKAP13_PATHWAY | -0.3156 | 0.0073 |
| BROWN_MYELOID_CELL_DEVELOPMENT_DN | 0.3152 | 0.0074 |
| RUAN_RESPONSE_TO_TNF_UP | 0.3144 | 0.0076 |
| KEGG_GNRH_SIGNALING_PATHWAY | -0.314 | 0.0077 |
| BHAT_ESR1_TARGETS_NOT_VIA_AKT1_DN | -0.3135 | 0.0078 |
| DER_IFN_GAMMA_RESPONSE_DN | -0.3116 | 0.0082 |
| PID_TRKR_PATHWAY | -0.3106 | 0.0084 |
| PID_ENDOTHELIN_PATHWAY | -0.3101 | 0.0085 |
| REACTOME_NUCLEAR_RECEPTOR_TRANSCRIPTION_PATHWAY | -0.3101 | 0.0085 |
| MULLIGAN_NTF3_SIGNALING_VIA_INSR_AND_IGF1R_DN | -0.3068 | 0.0093 |
| REACTOME_TRYPTOPHAN_CATABOLISM | 0.3042 | 0.0099 |
| CHEBOTAEV_GR_TARGETS_UP | -0.3008 | 0.0108 |
| ACEVEDO_FGFR1_TARGETS_IN_PROSTATE_CANCER_MODEL_DN | -0.2989 | 0.0113 |
| ONKEN_UVEAL_MELANOMA_DN | -0.2975 | 0.0117 |
| GARGALOVIC_RESPONSE_TO_OXIDIZED_PHOSPHOLIPIDS_GREY_UP | -0.2975 | 0.0117 |
| REACTOME_SIGNALING_BY_ERBB4 | -0.2971 | 0.0119 |
| MYLLYKANGAS_AMPLIFICATION_HOT_SPOT_5 | 0.2964 | 0.0121 |
| REACTOME_SIGNALING_BY_ERBB2 | -0.2957 | 0.0123 |
| PARENT_MTOR_SIGNALING_DN | -0.2931 | 0.0131 |
| PID_RET_PATHWAY | -0.2931 | 0.0131 |
| KEGG_GALACTOSE_METABOLISM | 0.2924 | 0.0134 |
| BIOCARTA_CXCR4_PATHWAY | -0.2919 | 0.0135 |
| RIZKI_TUMOR_INVASIVENESS_3D_DN | 0.2906 | 0.014 |
| REACTOME_CGMP_EFFECTS | -0.2905 | 0.014 |
| IZADPANAH_STEM_CELL_ADIPOSE_VS_BONE_DN | -0.2902 | 0.0141 |
| KEGG_MTOR_SIGNALING_PATHWAY | -0.2888 | 0.0146 |
| LOPEZ_MESOTHELIOMA_SURVIVAL_WORST_VS_BEST_UP | -0.2884 | 0.0147 |
| GESERICK_TERT_TARGETS_DN | -0.2871 | 0.0152 |
| REACTOME_PI3K_EVENTS_IN_ERBB2_SIGNALING | -0.2866 | 0.0154 |
| SUNG_METASTASIS_STROMA_DN | 0.286 | 0.0156 |
| RIZ_ERYTHROID_DIFFERENTIATION_12HR | -0.2856 | 0.0158 |
| KEGG_AXON_GUIDANCE | -0.2853 | 0.0159 |
| KEGG_ENDOCYTOSIS | -0.285 | 0.016 |
| REACTOME_SIGNALING_BY_PDGF | -0.2849 | 0.016 |
| PAPASPYRIDONOS_UNSTABLE_ATEROSCLEROTIC_PLAQUE_DN | -0.2839 | 0.0164 |
| DANG_REGULATED_BY_MYC_UP | 0.2839 | 0.0164 |
| CHEN_LVAD_SUPPORT_OF_FAILING_HEART_UP | -0.2834 | 0.0166 |
| REACTOME_AXON_GUIDANCE | -0.2829 | 0.0168 |
| DAIRKEE_TERT_TARGETS_DN | -0.2819 | 0.0172 |
| WANG_PROSTATE_CANCER_ANDROGEN_INDEPENDENT | -0.2815 | 0.0174 |
| CUI_TCF21_TARGETS_2_DN | -0.2814 | 0.0174 |
| KEGG_DILATED_CARDIOMYOPATHY | -0.2813 | 0.0175 |
| CHOI_ATL_STAGE_PREDICTOR | 0.2809 | 0.0177 |
| KRIGE_RESPONSE_TO_TOSEDOSTAT_6HR_DN | 0.2803 | 0.0179 |
| REACTOME_SIGNALING_BY_FGFR | -0.2792 | 0.0184 |
| REACTOME_NCAM_SIGNALING_FOR_NEURITE_OUT_GROWTH | -0.279 | 0.0185 |
| DAZARD_UV_RESPONSE_CLUSTER_G5 | 0.2787 | 0.0186 |
| PID_NCADHERIN_PATHWAY | -0.2782 | 0.0188 |
| FIGUEROA_AML_METHYLATION_CLUSTER_6_UP | -0.2777 | 0.019 |
| CUI_GLUCOSE_DEPRIVATION | 0.2766 | 0.0196 |
| LANDIS_ERBB2_BREAST_TUMORS_65_DN | -0.2764 | 0.0196 |
| CASTELLANO_NRAS_TARGETS_DN | -0.2764 | 0.0197 |
| YAO_TEMPORAL_RESPONSE_TO_PROGESTERONE_CLUSTER_15 | -0.2762 | 0.0197 |
| BIOCARTA_INTEGRIN_PATHWAY | -0.2759 | 0.0199 |
| PID_ER_NONGENOMIC_PATHWAY | -0.2759 | 0.0199 |
| LEE_NEURAL_CREST_STEM_CELL_DN | -0.2751 | 0.0202 |
| MATZUK_SPERMATOGONIA | -0.2748 | 0.0204 |
| WATANABE_ULCERATIVE_COLITIS_WITH_CANCER_UP | -0.2744 | 0.0206 |
| TAKADA_GASTRIC_CANCER_COPY_NUMBER_DN | -0.2742 | 0.0207 |
| REACTOME_SMOOTH_MUSCLE_CONTRACTION | -0.274 | 0.0207 |
| COLLER_MYC_TARGETS_DN | -0.274 | 0.0208 |
| GENTILE_UV_LOW_DOSE_DN | -0.2738 | 0.0208 |
| NIKOLSKY_BREAST_CANCER_16Q24_AMPLICON | 0.2737 | 0.0209 |
| STARK_HYPPOCAMPUS_22Q11_DELETION_UP | -0.2722 | 0.0217 |
| NIKOLSKY_BREAST_CANCER_12Q24_AMPLICON | 0.2712 | 0.0222 |
| BIOCARTA_RAS_PATHWAY | -0.2692 | 0.0232 |
| ZHAN_MULTIPLE_MYELOMA_CD2_DN | 0.269 | 0.0233 |
| BIOCARTA_MYOSIN_PATHWAY | -0.2689 | 0.0233 |
| DAWSON_METHYLATED_IN_LYMPHOMA_TCL1 | -0.2689 | 0.0234 |
| REACTOME_DOWNSTREAM_SIGNAL_TRANSDUCTION | -0.2687 | 0.0235 |
| PEREZ_TP63_TARGETS | -0.2686 | 0.0235 |
| BIOCARTA_CELL2CELL_PATHWAY | -0.2682 | 0.0237 |
| BIOCARTA_IGF1_PATHWAY | -0.2676 | 0.024 |
| PID_SMAD2_3NUCLEAR_PATHWAY | -0.2675 | 0.0241 |
| BIOCARTA_AGR_PATHWAY | -0.2675 | 0.0241 |
| SOTIRIOU_BREAST_CANCER_GRADE_1_VS_3_DN | -0.2674 | 0.0242 |
| REACTOME_DOWNSTREAM_SIGNALING_OF_ACTIVATED_FGFR | -0.2673 | 0.0242 |
| LEE_AGING_MUSCLE_UP | -0.2669 | 0.0245 |
| BEGUM_TARGETS_OF_PAX3_FOXO1_FUSION_UP | -0.2668 | 0.0245 |
| RICKMAN_TUMOR_DIFFERENTIATED_MODERATELY_VS_POORLY_UP | -0.2665 | 0.0247 |
| FIGUEROA_AML_METHYLATION_CLUSTER_7_UP | -0.2654 | 0.0253 |
| KEGG_GAP_JUNCTION | -0.2648 | 0.0256 |
| BIOCARTA_CCR3_PATHWAY | -0.2643 | 0.026 |
| ST_TYPE_I_INTERFERON_PATHWAY | 0.264 | 0.0261 |
| BIOCARTA_PAR1_PATHWAY | -0.2632 | 0.0266 |
| PID_MAPK_TRK_PATHWAY | -0.2626 | 0.0269 |
| BENPORATH_SUZ12_TARGETS | -0.2624 | 0.027 |
| NIELSEN_GIST | -0.2624 | 0.0271 |
| MARTIN_INTERACT_WITH_HDAC | -0.2622 | 0.0272 |
| TESAR_ALK_TARGETS_EPISC_4D_UP | -0.262 | 0.0273 |
| KEGG_HYPERTROPHIC_CARDIOMYOPATHY_HCM | -0.262 | 0.0273 |
| XU_GH1_AUTOCRINE_TARGETS_UP | -0.2617 | 0.0275 |
| SHIN_B_CELL_LYMPHOMA_CLUSTER_8 | 0.2616 | 0.0275 |
| TOMLINS_PROSTATE_CANCER_DN | -0.2613 | 0.0277 |
| REACTOME_G_ALPHA1213_SIGNALLING_EVENTS | -0.2612 | 0.0278 |
| KRIGE_RESPONSE_TO_TOSEDOSTAT_24HR_DN | 0.2611 | 0.0279 |
| KIM_TIAL1_TARGETS | 0.261 | 0.0279 |
| KEGG_REGULATION_OF_ACTIN_CYTOSKELETON | -0.2609 | 0.028 |
| EHLERS_ANEUPLOIDY_UP | -0.2609 | 0.028 |
| SPIELMAN_LYMPHOBLAST_EUROPEAN_VS_ASIAN_UP | 0.2606 | 0.0282 |
| REACTOME_NUCLEAR_SIGNALING_BY_ERBB4 | -0.2599 | 0.0286 |
| PID_FAK_PATHWAY | -0.2597 | 0.0287 |
| PID_S1P_S1P2_PATHWAY | -0.2597 | 0.0287 |
| REACTOME_ADENYLATE_CYCLASE_INHIBITORY_PATHWAY | -0.2597 | 0.0288 |
| LIU_PROSTATE_CANCER_DN | -0.2594 | 0.0289 |
| REACTOME_ASSOCIATION_OF_LICENSING_FACTORS_WITH_THE_PRE_REPLICATIVE_COMPLEX | 0.259 | 0.0292 |
| BIOCARTA_NFAT_PATHWAY | -0.2588 | 0.0293 |
| REACTOME_THROMBIN_SIGNALLING_THROUGH_PROTEINASE_ACTIVATED_RECEPTORS_PARS | -0.2583 | 0.0296 |
| REACTOME_SIGNALLING_BY_NGF | -0.2583 | 0.0296 |
| SEIKE_LUNG_CANCER_POOR_SURVIVAL | 0.2581 | 0.0298 |
| PID_THROMBIN_PAR1_PATHWAY | -0.258 | 0.0298 |
| GINESTIER_BREAST_CANCER_20Q13_AMPLIFICATION_UP | -0.2573 | 0.0303 |
| RODRIGUES_THYROID_CARCINOMA_POORLY_DIFFERENTIATED_DN | -0.2573 | 0.0303 |
| REACTOME_GAP_JUNCTION_DEGRADATION | -0.2572 | 0.0304 |
| BROWNE_HCMV_INFECTION_24HR_DN | -0.2572 | 0.0304 |
| TIAN_TNF_SIGNALING_NOT_VIA_NFKB | -0.2571 | 0.0305 |
| LEE_NEURAL_CREST_STEM_CELL_UP | -0.2567 | 0.0307 |
| NEBEN_AML_WITH_FLT3_OR_NRAS_DN | 0.2563 | 0.031 |
| NIELSEN_LEIOMYOSARCOMA_DN | -0.2547 | 0.0321 |
| KEGG_TIGHT_JUNCTION | -0.2546 | 0.0322 |
| REACTOME_SIGNALING_BY_EGFR_IN_CANCER | -0.2539 | 0.0326 |
| FIGUEROA_AML_METHYLATION_CLUSTER_1_UP | -0.2531 | 0.0332 |
| INGRAM_SHH_TARGETS | -0.253 | 0.0332 |
| PIONTEK_PKD1_TARGETS_DN | 0.2529 | 0.0334 |
| KEGG_LONG_TERM_DEPRESSION | -0.2527 | 0.0335 |
| THUM_SYSTOLIC_HEART_FAILURE_DN | -0.2523 | 0.0338 |
| KINSEY_TARGETS_OF_EWSR1_FLII_FUSION_DN | -0.2523 | 0.0338 |
| MOOTHA_PYR | -0.2522 | 0.0339 |
| IWANAGA_CARCINOGENESIS_BY_KRAS_DN | -0.2518 | 0.0341 |
| IVANOVA_HEMATOPOIESIS_STEM_CELL_LONG_TERM | -0.2517 | 0.0342 |
| REACTOME_TRAFFICKING_OF_AMPA_RECEPTORS | -0.2514 | 0.0344 |
| KEGG_PENTOSE_PHOSPHATE_PATHWAY | 0.2512 | 0.0346 |
| REACTOME_DEVELOPMENTAL_BIOLOGY | -0.2511 | 0.0346 |
| VANTVEER_BREAST_CANCER_ESR1_DN | 0.2511 | 0.0347 |
| BIOCARTA_MET_PATHWAY | -0.2506 | 0.0351 |
| KEGG_PHOSPHATIDYLINOSITOL_SIGNALING_SYSTEM | -0.2505 | 0.0351 |
| PID_LYMPH_ANGIOGENESIS_PATHWAY | -0.2505 | 0.0351 |
| BROWNE_HCMV_INFECTION_24HR_UP | 0.2504 | 0.0352 |
| ZHAN_MULTIPLE_MYELOMA_LB_UP | -0.2503 | 0.0353 |
| COURTOIS_SENESCENCE_TRIGGERS | 0.2502 | 0.0353 |
| CHEN_HOXA5_TARGETS_6HR_UP | -0.2502 | 0.0353 |
| CUI_TCF21_TARGETS_DN | -0.2499 | 0.0356 |
| REACTOME_FACILITATIVE_NA_INDEPENDENT_GLUCOSE_TRANSPORTERS | 0.2496 | 0.0358 |
| WONG_ENDMETRIUM_CANCER_DN | -0.2496 | 0.0358 |
| REACTOME_CELL_EXTRACELLULAR_MATRIX_INTERACTIONS | -0.2495 | 0.0359 |
| DER_IFN_ALPHA_RESPONSE_DN | -0.2494 | 0.036 |
| DARWICHE_PAPILLOMA_PROGRESSION_RISK | 0.249 | 0.0363 |
| BREUHAHN_GROWTH_FACTOR_SIGNALING_IN_LIVER_CANCER | -0.2489 | 0.0364 |
| REACTOME_NITRIC_OXIDE_STIMULATES_GUANYLATE_CYCLASE | -0.2488 | 0.0364 |
| FIGUEROA_AML_METHYLATION_CLUSTER_3_UP | -0.2485 | 0.0366 |
| LIU_IL13_MEMORY_MODEL_DN | 0.2478 | 0.0372 |
| MCCLUNG_DELTA_FOSB_TARGETS_2WK | -0.2476 | 0.0374 |
| REACTOME_CDC6_ASSOCIATION_WITH_THE_ORC_ORIGIN_COMPLEX | 0.2473 | 0.0376 |
| PID_CDC42_REG_PATHWAY | -0.2472 | 0.0376 |
| VERRECCHIA_RESPONSE_TO_TGFB1_C3 | -0.2472 | 0.0376 |
| HALMOS_CEBPA_TARGETS_DN | -0.2471 | 0.0378 |
| BIOCARTA_HER2_PATHWAY | -0.2469 | 0.0379 |
| RODRIGUES_THYROID_CARCINOMA_ANAPLASTIC_DN | -0.2467 | 0.0381 |
| BIOCARTA_SPRY_PATHWAY | -0.2466 | 0.0382 |
| HU_GENOTOXIC_DAMAGE_24HR | 0.246 | 0.0387 |
| PRAMOONJAGO_SOX4_TARGETS_DN | 0.2455 | 0.0391 |
| GAUSSMANN_MLL_AF4_FUSION_TARGETS_C_UP | -0.2452 | 0.0393 |
| CHESLER_BRAIN_D6MIT150_QTL_CIS | -0.2449 | 0.0396 |
| TAGHAVI_NEOPLASTIC_TRANSFORMATION | -0.2445 | 0.0398 |
| BONOME_OVARIAN_CANCER_SURVIVAL_SUBOPTIMAL_DEBULKING | -0.2445 | 0.0399 |
| SANA_TNF_SIGNALING_DN | -0.2442 | 0.0402 |
| REACTOME_ADENYLATE_CYCLASE_ACTIVATING_PATHWAY | -0.2439 | 0.0404 |
| DANG_MYC_TARGETS_UP | 0.2436 | 0.0406 |
| BURTON_ADIPOGENESIS_10 | -0.2436 | 0.0407 |
| SMID_BREAST_CANCER_LUMINAL_A_DN | 0.2434 | 0.0408 |
| VANTVEER_BREAST_CANCER_BRCA1_UP | 0.2432 | 0.041 |
| AFFAR_YY1_TARGETS_UP | -0.2431 | 0.0411 |
| CHUNG_BLISTER_CYTOTOXICITY_UP | 0.2427 | 0.0414 |
| TURASHVILI_BREAST_NORMAL_DUCTAL_VS_LOBULAR_UP | -0.2427 | 0.0414 |
| HASLINGER_B_CLL_WITH_13Q14_DELETION | -0.2426 | 0.0415 |
| SMID_BREAST_CANCER_RELAPSE_IN_LUNG_DN | -0.2418 | 0.0422 |
| BIOCARTA_CARDIACEGF_PATHWAY | -0.2415 | 0.0425 |
| HUMMERICH_SKIN_CANCER_PROGRESSION_DN | -0.2413 | 0.0426 |
| KEGG_NEUROTROPHIN_SIGNALING_PATHWAY | -0.2412 | 0.0427 |
| REACTOME_HORMONE_SENSITIVE_LIPASE_HSL_MEDIATED_TRIACYLGLYCEROL_HYDROLYSIS | -0.2409 | 0.043 |
| NAKAMURA_LUNG_CANCER_DIFFERENTIATION_MARKERS | 0.2403 | 0.0436 |
| SHIN_B_CELL_LYMPHOMA_CLUSTER_9 | 0.2403 | 0.0436 |
| REACTOME_TAK1_ACTIVATES_NFKB_BY_PHOSPHORYLATION_AND_ACTIVATION_OF_IKKS_COMPLEX | 0.2402 | 0.0436 |
| SHIPP_DLBCL_VS_FOLLICULAR_LYMPHOMA_UP | 0.2401 | 0.0437 |
| REACTOME_ABORTIVE_ELONGATION_OF_HIV1_TRANSCRIPT_IN_THE_ABSENCE_OF_TAT | 0.2399 | 0.0439 |
| MARTENS_TRETINOIN_RESPONSE_DN | 0.2399 | 0.0439 |
| LEE_METASTASIS_AND_ALTERNATIVE_SPLICING_DN | -0.2397 | 0.044 |
| PEREZ_TP53_AND_TP63_TARGETS | -0.2394 | 0.0444 |
| REACTOME_NGF_SIGNALLING_VIA_TRKA_FROM_THE_PLASMA_MEMBRANE | -0.2392 | 0.0445 |
| RAY_TUMORIGENESIS_BY_ERBB2_CDC25A_DN | -0.2392 | 0.0446 |
| BURTON_ADIPOGENESIS_9 | -0.2391 | 0.0446 |
| NAKAMURA_ADIPOGENESIS_LATE_UP | -0.2391 | 0.0446 |
| REACTOME_MRNA_3_END_PROCESSING | 0.239 | 0.0447 |
| VALK_AML_CLUSTER_8 | -0.2386 | 0.0451 |
| KEGG_CYSTEINE_AND_METHIONINE_METABOLISM | 0.2384 | 0.0453 |
| WANG_NFKB_TARGETS | -0.2382 | 0.0455 |
| FIGUEROA_AML_METHYLATION_CLUSTER_2_UP | -0.2381 | 0.0455 |
| REACTOME_REGULATION_OF_INSULIN_SECRETION_BY_ACETYLCHOLINE | -0.2378 | 0.0458 |
| ZERBINI_RESPONSE_TO_SULINDAC_UP | -0.2377 | 0.0459 |
| REACTOME_NEUROTRANSMITTER_RECEPTOR_BINDING_AND_DOWNSTREAM_TRANSMISSION_IN_THE_POSTSYNAPTIC_CELL | -0.2377 | 0.046 |
| KANG_IMMORTALIZED_BY_TERT_DN | -0.2376 | 0.046 |
| CAIRO_LIVER_DEVELOPMENT_UP | -0.2376 | 0.0461 |
| PLASARI_TGFB1_TARGETS_10HR_DN | -0.2375 | 0.0461 |
| CHEBOTAEV_GR_TARGETS_DN | -0.2374 | 0.0462 |
| UZONYI_RESPONSE_TO_LEUKOTRIENE_AND_THROMBIN | -0.2374 | 0.0462 |
| BIOCARTA_INSULIN_PATHWAY | -0.2373 | 0.0463 |
| BIOCARTA_BAD_PATHWAY | -0.2373 | 0.0463 |
| CHICAS_RB1_TARGETS_CONFLUENT | -0.2372 | 0.0464 |
| REACTOME_SEMA4D_INDUCED_CELL_MIGRATION_AND_GROWTH_CONE_COLLAPSE | -0.2364 | 0.0472 |
| PICCALUGA_ANGIOIMMUNOBLASTIC_LYMPHOMA_DN | -0.2363 | 0.0473 |
| PID_BETA_CATENIN_NUC_PATHWAY | -0.2362 | 0.0474 |
| ZEMBUTSU_SENSITIVITY_TO_FLUOROURACIL | 0.2357 | 0.0479 |
| WANG_RESPONSE_TO_PACLITAXEL_VIA_MAPK8_DN | -0.2357 | 0.0479 |
| NAGASHIMA_EGF_SIGNALING_UP | -0.2355 | 0.048 |
| HEDENFALK_BREAST_CANCER_BRACX_DN | 0.2352 | 0.0484 |
| WONG_ADULT_TISSUE_STEM_MODULE | -0.2351 | 0.0485 |
| MCCLUNG_CREB1_TARGETS_UP | -0.235 | 0.0486 |
| ODONNELL_METASTASIS_UP | -0.2345 | 0.049 |
| BIOCARTA_BARR_MAPK_PATHWAY | -0.2343 | 0.0493 |
| BAKKER_FOXO3_TARGETS_DN | 0.2341 | 0.0494 |
| REACTOME_SIGNALING_BY_FGFR_IN_DISEASE | -0.234 | 0.0495 |
| GOBERT_OLIGODENDROCYTE_DIFFERENTIATION_DN | -0.2337 | 0.0498 |
| UDAYAKUMAR_MED1_TARGETS_DN | -0.2337 | 0.0498 |
| KANG_AR_TARGETS_UP | -0.2337 | 0.0498 |
