# Supplementary material for: A novel genomic signature predicting FDG uptake in diverse metastatic tumors
Source: EJNMMI Res. 2018 Jan 18;8:4. doi: 10.1186/s13550-017-0355-3 (PMC5773462; doi:10.1186/s13550-017-0355-3)
Supplement: Supplementary file 7 — Characteristics of the patients in the validation set along with their measured and predicted (SUVPLS) SUV values. (DOCX 13 kb) [file 13550_2017_355_MOESM7_ESM.docx]

**Table S3.** Characteristics of the patients in the validation set along with their measured and predicted (SUVPLS) SUV values.

| **Patient number** | **Histology** | **Biopsy Localization** | **SUVmeasured** | **SUVPLS** |
| --- | --- | --- | --- | --- |
| Patient 1 | Lung cancer | Lung | 4.09 | 6.02 |
| Patient 2 | Kidney cancer | Lymphadenopathy | 8.36 | 5.40 |
| Patient 3 | Pancreatic cancer | Primary | 5.18 | 3.45 |
| Patient 4 | Bile duct cancer | Primary | 10.74 | 6.11 |
| Patient 5 | Breast cancer | Lymphadenopathy | 8.62 | 7.30 |
| Patient 6 | Pancreatic cancer | Liver | 8.02 | 7.92 |
| Patient 7 | Ovarian cancer | Retroperitoneal | 6.87 | 6.18 |
| Patient 8 | Breast cancer | Lymphadenopathy | 4.93 | 6.90 |
| Patient 9 | Genitourinary tumor | Lymphadenopathy | 1.96 | 6.43 |
| Patient 10 | Ovarian cancer | Retroperitoneal | 8.83 | 7.76 |
| Patient 11 | Esophageal cancer | Retroperitoneal | 3.96 | 4.13 |
| Patient 12 | Bile duct cancer | Retroperitoneal | 3.38 | 4.60 |
| Patient 13 | Ovarian cancer | Retroperitoneal | 9.95 | 7.30 |
